# Dynamics of temperature-actuated droplets within microfluidics

**DOI:** 10.1038/s41598-019-40069-9

**Published:** 2019-03-07

**Authors:** Asmaa Khater, Mehdi Mohammadi, Abdulmajeed Mohamad, Amir Sanati Nezhad

**Affiliations:** 1 Department of Mechanical and Manufacturing Engineering, University of Calgary, Calgary, AB T2N 1N4 Canada; 2 BioMEMS and Bioinspired Microfluidic Laboratory, Department of Mechanical and Manufacturing Engineering, University of Calgary, Calgary, AB T2N 1N4 Canada; 3 Centre for Bioengineering Research and Education, University of Calgary, Calgary, AB T2N 1N4 Canada

## Abstract

Characterizing the thermal behavior of dispersed droplets within microfluidic channels is crucial for different applications in lab-on-a-chip. In this paper, the physics of droplets volume during their transport over a heater is studied experimentally and numerically. The response of droplets to external heating is examined at temperature ranges of 25–90 °C and at different flow rates of the dispersed phase respect to the continuous flow. The results present a reliable prediction of the droplet volume and stability when heating is applied to the droplets at the downstream channel in a quite far distance from the droplets’ ejection orifice. Increasing the ratio of flow rate resulted in larger droplets; for instance, the flow ratio of 0.25 produced drops with 40% larger diameter than the flow rate of 0.1. For every 10 °C increase in temperature of the droplets, the droplet diameter increased by about 5.7% and 4.2% for pure oil and oil with a surfactant, respectively. Also, the droplets showed a degree of instability during their transport over the heater at higher temperatures. Adding SPAN 20 surfactant improved the stability of the droplets at temperatures higher than 60 °C. The experimentally validated numerical model helped for systemic analysis of the influence of key temperature-dependence parameters (e.g. surface tension, density and viscosity of both phases) on controlling the volume and stability of droplets. Our findings supported to develop highly functional systems with a predetermined droplets performance under high temperatures up to 90 °C. This report provides a preliminary basis for enhancing the performance of droplet microfluidic systems for digital droplet polymerase chain reaction (ddPCR), continuous flow digital loop-mediated isothermal PCR (LAMP), and droplet-based antibiotic susceptibility testing.

## Introduction

Droplets and threads with the ability to conduct a small volume of fluids scaled from nano- to microliter became promising for different applications, including tissue engineering^1^, particle-based display technologies^2^, therapeutics^3^, high performance composite filler materials^4^, and food industry^5^. Monodispersed droplets can operate as microreactors not only for performing reactions in parallel, series, or parallel/series combinations^6,7^ but also to implement multiple reactions by changing the reaction conditions within each droplet. DNA amplifications^8^, single-cell assays^9,10^, cell-free protein analysis^11^, and synthesis of nanocrystals^12,13^ are examples of the applications that require precise thermal control or rapid switching between different temperatures. The high surface-area-to-volume ratio offered by microdroplets ensures a fast response to the change in temperature. The challenges such as evaporation, limited high-throughput performance, and uncontrolled diffusion under external heating have been primarily confronted by emulsion-based microfluidics. However, the temperature dependency of droplets’ physical properties represented by the variation in viscosities, densities and surface tension between the carrier and dispersed phases have complicated the high-performance droplet microfluidics under exposure to external heating sources. Controlling the stability of droplet transport at higher temperatures remain challenging.

The temperature dependency of the interfacial and viscosity properties of water-in-oil drops subject to thermal energy of a heater placed at the breakup location has been investigated in flow-focusing devices^14^ and T-junction systems^15–19^. Stan *et al*.^20^ used the temperature regulation of inlet channels and nozzles of a flow-focusing device to control velocity and volume of the produced droplets. Also, droplet sorting and transport through symmetrical micro-bifurcations using laser and electrical resistance heating were reported by Baroud *et al*.^21^ and Yap *et al*.^22,23^, respectively. Verneuil *et al*.^24^ and Yesiloz *et al*.^25^ used a localized heating at the downstream channel as an effective method for rapid mixing inside individual droplets. The former employed a tightly focused infrared laser on droplets formed in a T-junction geometry, while the latter proposed an integrated microwave-based heater in a flow focusing generator. In a recent study by Lee *et al*.^26^, a control system was developed to dynamically measure the temperature-dependent interfacial tension based on the drop deformation and by embedding an integrated localized heating system placed in a series of T-junction, co-flowing, and contraction-expansion configurations. All these works have studied the effect of temperature on the generation and transport of liquid droplets, however to the best of our knowledge, no work has been performed on the behavior of drops in response to a heating source placed far from the droplet ejection point. Also, the highest reported temperature applied to droplets was 80 °C^20^, however, a temperature of up to 95 °C is needed for applications like polymerase chain reaction (PCR)^27,28^.

On the other hand, numerical analysis of droplet transport under external heating is very complicated and computationally extensive due to the need to 3D simulating of droplets^29,30^. Most of the numerical analysis of droplet formation and transport are limited to 2D simulations aiming to investigate heat transfer enhancement and characterize internal circulations within the droplets (Marangoni effect)^31–35^. To the authors’ knowledge, the only 3D numerical model of droplet formation in a heated T-junction microchannel was presented by Ho *et al*.^36^, where they considered the effect of temperature-dependant properties and thermos-coalescence on droplet transport. It is noticeable that the physics governing the thermo-microfluidics require further understanding due to the strong coupling of different physical phenomena.

In this work, the physics of droplet transport subject to an external heating source placed at the downstream of the transport channel is studied experimentally and numerically in a flow-focusing device. Monodispersed droplets by a flow-focusing system are first produced relying on the models of Ganan-Calvo and coworkers^37,38^ and Anna *et al*.^39^. The stretch of dispersed phase by the continuous phase at the nozzle breaks it into droplets. An external heater is placed underneath the downstream transport channel at a distance far from the nozzle to guarantee that the heating energy does not affect the upstream droplet formation channels. The effect of thermal energy on the size of droplets during their transport over the heater is investigated at the temperature range of 25–90 °C. This thermal influence is also studied in the presence and absence of surfactants added to the continuous phase. A 3D thermofluid numerical model is developed for simulating the physics of droplets transport through the downstream microchannel where the droplets are subject to external heating. The numerical model is then used to interpret the size (volume) change of droplets under the thermal effect. The outcome of the present work benefits future works for optimizing the performance of droplet-based DNA sequencing, including digital droplet polymerase chain reaction (ddPCR)^27,28^ and continuous flow digital loop-mediated isothermal PCR (LAMP)^40^. While the target DNA sequence in the PCR process is exponentially amplified through successive thermal cycles alternating between two or among three different temperatures for specific time intervals^41,42^ the LAMP technique involves DNA amplification at a single temperature^43^. These two techniques require a highly precise thermal reaction on droplets subject to different thermal sources during their transport to eliminate the detection of false positive results.

## Results and Discussion

### Device design

**Figure 1:**
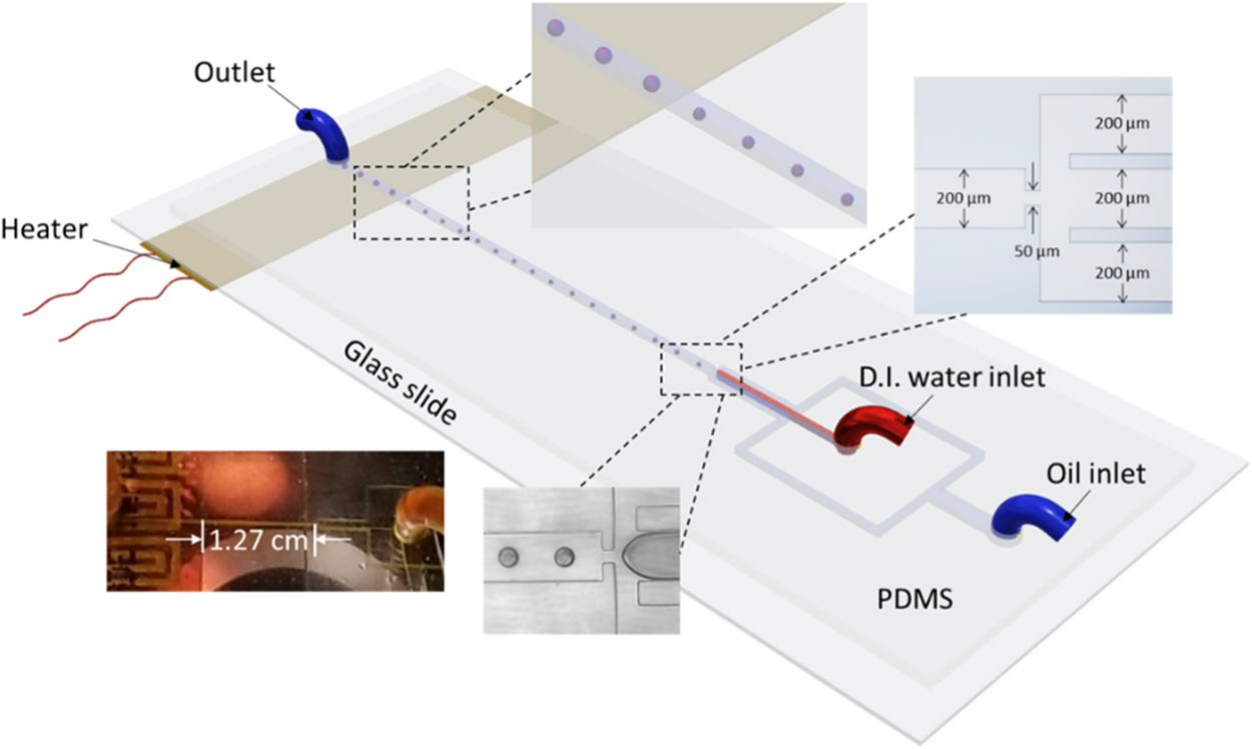
The schematic presentation of the microfluidic droplet chip and the integrated heating system. The heater is located 1.27 cm away from the site of droplet generation (nozzle).

The microchannels network shown in Fig. 1 comprises two inlets, droplet generation zone (nozzle) with carrier microchannels, one downstream channel and one outlet. The width of the carrier microchannels and the downstream channel is 200 μm, and the thickness of polydimethylsiloxane (PDMS) walls separating the continuous and the dispersed channels is 150 μm. The width and length of the focusing nozzle are 50 μm and 70 μm, respectively. All channels have 80 μm depth. The bottom surface of the glass slide that seals the microfluidic system is in direct contact with a flexible pressure-sensitive adhesive (PSA) heater. The heater was placed 1.27 cm away from the nozzle site. The heater is 1 cm wide and 5 cm long (OMEGA Engineering Inc, Kapton® (Polyimide Film), insulated heater KHLV Series, 28 Volts).

### Experimental tests and validation

The flow rate of DI water was kept constant at 100 μl/hr for all experiments and the droplet characterization was examined at four different flow rate ratios, ${\boldsymbol{\phi }}=\frac{{{\boldsymbol{Q}}}_{{\boldsymbol{d}}}}{{{\boldsymbol{Q}}}_{{\boldsymbol{c}}}\,}$,  of 0.1, 0.15, 0.2, 0.25 and at different temperatures, T = 25, 40, 50, 60, 70, 80, 90 °C. This range of temperature was tested to consider all possible operating temperatures of PCR and LAMP. The heater is turned on for 20 min prior to recording the results. First, the effect of adding SPAN 20 to the carrier phase on the size and stability of droplets was investigated. For each data point, fifteen measurements were recorded for effective droplets diameter, and images are captured at three different locations; (i) the droplet-formation site to guarantee that the heat energy dissipated from the heater has no impact on droplets diameter at the generation zone, (ii) a transition-zone that considers the droplets behavior at the beginning of heating site to track the instant response of droplets to temperature change, and (iii) heater zone to record the droplet size.

**Figure 2:**
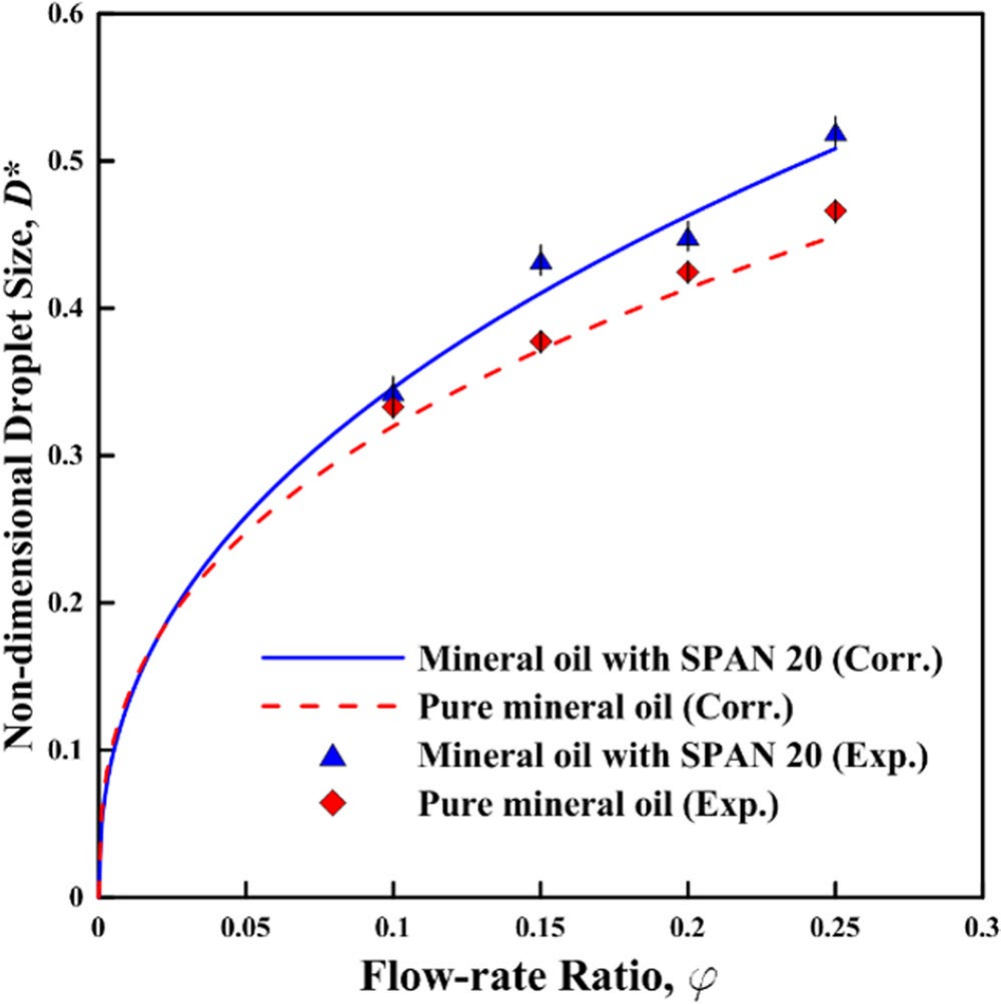
The normalized droplets diameter (D*) as a function of the flow rate ratios (φ) at 25 °C with the curves of the fitting correlations (Maximum relative error is 1.5%).

Figure 2 indicates the variation of the normalized droplets diameter ${D}^{\ast }$ against the flow rate ratios $\phi$ at 25 °C. Adding SPAN 20 results in about 1.1 times larger droplets in diameter at the same flow rate ratio, which agrees with the data in the literature^44^. This is physically sound as the surfactant reduces the interfacial tension between the immiscible phases. The droplets diameter is zero for the flow-rate ratio of $\phi =0$ as the no-flow condition. The least-squares method for power function was implemented to obtain the correlation in equation (1) (a maximum relative error less than 1.5%) and predict the droplets size as a function of flow rate ratios.

1 $${D}^{\ast }=\{\begin{array}{l}0.75{\phi }^{0.37},{\rm{pure}}\,{\rm{Oil}}\\ 0.83{\phi }^{0.37},{\rm{Oil}}\,{\rm{with}}\,{\rm{SPAN20}}\end{array}$$

**Figure 3:**
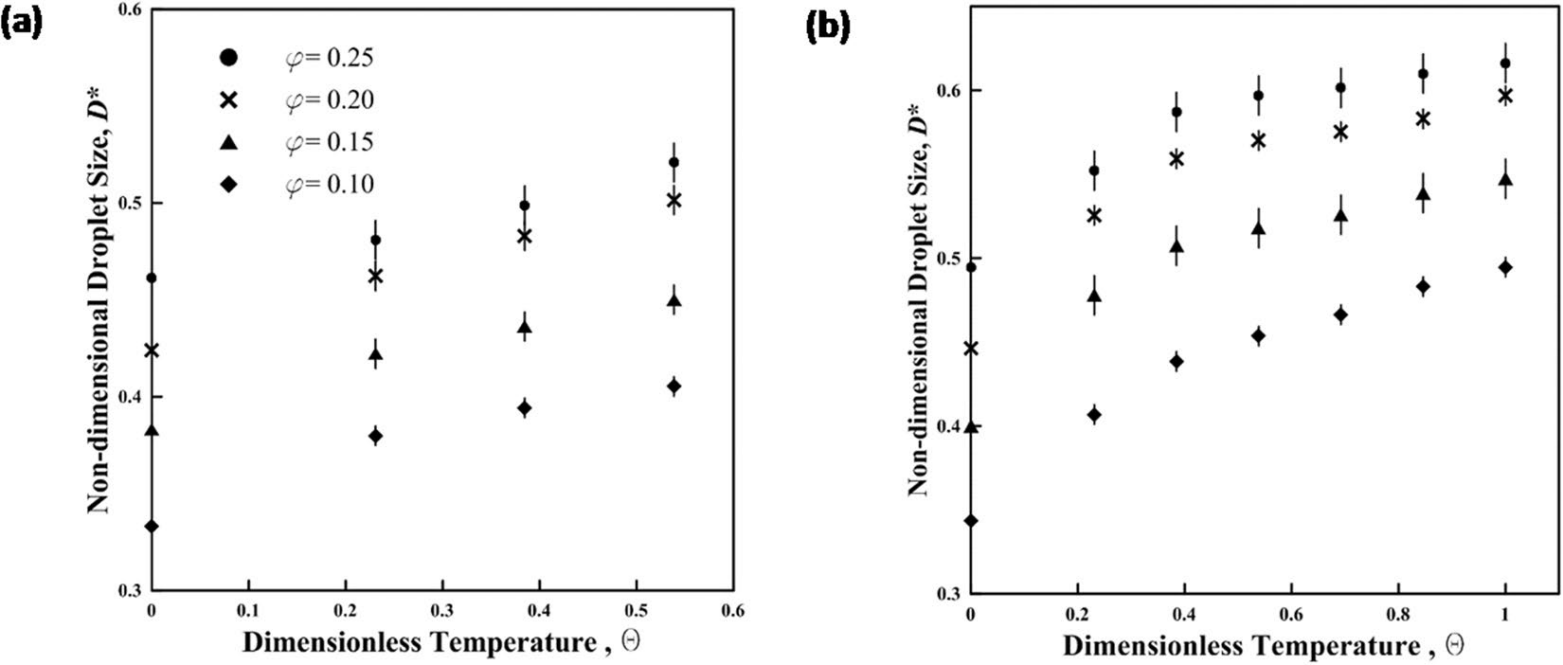
The varation of normalized droplets diameter (D*) with dimensionless temperature $({\theta })$. The continous phase is light minral oil (**a**) without SPAN20 and (**b**) with SPAN20.

The non-dimensional results in Fig. 3 show that the droplet diameter increases with the increase in temperature due to the effect of temperature on the density of the aqueous phase. The percentage of reduction in the density is equal to the percentage of the increase in droplet volume.

**Figure 4:**
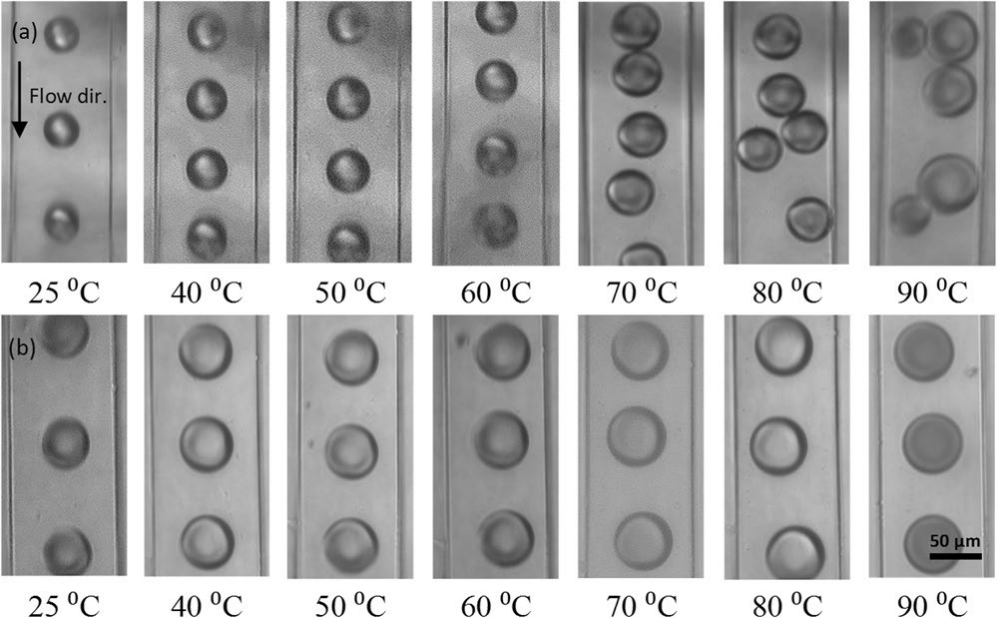
Typical phase diagrams of the change in droplets subject to different temperatures over the heater at the downstream channel (**a**) in the absence of surfactant to the carrier phase and (**b**) in the presence of SPAN20 surfactant. The flow rates of the light mineral oil and D.I. water for (**a**,**b**) cases are Q_c_ = 500 μl/h and Q_d_ = 100 μl/h, respectively. The frames are taken at 15.4 frames per second (fps).

The stability of droplets in the absence of surfactant reduces where they are exposed to temperatures above 60 °C over the heater placed at the downstream channel (Fig. 4a and Movie S1). The results of Fig. 4b for typical phase diagrams at different temperatures show that adding SPAN20 surfactant improves the stability of droplets at higher temperatures mainly due to the increase in the resistance of water droplets against coalescence^45,46^. At each flow rate and at room temperature (Tables S1 and S2) adding SPAN 20 surfactant with 1 CMC concentration not only increases the droplet size but also rises the distance between two successive droplets. On the other hand, increasing the temperature decreases the distance between successive droplets. This distance in the absence of surfactants reduces dramatically and increases the risk of droplets coalescence (the coalescence of droplets is observed at 90 °C). The droplets flowing over the heater remain stable in the presence of surfactant at even 100 °C temperature. As a result of the instability of droplets at high temperatures in the absence of surfactants, the phase diagrams in Fig. 4 are presented at the stable range of droplets. Additional phase diagrams particularly for temperatures higher than 60 °C are presented in Tables S1 and S2.

Since the volumetric ratio of flow rate is independent from the temperature, the correlation relating the non-dimensional droplet diameter is obtained in the form of a product of two functions in equation (2) (flow rate ratio and the non-dimensional temperature).

2 $${D}^{\ast }={f}_{1}(\phi ){f}_{2}(\theta )$$

where the first function ${f}_{1}(\phi )\,\,$is deduced from equation (1) at room temperature. The values of droplets diameter at $\theta =0$ are defined at room temperature (25 °C). Since the initial value of ${f}_{2}(\theta )$ is non-zero, an exponential function or a 2^nd^ degree polynomial is applied to satisfy question (2). Applying the least-squares method to fit the exponential function ${f}_{2}(\theta )$ shows a maximum relative error of 6.7% and an average relative error of 3.2%, while for the 2^nd^ degree polynomial ${f}_{2}(\theta )$, the maximum and the average relative error are 7.7% and 3.7%, respectively. Tables S3 and S4 show that the correlation fits very well with the experimental results. We select the exponential function as the fitting curve, and ${f}_{2}(\theta )$ is formulated as equation (3).

3 $${f}_{2}(\theta )=\frac{{D}^{\ast }}{{f}_{1}(\phi )}=\{\begin{array}{l}1.01{e}^{0.36\theta },{\rm{pure}}\,{\rm{Oil}}\\ 1.05{e}^{0.27\theta },{\rm{Oil}}\,{\rm{with}}\,{\rm{SPAN}}20\end{array}$$

The final correlation is obtained after substituting *f*_1_ and *f*_2_ in equation (2) with the functions in equations (1) and (3), respectively.

4 $${D}^{\ast }=\{\begin{array}{l}0.76{\phi }^{0.37}{e}^{0.36\theta },{\rm{pure}}\,{\rm{Oil}}\\ 0.87{\phi }^{0.37}{e}^{0.27\theta },{\rm{Oil}}\,{\rm{with}}\,{\rm{SPAN}}20\end{array}$$

The correlation is valid for temperatures up to 60 °C in pure oil tests given the instability of droplets subject to higher temperature over the heater. However, the correlation is valid for temperatures up to 100 °C when SPAN20 is added to the carrier phase.

### Numerical modeling of droplet microfluidic

**Figure 5:**
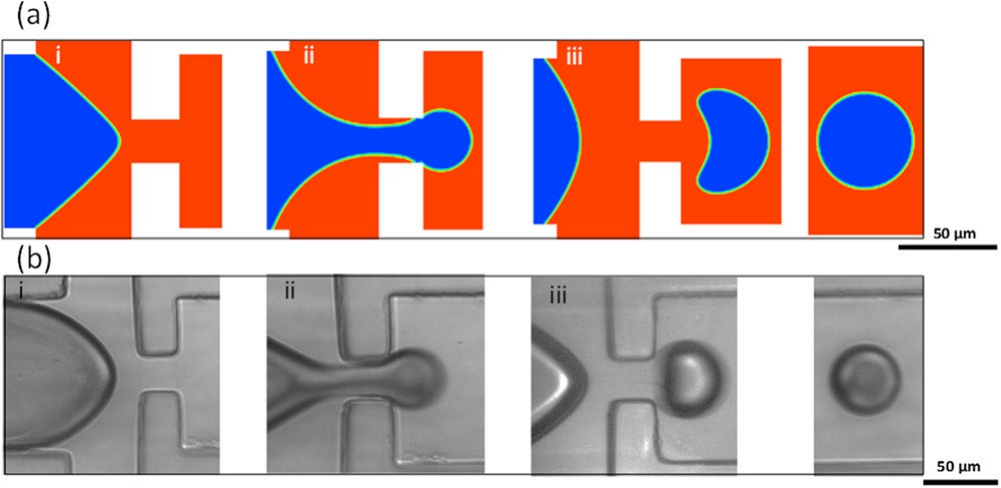
Images from (**a**) simulation and (**b**) experimental results for the droplets (i) formation, (ii) break up and (iii) transport.

ANSYS-Fluent software was used to perform 2D and 3D multiphase flow numerical simulations for droplet generation and transport under thermal effects. A mesh-independency test was conducted to validate the numerical model. The mesh sizes of 2 μm × 2 μm, and 5 μm × 5 μm × 5 μm was selected for each cell in 2D and 3D simulations, respectively. 2D simulations are sufficient to predict reliably the effective diameter of the generated droplets. However, 2D models are not satisfactory to foresee the droplet behaviour in response to external heating. Newtonian fluids as a continuous and dispersed phases were introduced into a rectangular microchannel at 25 °C with a uniform velocity fixed at 1.73 mm/s for the dispersed flow and ranged from 3.47–8.68 mm/s for each inlet of the continuous phase. A gauge pressure of zero is applied at the outlet of the computational domain. Our simulation is limited to testing the multiphase flow in the absence of surfactant. The contact angle is assumed to be constant with a value of 135° measured for PDMS. The thickness of PDMS and glass layers are 3.5 mm and 1.2 mm, respectively, and are set to free convection with a heat transfer coefficient of 10 W/m^2^ K^47,48^. The temperature of the heater surface is adjusted within the range of 50–90 °C. The results of flow patterns are shown in Fig. 5 at the flow rates of 100 and 500 µl/h for the dispersed and continuous phases, respectively. The dispersed phase is released from the orifice followed up with the droplet growth, break up and transport through the downstream channel. The droplet diameter predicted numerically are 3% smaller than the experimental results.

### Lumped system analysis

The lumped capacity method, (equation 5), is used to estimate the time required for droplets to reach the temperature of the heater and compare it with the time calculated from simulations^47,48^.

5 $$\frac{T(t)-{T}_{\infty }}{{T}_{i}-{T}_{\infty }}={e}^{-[\frac{hA}{\rho \forall C}]t}$$

where ${T}_{\infty }$ is oil temperature, ${T}_{i}$ is droplet initial temperature, $h$ is convective heat transfer coefficient, $A$ is drop surface area, $\forall$ is droplet volume, $\rho$ and $C$ are droplet density and specific heat, respectively. The period required to heat up the droplet is inversely proportional to the droplet diameter. The period calculated to heat up the largest drop (≈100 µm) are 28 ms, and 26.3 ms based on the lumped system analysis and numerical simulations, respectively. Figure S2 illustrates the time required to heat up different sizes of the experimentally obtained diameters.

### 3D Simulation of temperature effect on droplets behavior

**Figure 6:**
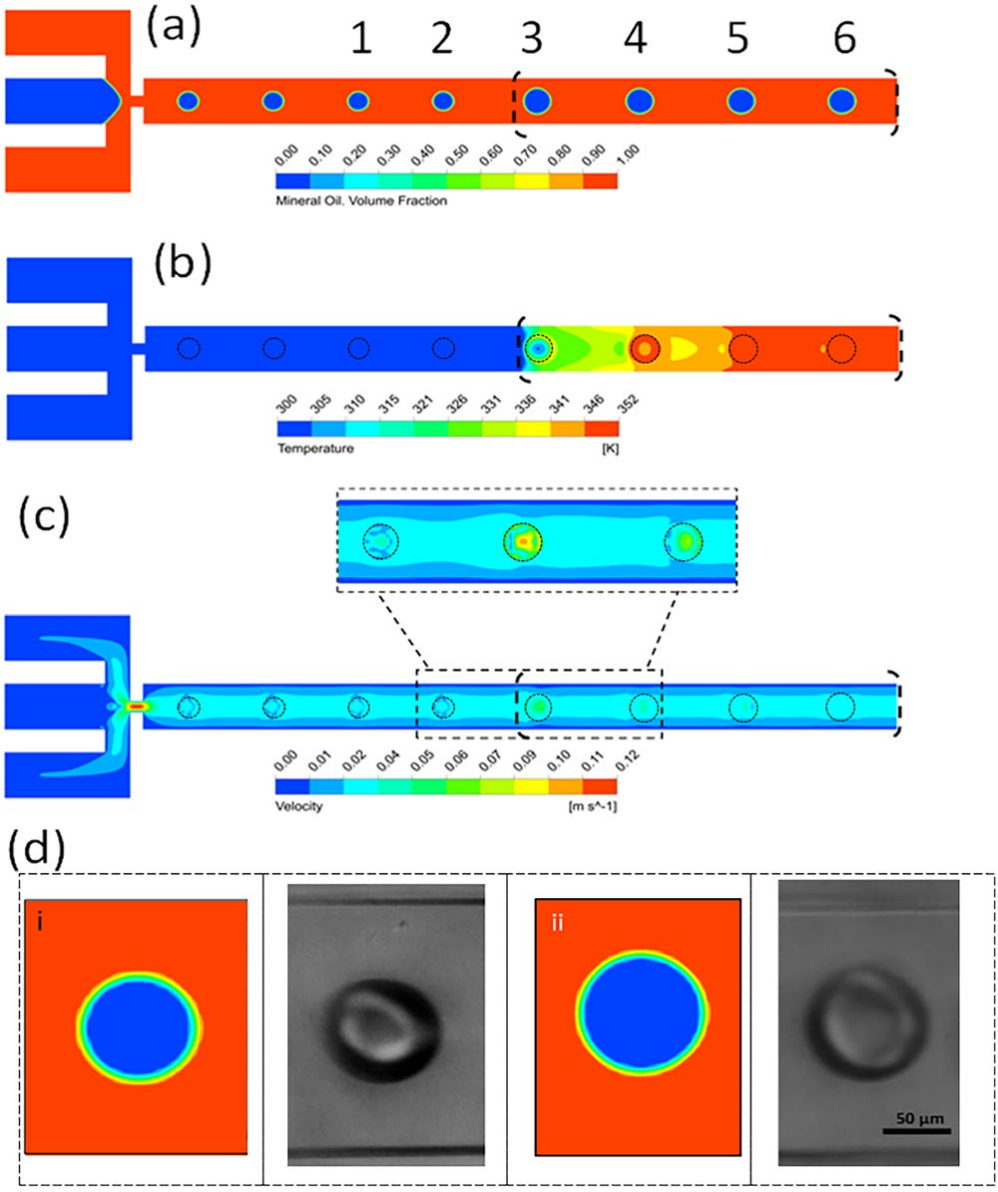
The 3D simulation results of the droplet behavior in response to external thermal stimulation. (**a**) The effective diameter of droplets increases as it passes over the high temperature zone along the flow direction. The size of droplets #3 and #4 in Fig. 8a appears larger in 2D illustration than the actual size due to the thermocapillary effect, however the actual 3D droplet volume and its gradual increase to the temperature gradient over the heater is shown in Figs S4, S5. (**b**) The temperature distribution along with the flow direction indicates the pattern of high temperature zone. (**c**) The velocity profiles illustrating the vortices in the transient condition between the cold and hot zones. (**d**) The images of droplets from both simulations and experiments i) before applying heat at 25 °C and ii) after heating to 60 °C (flow rate ratio = 0.2).

3D numerical simulations were carried out to systemically analyze the effect of different physical properties, including the density, viscosity and surface tension on droplets behaviour. The change of surface tension with the temperature resulted in droplet deformation without any change in droplet volume. The constant droplet volume was calculated based on the mass conservation principle and used to validate the numerical model and the solver. The higher density and viscosity of the carrier fluid resulted in a higher instability of droplets and a thicker layer of the interface between phases. Incorporation of time-dependent physical parameters into the numerical model resulted in a quantitative agreement with the experiments (Fig. 6, Figs S3, S4, and Movie S2). The results of droplet transport at an iso-depth of 40 µm is shown in Fig. 6 where the black dashed line shows the heater boundary. The droplet volume change is traced numerically by quantifying the number of cells per droplet through its travel over the heater (Fig. S5). Although the droplet’s effective diameter calculated from numerical studies is about 2.89% smaller than the effective diameter measured in experiments, the numerically predicted volume (effective diameter) of the droplets showed an increase of 35.3% numerically compared with 35.6% experimentally. Table 1 compares the droplets volume obtained from experimental and numerical data at two different temperatures 25 °C (site of droplet generation) and 80 ^ο^C (over the heater) for water and pure oil at flow rate ratios are 0.1 and 0.2, respectively. The numerical simulations predict precisely the change in temperature and volume of droplet but not simply able to simulate the coalescence of droplets at high temperatures. Moreover, the numerical results show that the maximum velocity of the fully developed flow has increased by 4.13%, and vortices appear inside the droplets as they move from the low to high temperature zone (Fig. 6c). These vortices disappear when the temperature of the droplets reaches the temperature of the surrounding oil. Figure 6d illustrates the change in the drops size numerically and experimentally at the flow rate ratio of 0.2.

**Table 1:** Comparison between droplet volume obtained numerically and experimentally at two different flow rate ratios at temperatures of 25 °C and 80 °C.

${\boldsymbol{\phi }}$	${\boldsymbol{\theta }}$	D^*^ (Experimental)	D^*^ (Numerical)	Error (%)	% D^*^ increase after heating (numerical)	% D^*^ increase after heating (experimental)
0.1	0.00	0.324	0.316	2.47	28.53	29.06
	0.84	0.418	0.406	2.87		
0.2	0.00	0.418	0.407	2.67	35.30	35.61
	0.84	0.568	0.552	2.89		

From previously discussed results and the literature work^17,20^, the droplet volume increased by rising the temperature regardless of the location of the heater. However, the increase in droplet volume when the heater is mounted to the droplet generation nozzle was approximately 2.5 times compared to the increase when the heater was attached to the downstream channel. This could be explained as the surface tension in the former has a dominant effect in forming and controlling the droplet size rather than in the present work where the surface tension only affects the droplets deformation and motion with no effect on their size. Based on the numerical results, the behaviour of droplets moving towards the heater (Fig. S7) when the surface tension and viscosity are functions of temperature agreed with the results presented by Ho *et al*.^36^.

## Conclusion

The work reports the effect of temperature on the dynamic characteristics of droplets flowing in microchannels. Light mineral oil was used as a continuous phase at different flow rate ratios with the DI water as a dispersed phase. Incorporation of surfactant SPAN 20 with the concentration of 1 CMC increased the size of droplets by an average factor of 1.1, meanwhile improved the stabilization of droplets at temperatures higher than 60 °C. The droplet’s effective diameter increased by about 5.7% ± 0.3% and 4.2% ± 0.4% for pure oil and oil with a surfactant, respectively, for every 10 °C increase in the temperature. Also increasing the temperature from 25 °C to 60 °C increased the velocity of droplets by 4.14% ± 0.25%. The correlations that could precisely predict the droplet size were developed as a function of the flow rate ratios and temperature with a maximum relative error of about 1.5% and 6.7%, respectively. Finally, the numerical simulations using the volume of fluid (VOF) method were successfully implemented to validate the experimental results and determine the effect of using temperature dependent properties of phases on the change in droplet size and temperature. The results also provided a framework for understanding the physics underlying the multiphase flow in microsystems when an external heat source is involved. Controlling the dynamic stability of droplets under different temperatures opens up an avenue droplet-based applications in lab-on-a-chip biosensing, chemical reactions and high-throughput omics assays. Further numerical and experimental works are needed to investigate the droplet response to multiple heaters with different temperatures in order to study the adaptability of droplets to the rapid change in temperature; and to use various pairs of continuous and dispersed phases (Newtonian or non-Newtonian fluids) in order to investigate the stability of droplets at temperature in the range of 35–200 °C.

## Experimental Section

### Device fabrication

The photolithography technique was utilized to fabricate the SU-8 mold. The microfluidic chip was fabricated using soft-lithography technique^49^. Briefly, polydimethylsiloxane (PDMS) and curing agent (Sylgard 184 from Dow Corning) were thoroughly mixed with a ration of 10:1 to fabricate the microfluidic chip^49^. The mixture was then degasified in a vacuum chamber for 10 min to eliminate trapped air bubbles in the PDMS sample. The SU-8 mold was silanized to peel-off PDMS easily once cured. The PDMS mixture was poured onto the SU-8 mold and cured in an oven for 8 hrs at 120 °C. The cured PDMS layer was punched to create the inlets and outlet holes and then bonded to a regular microscope slide glass (VWR international Inc.) through standard O_2_ plasma activation (Electro-Technic Products). The glass substrate was cleaned with acetone before bonding to the PDMS layer.

### Materials and experimental setup

**Figure 7:**
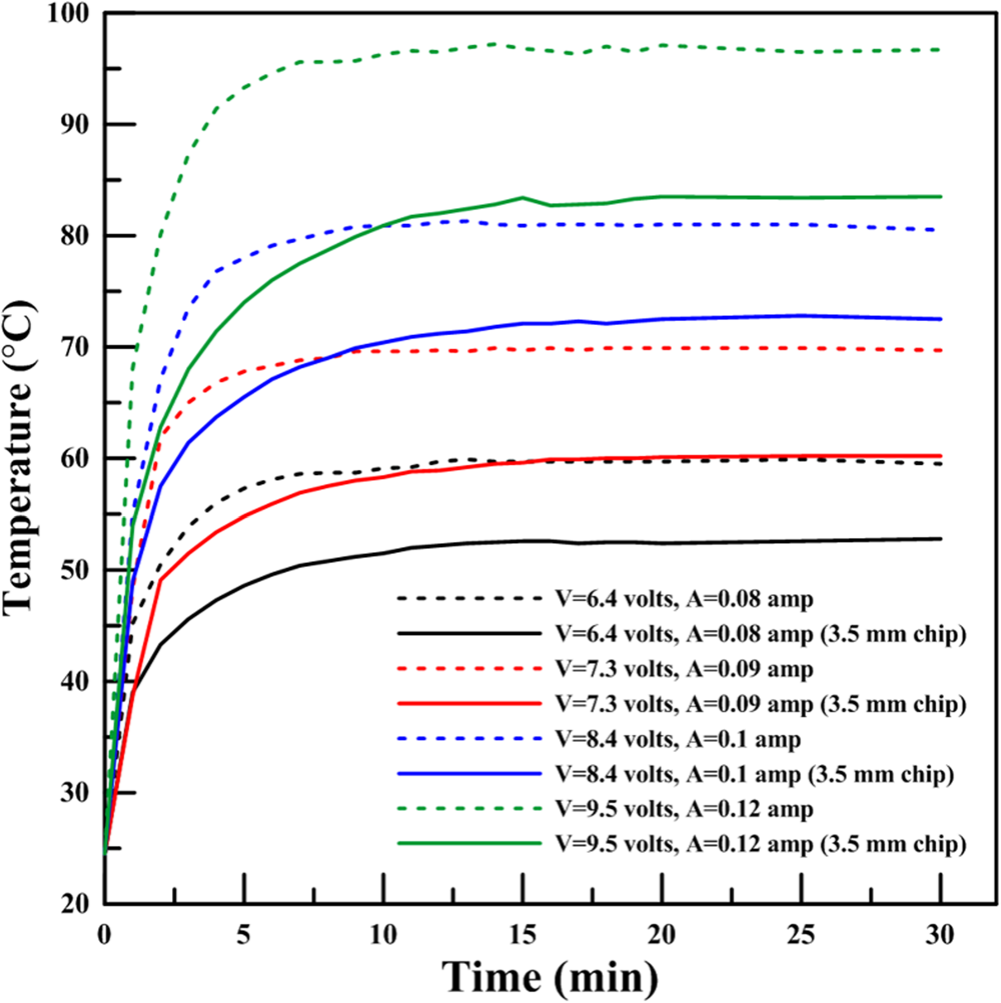
Heater calibration curve in the presence of a microfluidic chip placed perpendicular to the heater (maximum relative error is less than 0.5%).

The continuous phase was light mineral oil (Sigma-Aldrich 330779) and the dispersed phase was DI water with 1% w/w fluorescence dye (Cole Parmer, Canada). The corresponding experiments were conducted in the presence and absence of Sorbitan monolaurate (SPAN 20) with 1 CMC surfactant (Sigma-Aldrich 85544) added to mineral oil. Tygon Microbore tubing (1/32″ID x 1/16″OD, Cole-Parmer Canada) was connected to two programmable precision syringe pumps (Harvard Apparatus PHD2000) and used to continuously inject the oil and aqueous fluid into the carrier micro-channels. The heater was a 1 cm wide and 5 cm long (OMEGA Engineering Inc, Kapton® (Polyimide Film), insulated heater KHLV Series, 28 Volts). The heater was calibrated up to 95 °C in the presence of the microfluidic device placed on its top surface prior testing the droplet generation, for evaluating the heat transfer rate and temperature gradient in the direction normal to the heater Fig. S1. The voltage of the heater was changed using a DC power supply (TENMA 72-8335 A) to adjust the temperature of the chip. The temperature was monitored continuously by means of a precisely calibrated infrared (IR) camera (FLIR T-350). The time-dependent change of temperature within the downstream microchannel over a period of 30 min in response to different voltages is shown in Fig. 7. Upon setting the voltage, the temperature changes rapidly first and its rate slows down until becoming plateau at about 15 min. The tests for obtaining the calibration curve was repeated 5 times. Figure 7 represents the average of these calibrations. The maximum relative error between the average temperature and any other temperature at the same time remained below ±0.5%.

**Figure 8:**
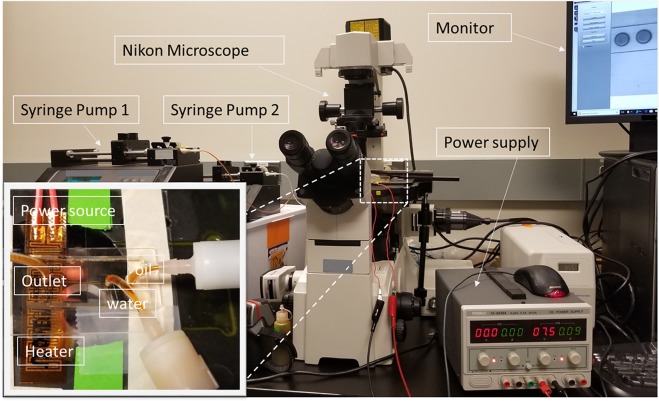
A photograph of the experimetal set-up, including two-syringe pumps, a microscope with a fast recording camera connected to a monitor, infrared (IR) camera, and DC power supply.

The droplets were observed by filter-set and inverted fluorescent microscopes (Nikon EclipseTE2000-S; Nikon Instruments, Melville, NY). A highly sensitive monochromatic CCD camera (Moticam Pro 285 A, Motic, Hong kong) was employed to capture the droplet images which were then processed by NIH ImageJ software (version 1.8.0) to determine the size and shape. A photograph of the experiment setup is presented in Fig. 8.

### Theoretical analysis

The volume of fluid (VOF) numerical method was used in this work for 2D and 3D simulating the effect of temperature on the dynamic droplet size and motility within microchannels. The VOF method, proposed by Hirt and Nichols^50^, can trace the interface between two immiscible phases by solving the phase indicator function ${\boldsymbol{\mbox{''}}}{\boldsymbol{\alpha }}{\boldsymbol{\mbox{''}}}$ in which the volume fraction of the continuous phase ${{\boldsymbol{\alpha }}}_{{\boldsymbol{c}}}$ is obtained numerically by solving $\frac{\partial {\boldsymbol{\alpha }}}{\partial {\bf{t}}}+{\bf{U}}.\nabla {\boldsymbol{\alpha }}\,=0$, where U is the flow velocity field shared by two fluids. The volume fraction of the dispersed phase is directly computed from $(1-\,{{\boldsymbol{\alpha }}}_{{\boldsymbol{c}}}).$ ${\boldsymbol{\alpha }}$ is a step function whose value is unity when the mesh cell is full of the one phase, and zero if the mesh cell is full of the other phase. The cells containing an interface of the two phases have ${\boldsymbol{\alpha }}$ with values between 0 and 1. The conservation of mass, momentum and energy are the governing equations and formulated mathematically as equations (6–8).

6 $$\frac{\partial \rho }{\partial {\rm{t}}}+\nabla .(\rho {\bf{U}})=0$$

7 $$\frac{\partial (\rho {\bf{U}})}{\partial {\rm{t}}}+\nabla .(\rho {\bf{U}}{\bf{U}})=-\nabla p+\nabla .({\rm{\mu }}(\nabla {\bf{U}}+{\nabla }^{T}{\bf{U}}))+{{\rm{\rho }}f}_{{\rm{\sigma }}}$$

8 $$\frac{\partial (\rho {c}_{p}{\rm{T}})}{\partial {\rm{t}}}+\nabla .(\rho {c}_{p}{\bf{U}}{\rm{T}})=\nabla .(k\,\nabla {\rm{T}})$$

where *p* is pressure, and ${{\rm{f}}}_{{\rm{\sigma }}}$ is force resulted from the surface tension at the interface, and $k$ and ${c}_{p}$ are thermal conductivity and specific heat capacity, respectively. The interfacial force in equation (7) is assessed per unit volume by the continuum surface stress (CSS) method^51,52^ and is given in equation (9).

9 $${{\rm{f}}}_{{\rm{\sigma }}}=\nabla .[{\rm{\sigma }}[|\nabla {\rm{\alpha }}|{\bf{I}}-\frac{\nabla {\rm{\alpha }}\otimes \nabla {\rm{\alpha }}}{|\nabla {\rm{\alpha }}|}]]$$

Where ${\bf{I}}\,\,$is unit tensor and σ is surface tension. The two immiscible fluids are reflected as one effective fluid within the whole domain. The physical properties of the fluids are determined as weighted averages according to the distribution of liquid volume fraction (equations 10 and 11).

10 $${\rm{\rho }}={{\rm{\rho }}}_{{\rm{c}}}\alpha +(1-\alpha ){{\rm{\rho }}}_{{\rm{d}}}$$

11 $$\mu ={\mu }_{{\rm{c}}}\alpha +(1-\alpha ){\mu }_{{\rm{d}}}$$

Where ${{\rm{\rho }}}_{{\rm{d}}}\,\,$and ${{\rm{\rho }}}_{{\rm{c}}}$ are densities of dispersed and continuous phases, respectively. ${\mu }_{{\rm{c}}}$ and ${\mu }_{{\rm{d}}}$ are viscosities of the continuous and dispersed phase, respectively. Since the immiscible fluids cannot be blended, the following three criteria should be met^53^; (i) The local normal component of the velocities for each fluid needs to be equal to the interface velocity; (ii) The velocities tangent to the interface inside and outside the droplet need to be equal; and (iii) The tangential shear stresses declared by equation (12) need to be balanced at the interface.

12 $${\mu }_{d}{\frac{\partial {{\rm{u}}}_{t}}{\partial {\rm{r}}}|}_{d}={\mu }_{c}{\frac{\partial {{\rm{u}}}_{t}}{\partial {\rm{r}}}|}_{c}$$

where ${\frac{\partial {{\rm{u}}}_{t}}{\partial {\rm{r}}}|}_{d}\,{\rm{and}}\,{\frac{\partial {{\rm{u}}}_{t}}{\partial {\rm{r}}}|}_{c}$ are derivatives of the tangential velocity with respect to *r*-direction inside and outside of the droplet, respectively. 2D simulations suffice for the present geometry to predict the behaviour of droplets when heat is added. All flow simulations were performed using ANSYS-Fluent software. The module multiphase with activating Energy equation, the transient condition was used to solve the equations numerically. The physical properties of the fluids utilized in this study are temperature dependent Table 2 ^20^.

**Table 2:** Physical properties of the continuous and dispersed phases used in numerical simulations^20^ (Temperature in Kelvin, T_ref_ = 298 K).

Properties	water	Mineral oil
Density [kg m^−3^]	−0.204 T + 1005.2	−0.524 T + 1002.4
Viscosity [Pa s]	1E-7 T^2^ − 8E-5 T + 0.0155	9E-06 T^2^ − 0.0061 T + 1.0478
Thermal conductivity [W m^−1^ K^−1^]	0.6	0.14
Specific heat [J kg^−1^ K^−1^]	4182	2500
Surface tension [N m^−1^]	−0.0001 T + 0.048	

### Controlling non-dimensional parameters

The key parameters controlling the performance of droplets microgenerator include the physical properties and flow rates of the dispersed and continuous phases, and the dimensions of microchannels^54^. These parameters are compiled by two dimensionless groups, flow rate ratio (${\boldsymbol{\phi }}$) and capillary number (${\boldsymbol{Ca}}$). ${\boldsymbol{\phi }}$ between the two immiscible fluids largely dominates the droplet size.

13 $$\phi =\frac{{Q}_{d}}{{Q}_{c}\,}$$

The capillary number ($$Ca)$$ describes the contest between the capillary pressure (resisting the deformation of liquid interface) and viscous shear stresses (that causes the deformation of the liquid interface). In flow-focusing configurations, $$Ca$$ is defined as equation (14)^55,56^.

14 $$Ca=\frac{{\mu }_{c}G{a}_{o}}{{\rm{\sigma }}}$$

where $G$ is the characteristic deformation rate $G=\frac{{Q}_{c}}{h\Delta z\,}(\frac{1}{{w}_{o}}-\frac{1}{2{w}_{c}})$, ${a}_{o}\,$is the characteristic droplet radius ${a}_{o}=\frac{{w}_{d}}{2}$, and $h$ is the depth of channel, the s. $\Delta z$ is axial distance between the end of the inlet channels and the orifice with width ${w}_{o}$. The subscripts *c* and *d* represent the continuous and dispersed phases, respectively. *µ* is viscosity, ρ is density, ${\rm{\sigma }}$ is interfacial tension, *Q* is volumetric flow rate, and $w$ is width of the channel. The capillary number ($Ca$) at room temperature is approximately equal to 0.01 for all scenarios tested in this work. The measured droplet diameter ($D$) and temperature ($T$) are also cast in a dimensionless form. The width of the downstream channel is taken as the characteristic length. The droplets have spherical shape when $D$ is less than the depth of the microchannel ($h$); otherwise they would have discoid shape where the equivalent a diameter (${D}_{eq}$) is obtained from the correlation proposed by Nie *et al*.^57^.

15 $${D}_{eq}=\{\begin{array}{l}D,\,D < h\\ \sqrt[3]{\frac{[2{D}^{3}-{(D-h)}^{2}(2D+h)]}{2}},D > h\end{array}\,$$

The normalized drops diameter is defined as ${D}^{\ast }=\frac{{D}_{eq}}{{w}_{out}}$ where ${w}_{out}$ is width of the downstream channel. The dimensionless temperature ($\theta$) is defined as $\frac{T-\,{T}_{R}}{{T}_{m}-\,{T}_{R}}$, where ${T}_{R}$ is room temperature and ${T}_{m}$ is the highest temperature investigated in this work. Therefore, $\theta$ would have values between 0 to 1.

## Supplementary information

Movie 1

Movie 2

Supplementary Information
