# Supplementary material for: Dynamics of temperature-actuated droplets within microfluidics
Source: Sci Rep. 2019 Mar 7;9:3832. doi: 10.1038/s41598-019-40069-9 (PMC6405956; doi:10.1038/s41598-019-40069-9)
Supplement: Supplementary file 3 — Supplementary Information [file 41598_2019_40069_MOESM3_ESM.docx]

**Dynamics of temperature-actuated droplets within microfluidics**

Asmaa Khater^a,b^, Mehdi Mohammadi^a,b^, Abdulmajeed Mohamad ^*a^ and Amir Sanati Nezhad^*a,b,c^

*^a^ Department of Mechanical and Manufacturing Engineering, University of Calgary, Calgary, AB T2N 1N4 Canada*

*^b^ BioMEMS and Bioinspired Microfluidic Laboratory, Department of Mechanical and Manufacturing Engineering, University of Calgary, Calgary, AB T2N 1N4 Canada*

*^c^ Centre for Bioengineering Research and Education, University of Calgary, Calgary, AB T2N 1N4, Canada*

* Corresponding authors

E-mails:

[mohamad@ucalgary.ca](mailto:mohamad@ucalgary.ca)

[amir.sanatinezhad@ucalgary.ca](mailto:amir.sanatinezhad@ucalgary.ca)

**Supplementary Information (SI):**

## S1. Heater calibration

The heater was calibrated to temperature of 95 °C with placing the chip on the heater’s top surface. The voltage of the heater was changed using a DC power supply and temperature change was monitored continuously by an IR camera. Temperature gradient in the direction normal to the heater is shown in **Fig**. S1.

| 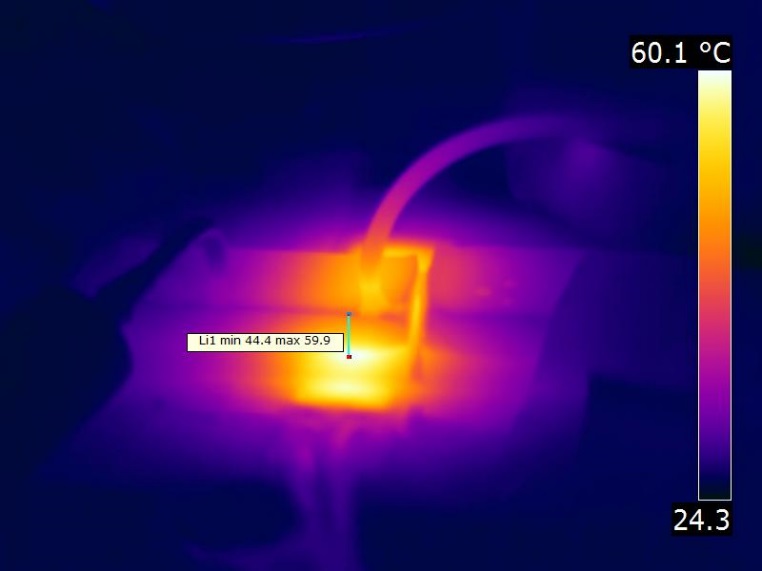  **a)** | 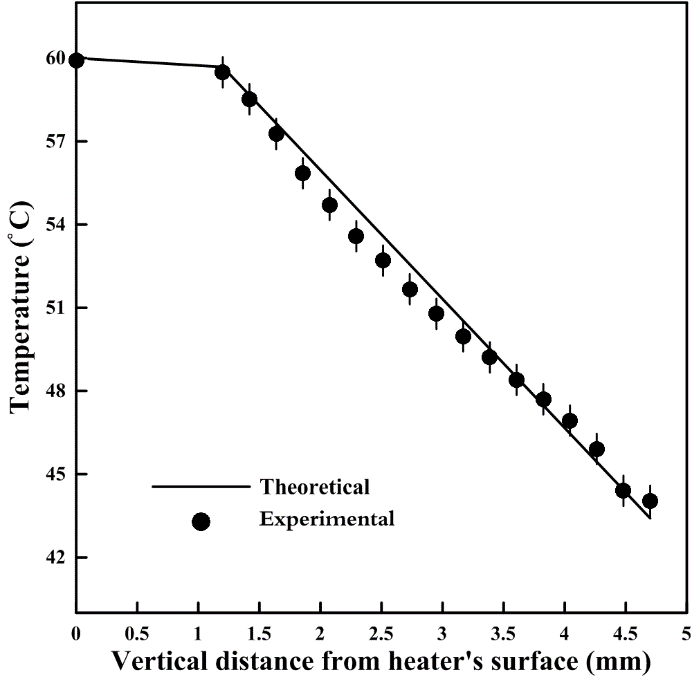  **b)**  **d)** |
| --- | --- |
| 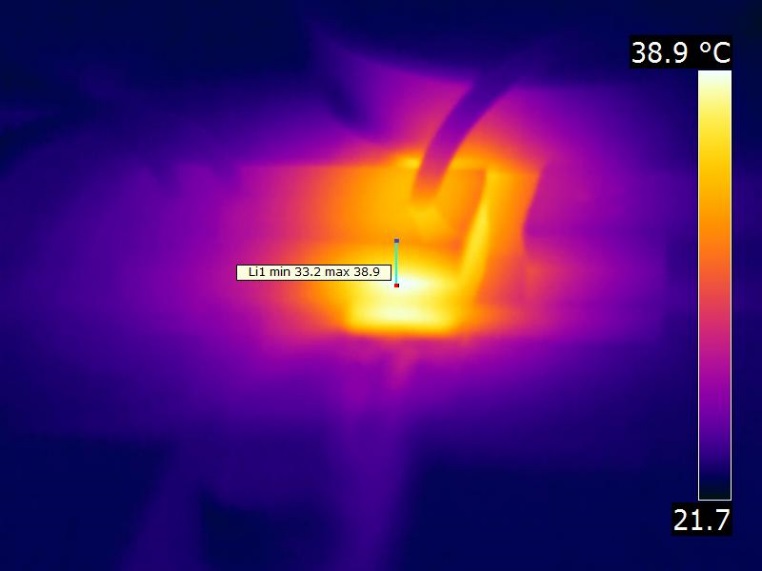  **c)** | 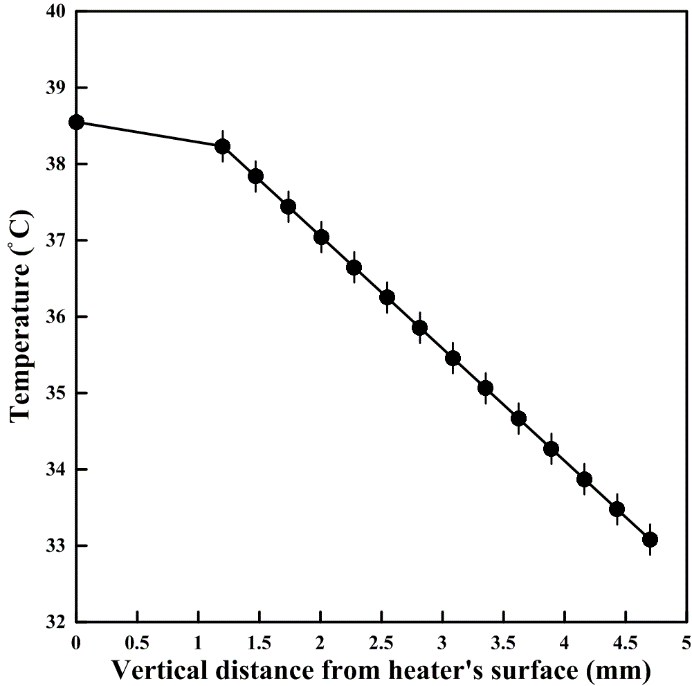 |


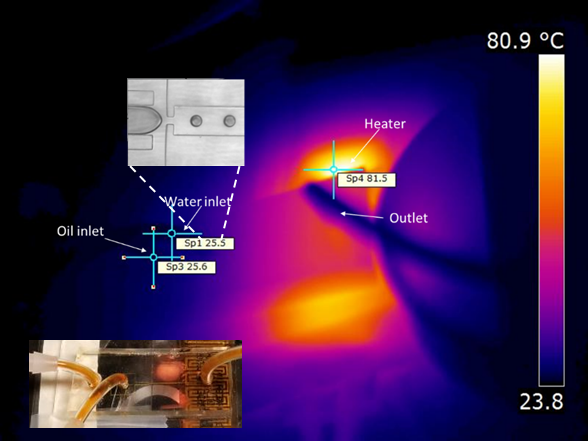


**e)**

**Figure S1** Temperature distribution within the chip placed over the heater and captured by Infrared (IR) camera. **(a)** the three-dimensional (3D) image of the temperature distribution within the chip for the heater set at 60°C, **(b)** the diagram showing the experimental results extracted from IR camera when the heater is set at 60°C and the temperature distribution calculated from Newton's Law of cooling, **(c)** the temperature distribution within the chip placed on the heater is set at 39 °C, **(d)** The diagram showing the experimental results extracted from IR camera when the heater is set at 39 °C and the temperature distribution calculated from Newton's Law of cooling. **(e)** Temperature of inlets and droplet generation site remains below 26 °C even when the heater temperature reaches about 90 ^o^C.

## S2. Experimental results for all tested cases

**Table S1.** The phase diagram of droplet generation at different temperatures and flow rate ratios for pure mineral oil used as the continuous phase.

| Flow rate ratio | 25 °C | 40 °C | 50 °C | 60 °C | 70 °C | 80 °C | 90 °C |
| --- | --- | --- | --- | --- | --- | --- | --- |
| 0.10 | 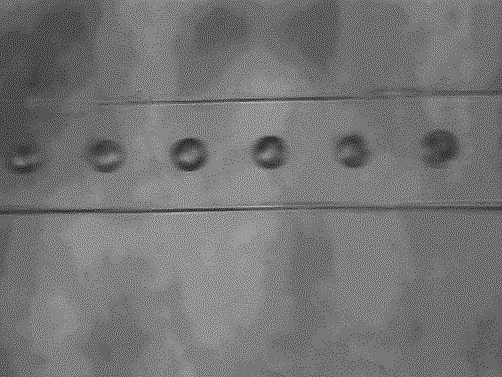 | 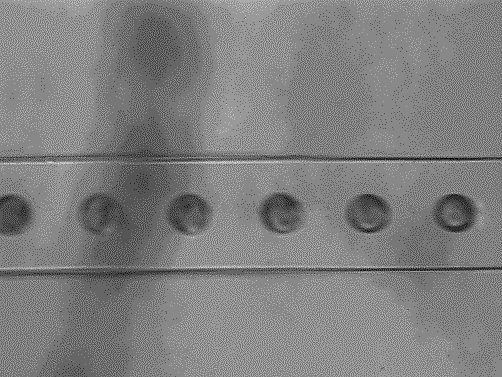 | 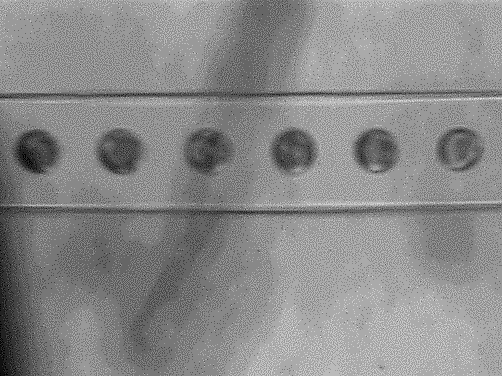 | 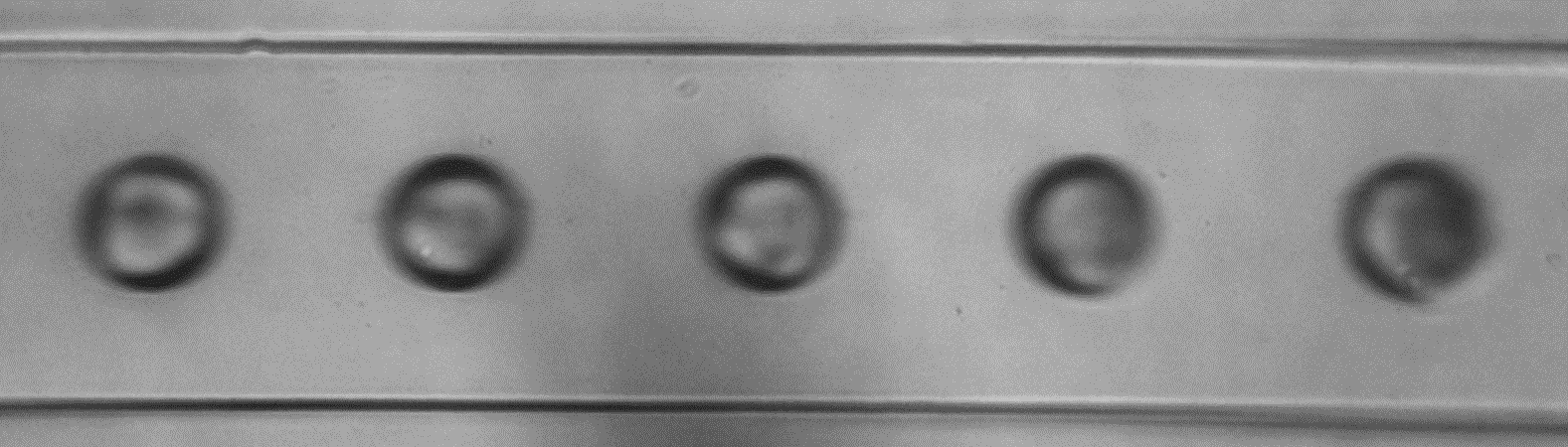 | 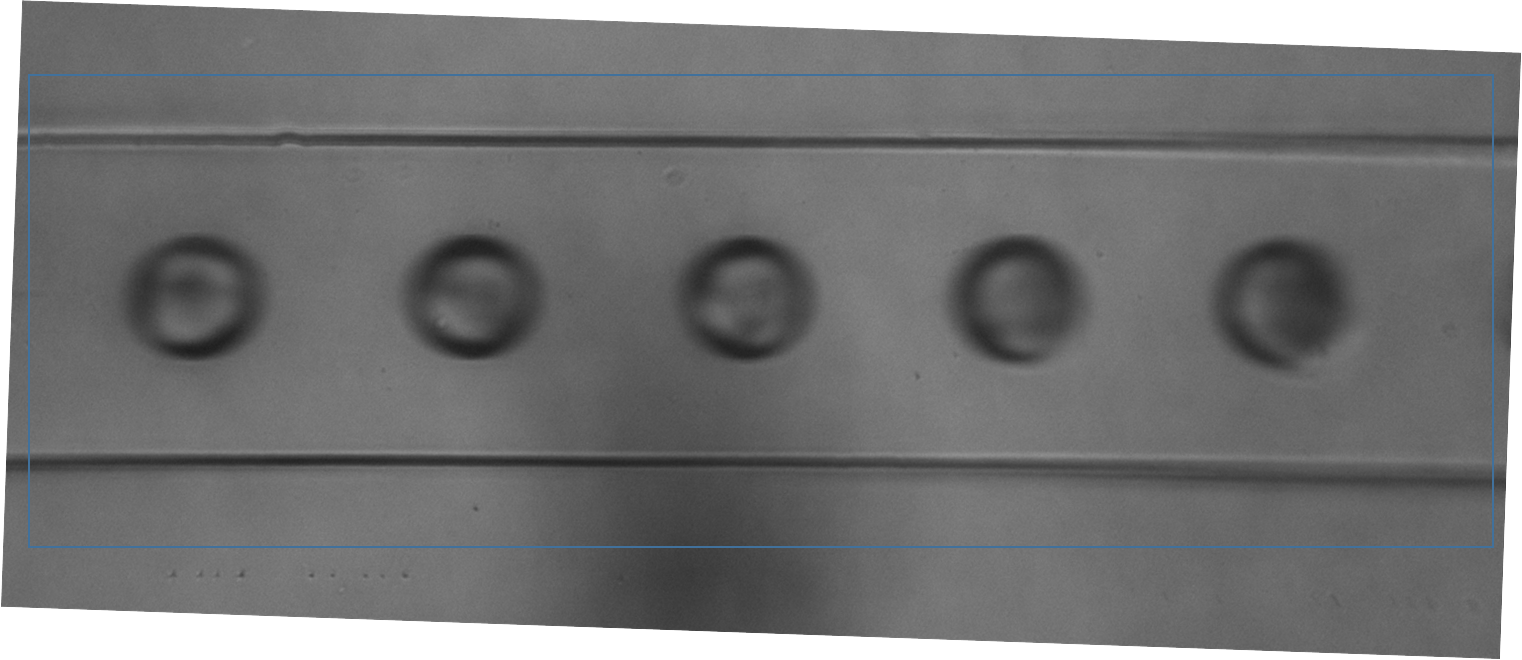 | 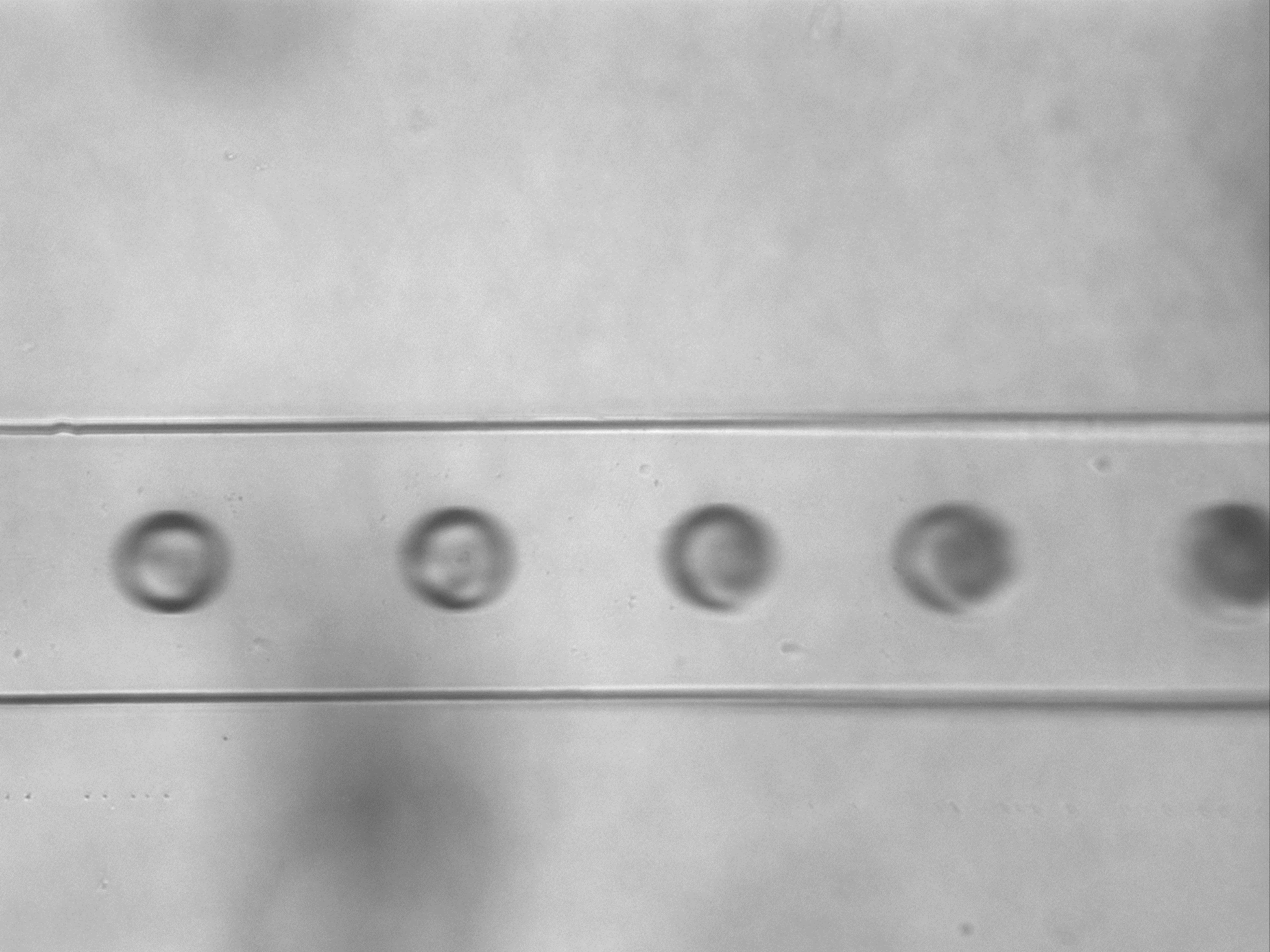 | 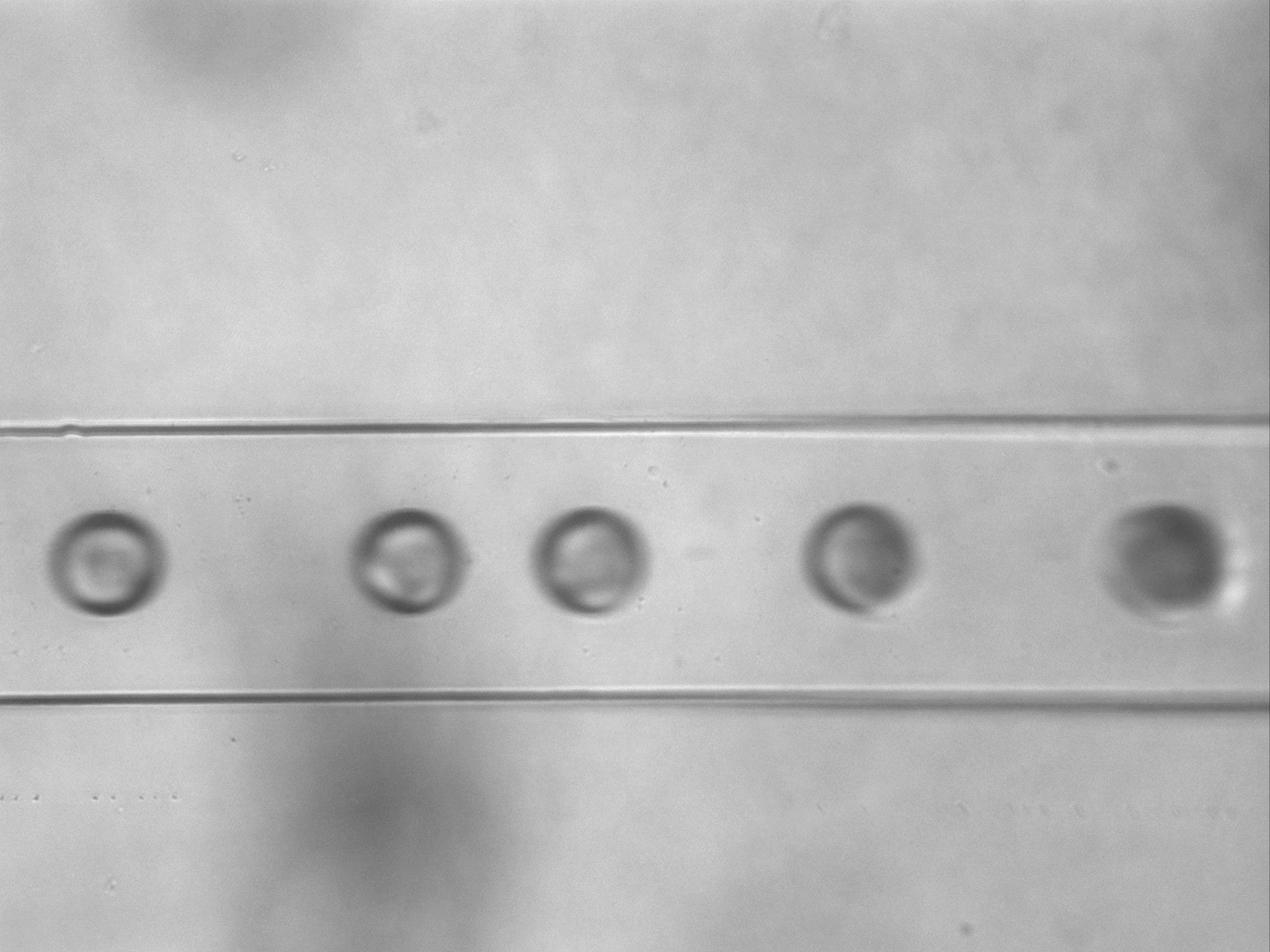 |
| 0.13 | 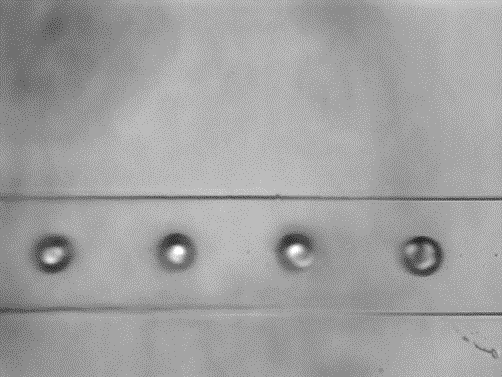 | 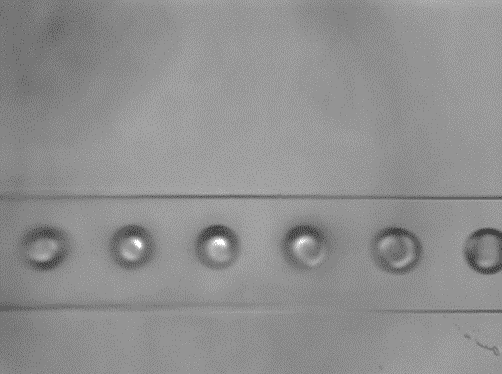 | 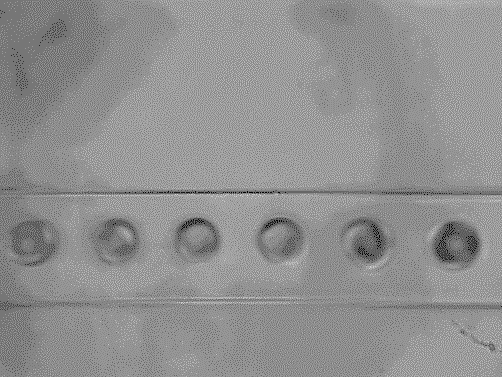 | 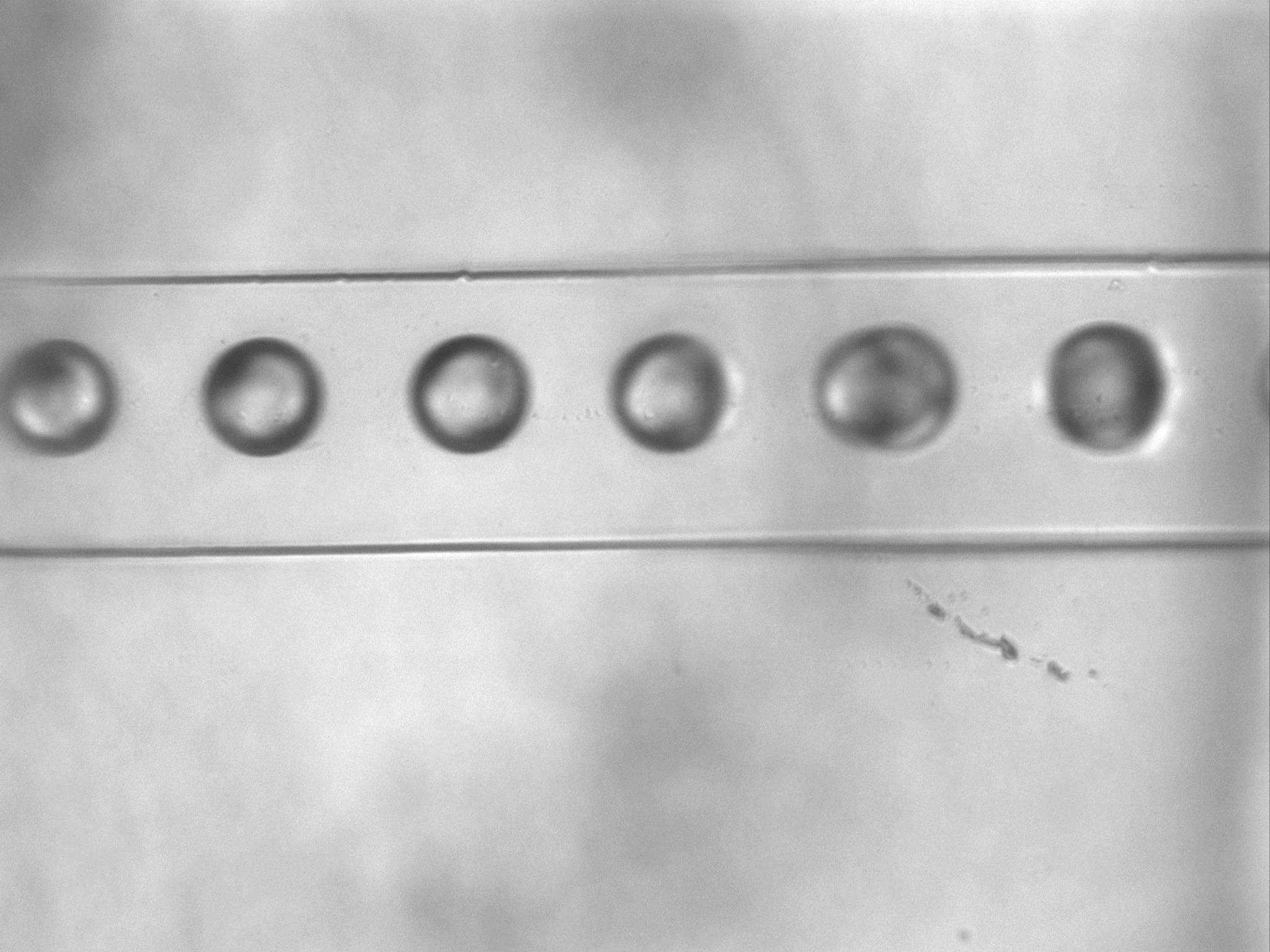 | 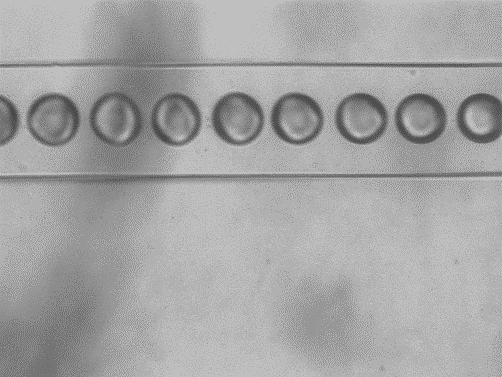 | 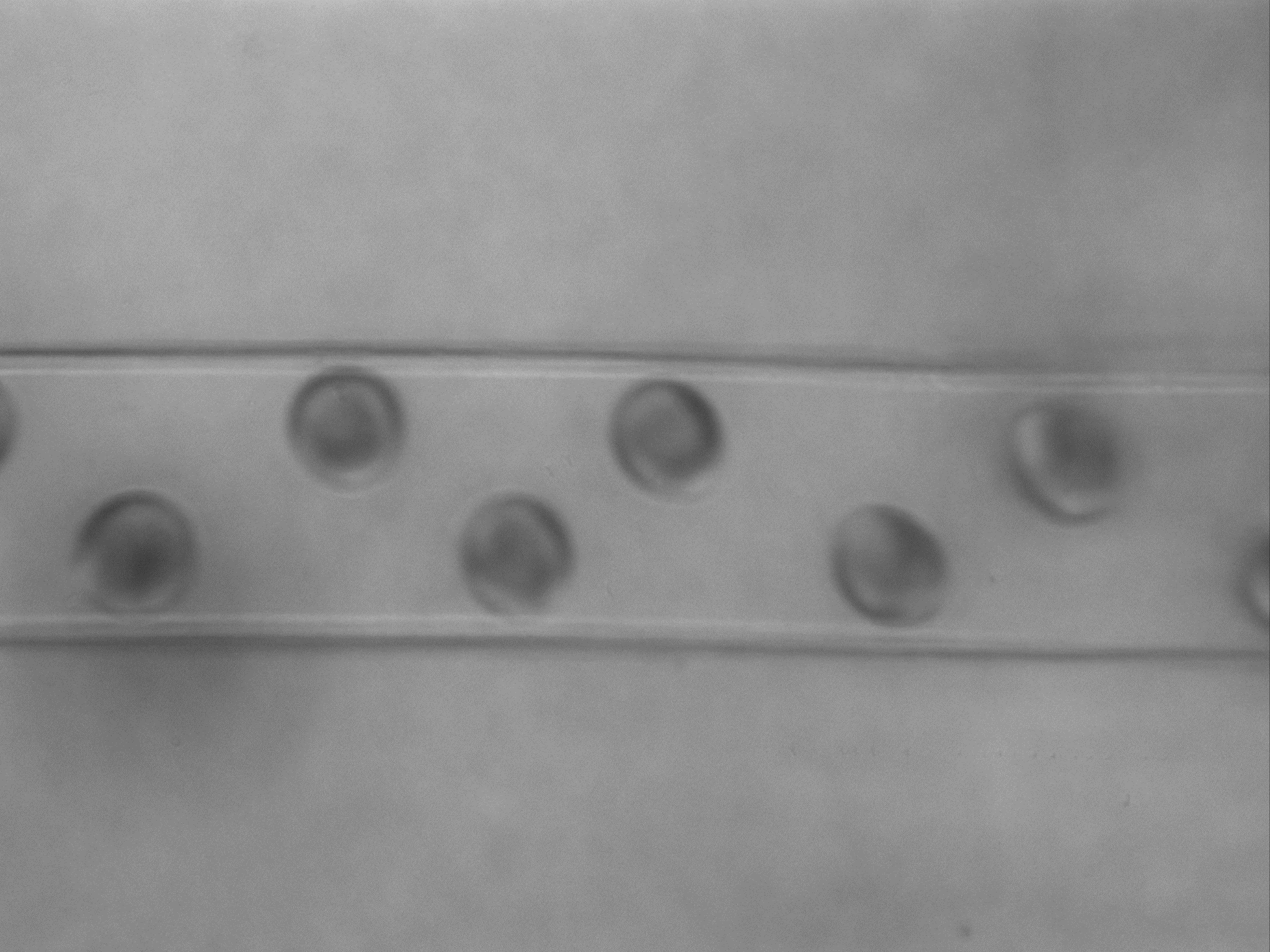 | 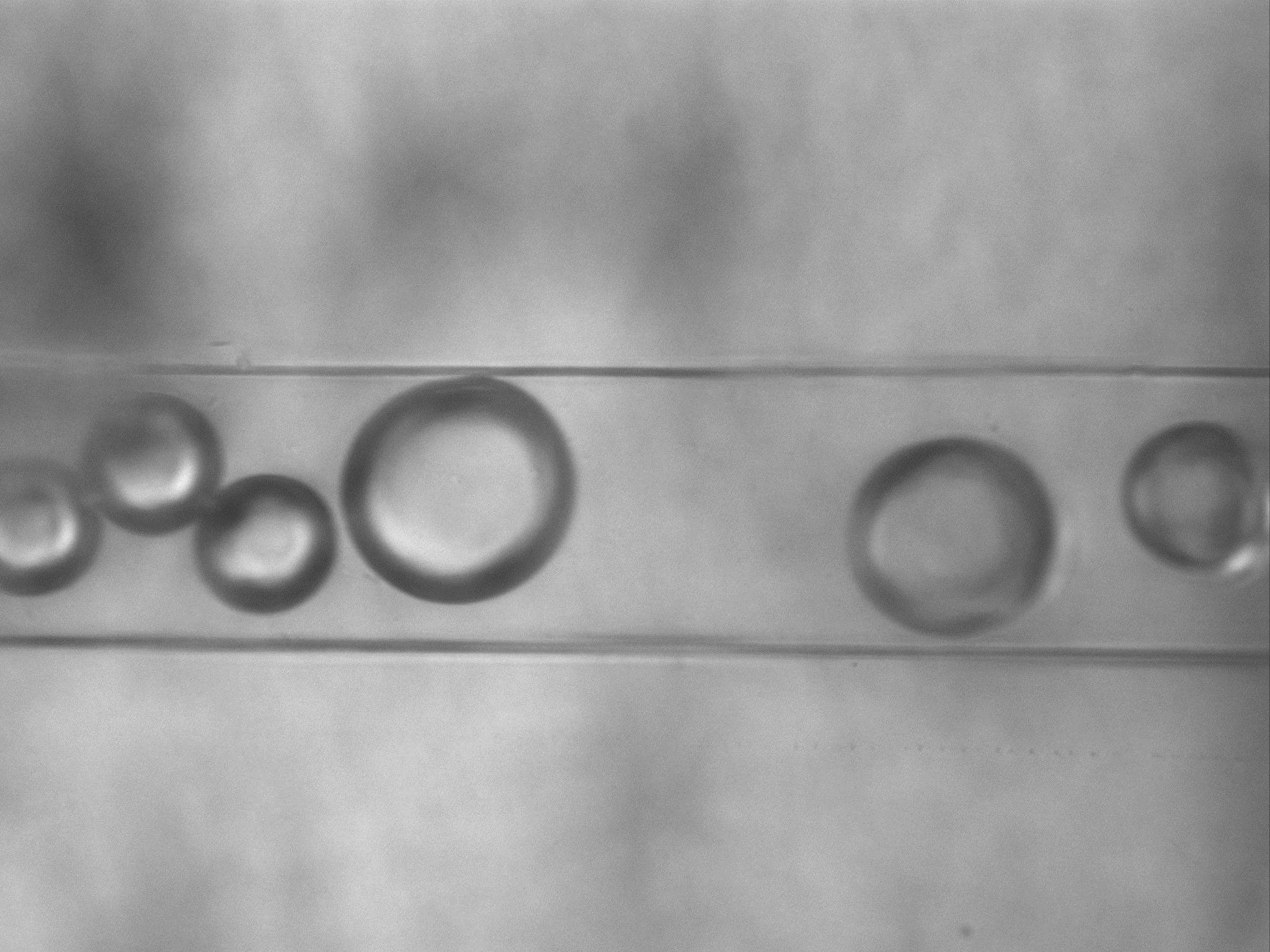 |
| 0.15 | 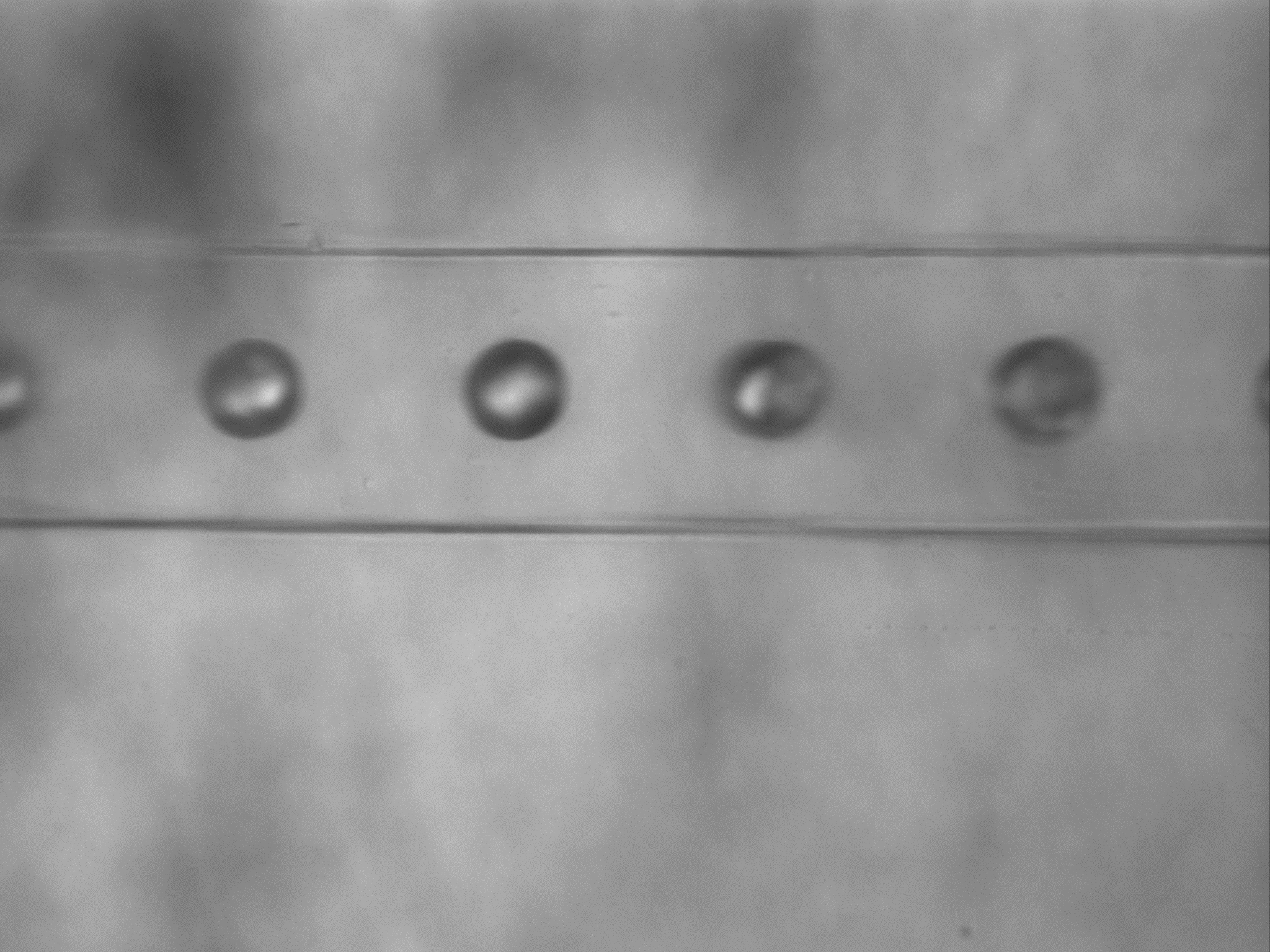 | 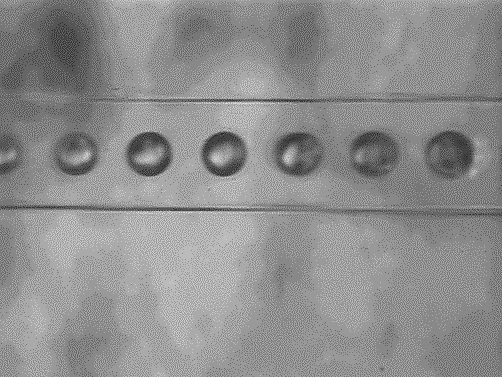 | 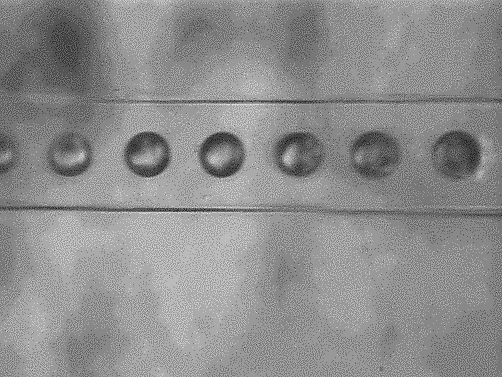 | 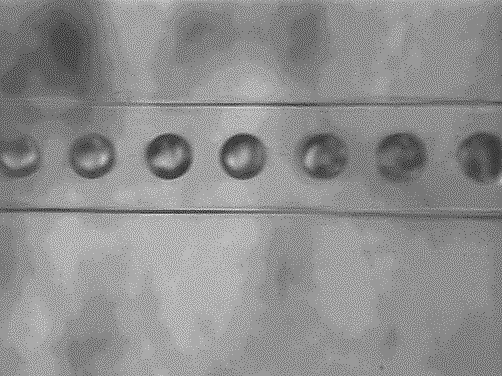 | 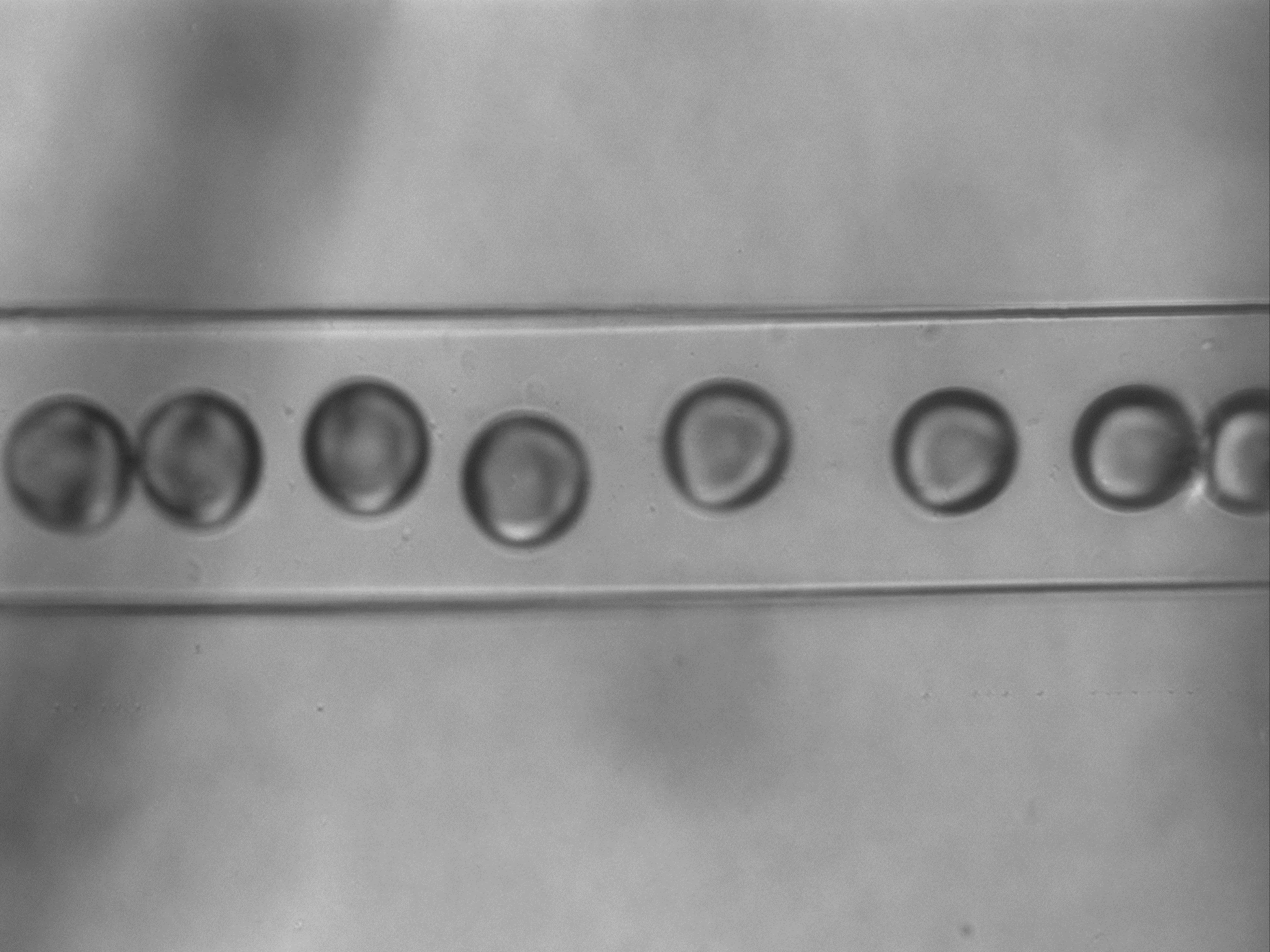 | 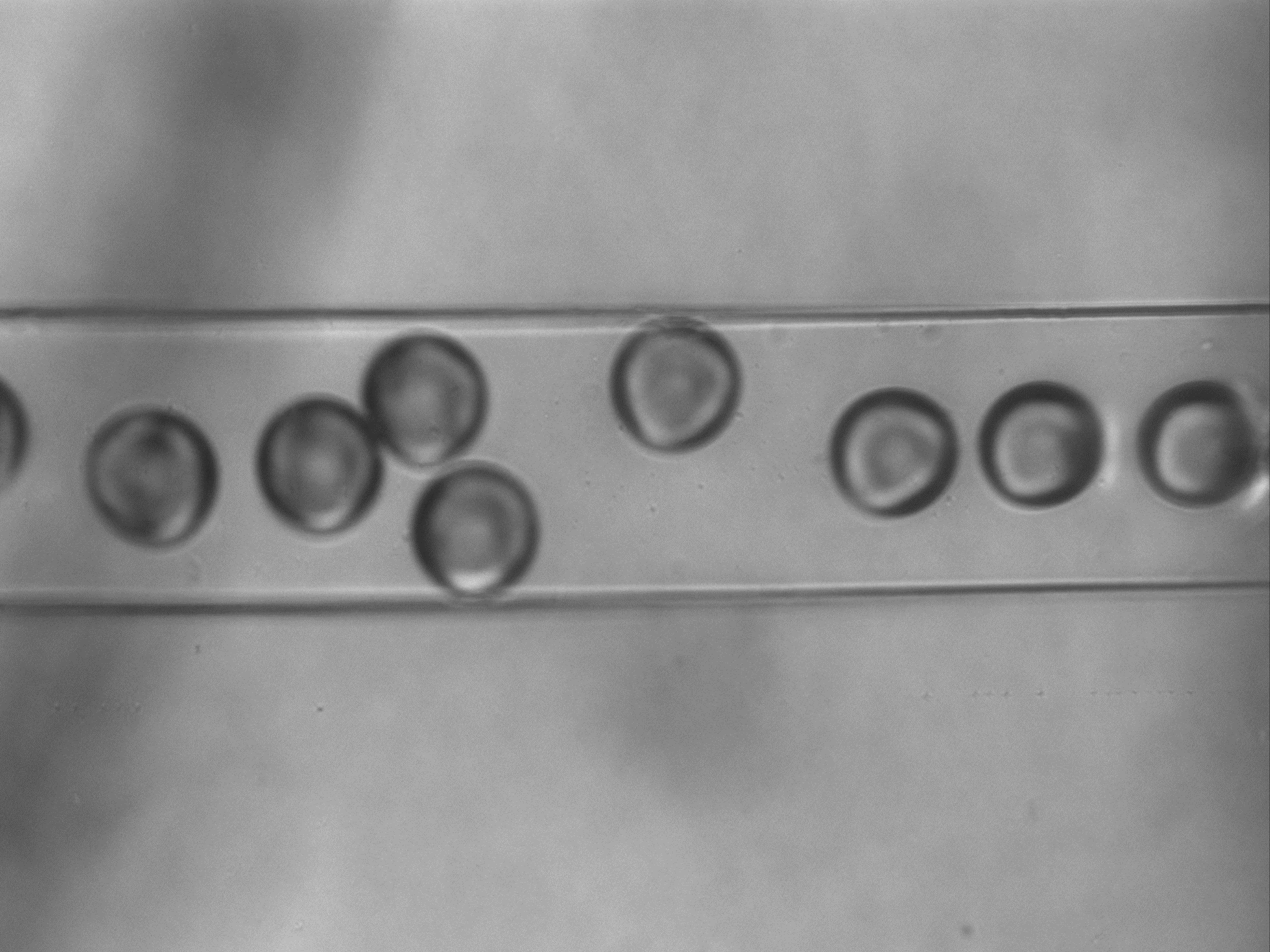 | 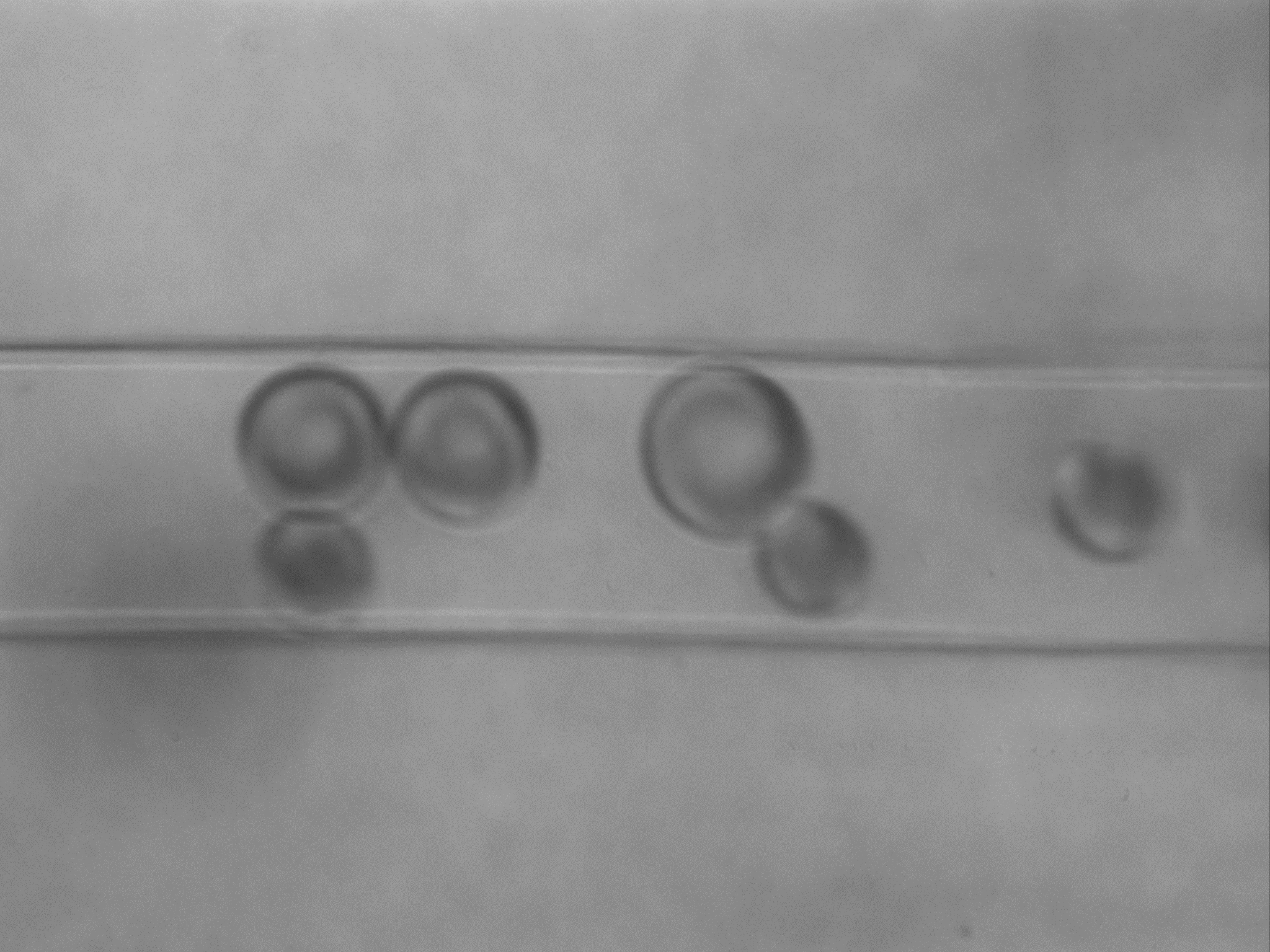 |
| 0.20 | 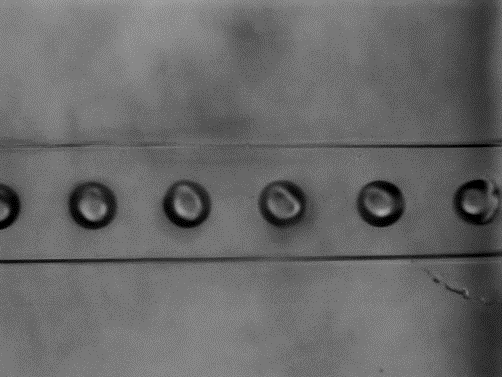 | 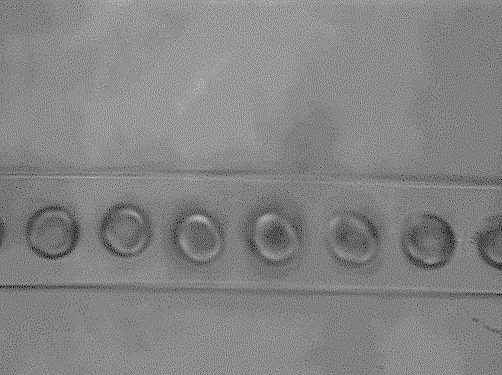 | 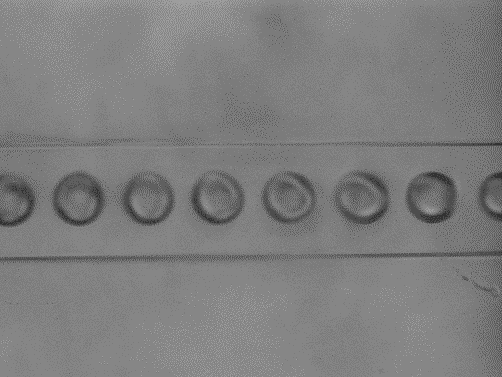 | 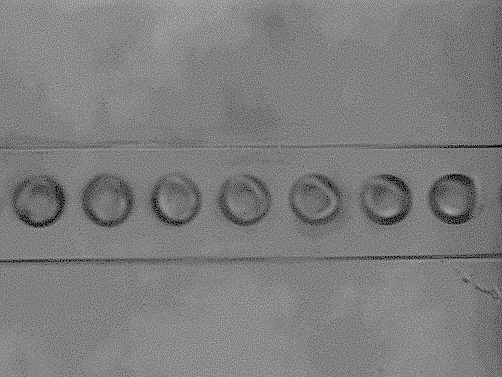 | 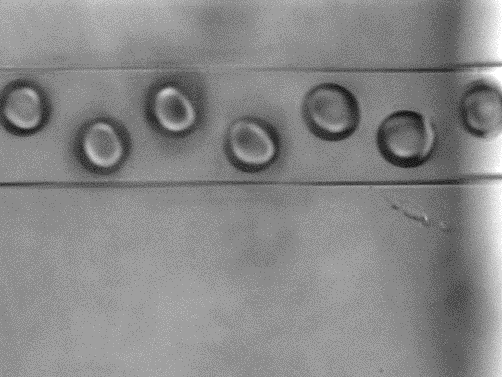 | 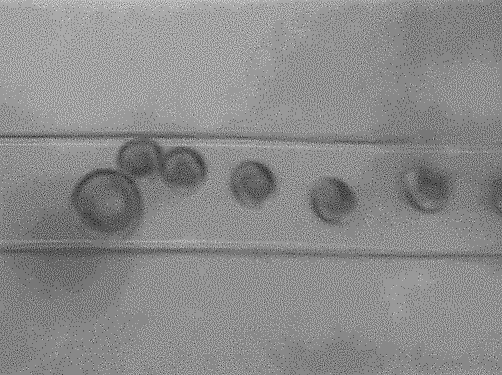 | 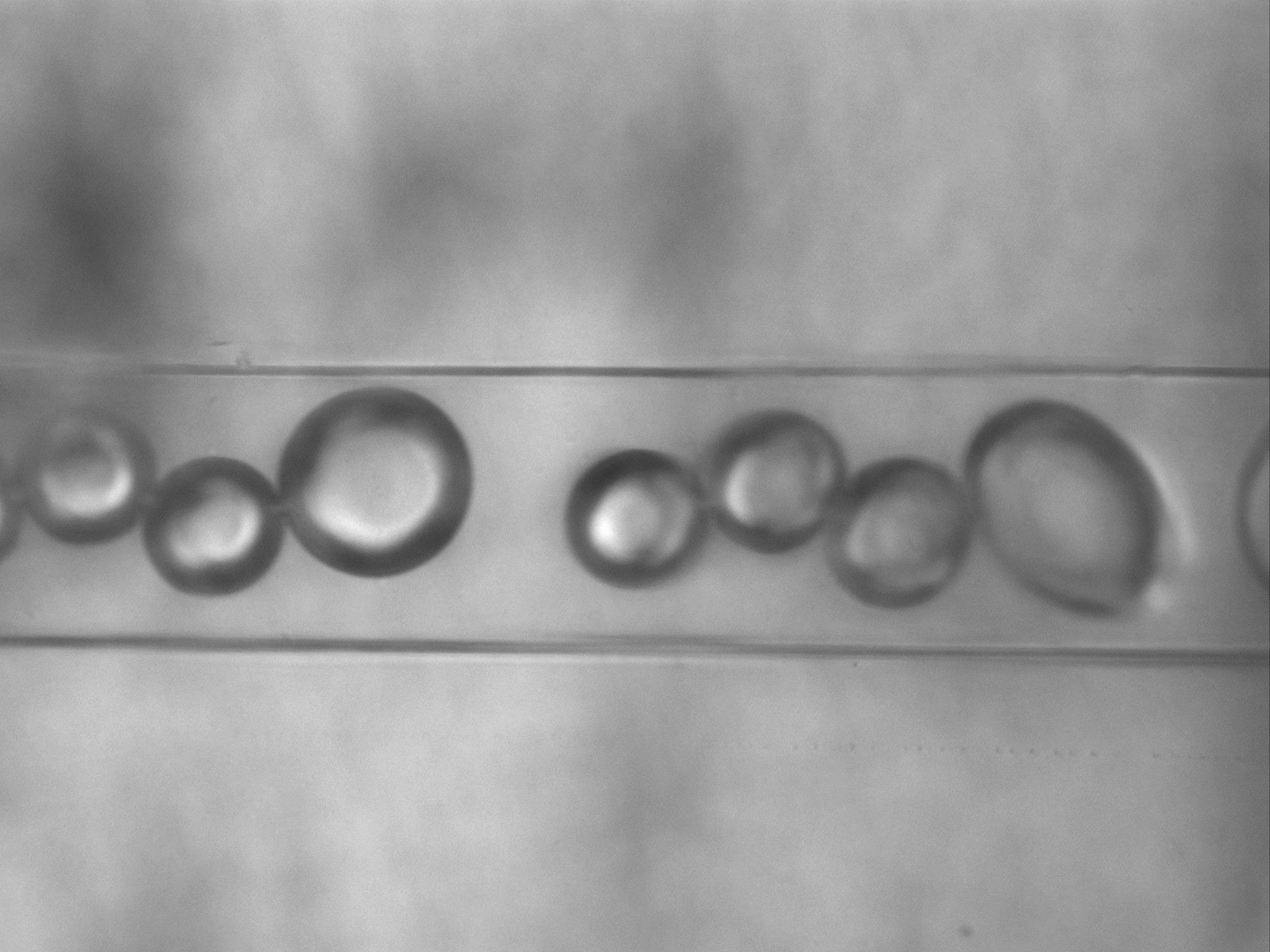 |
| 0.25 | 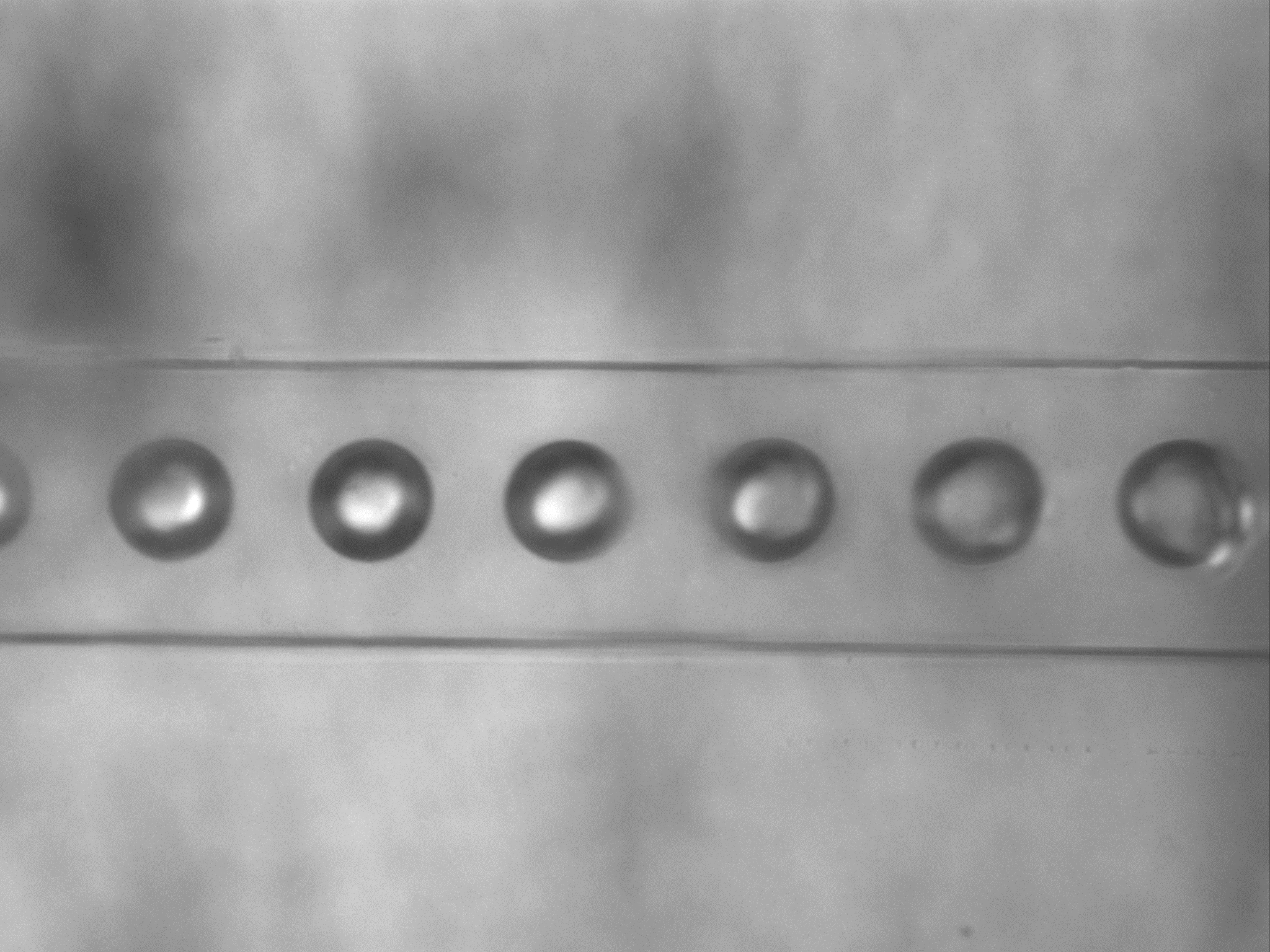 | 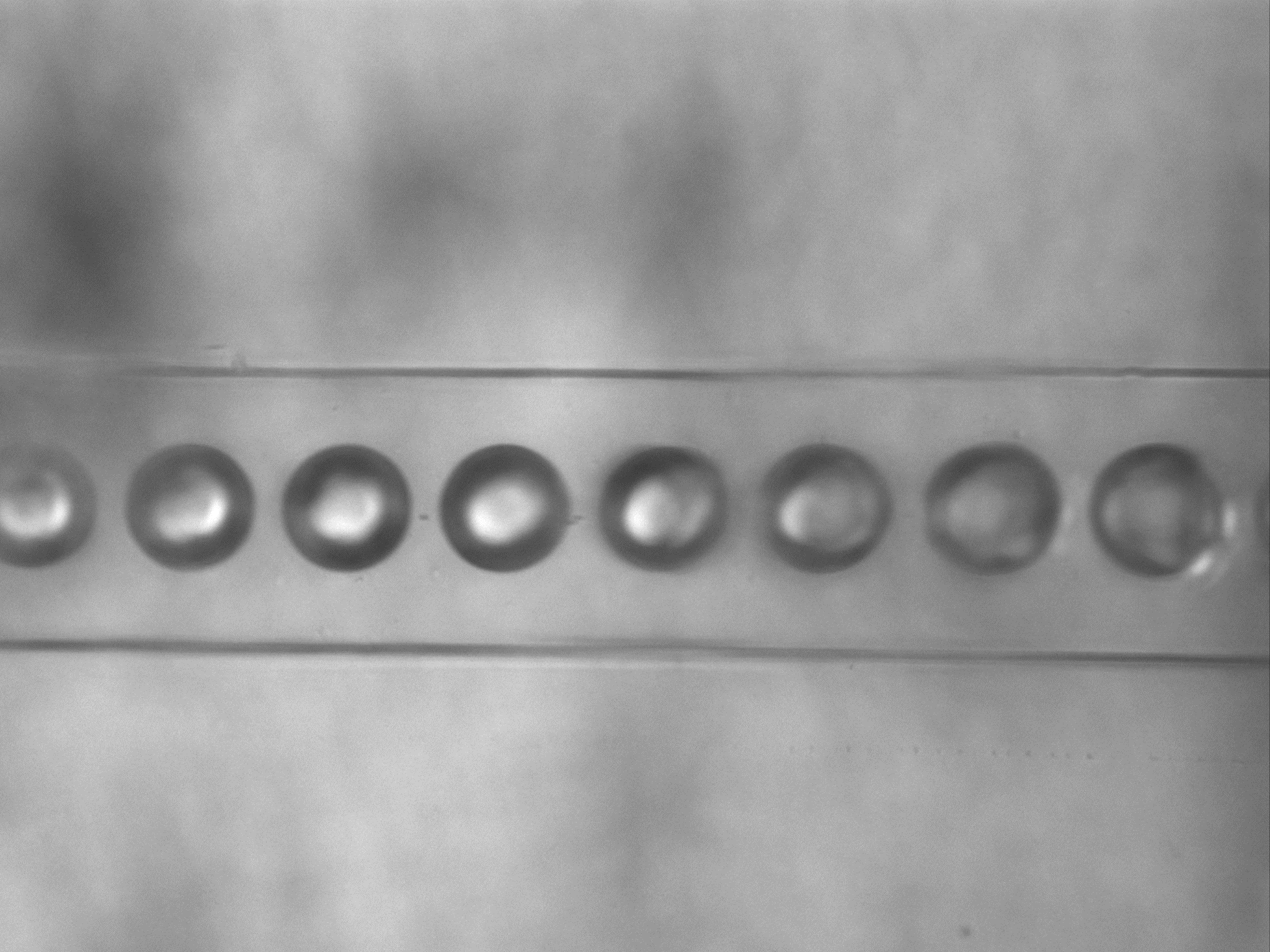 | 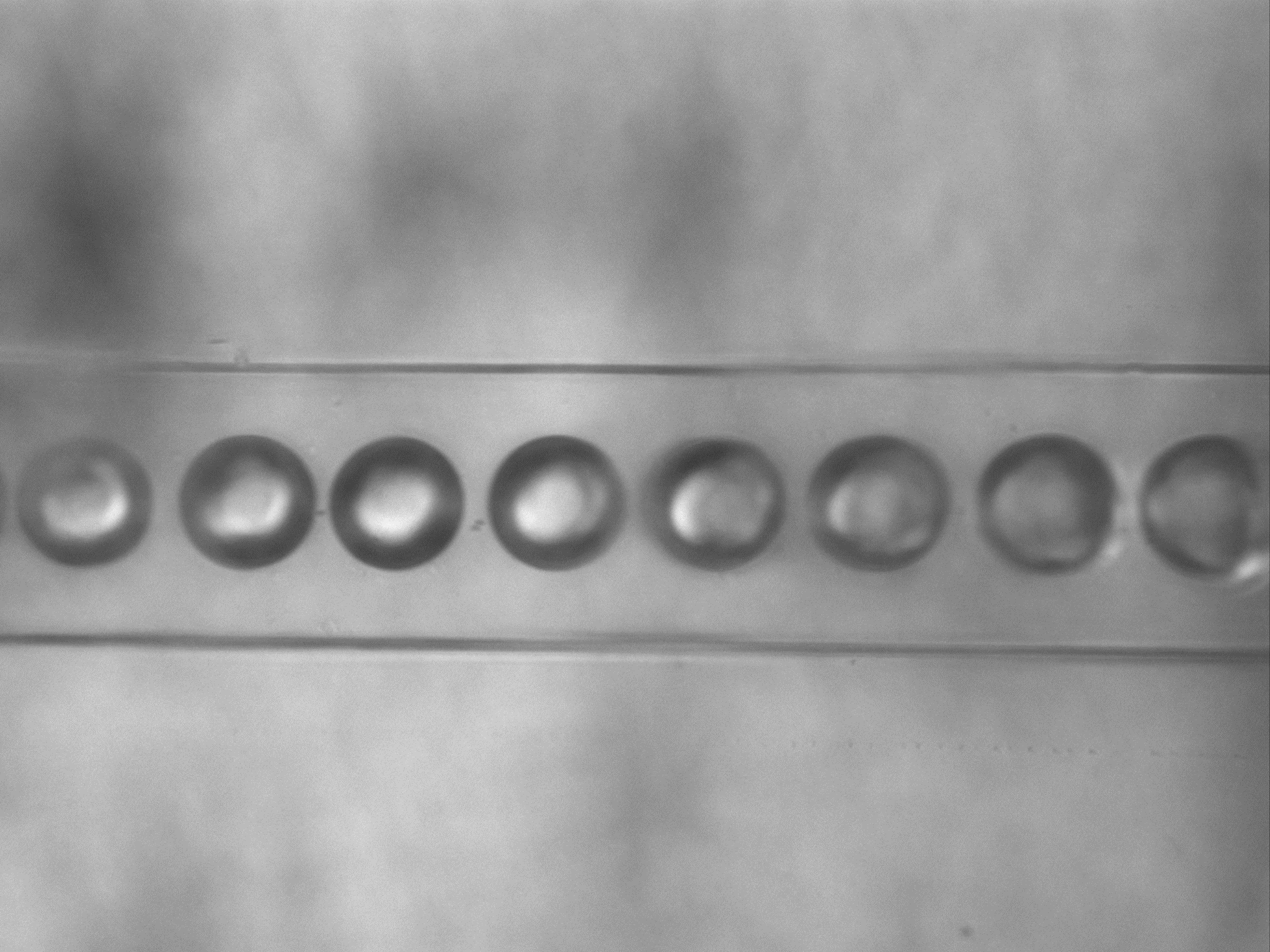 | 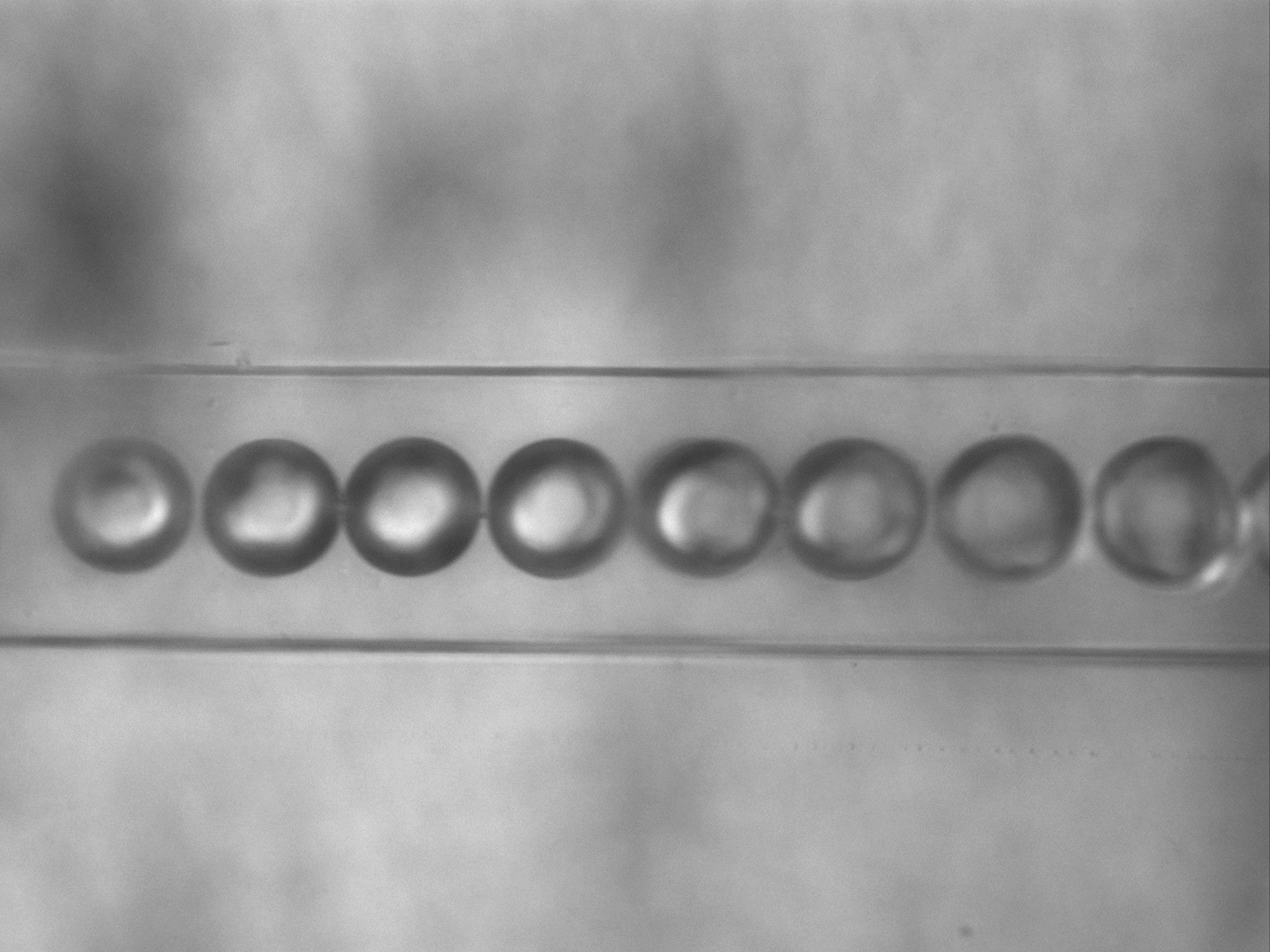 | 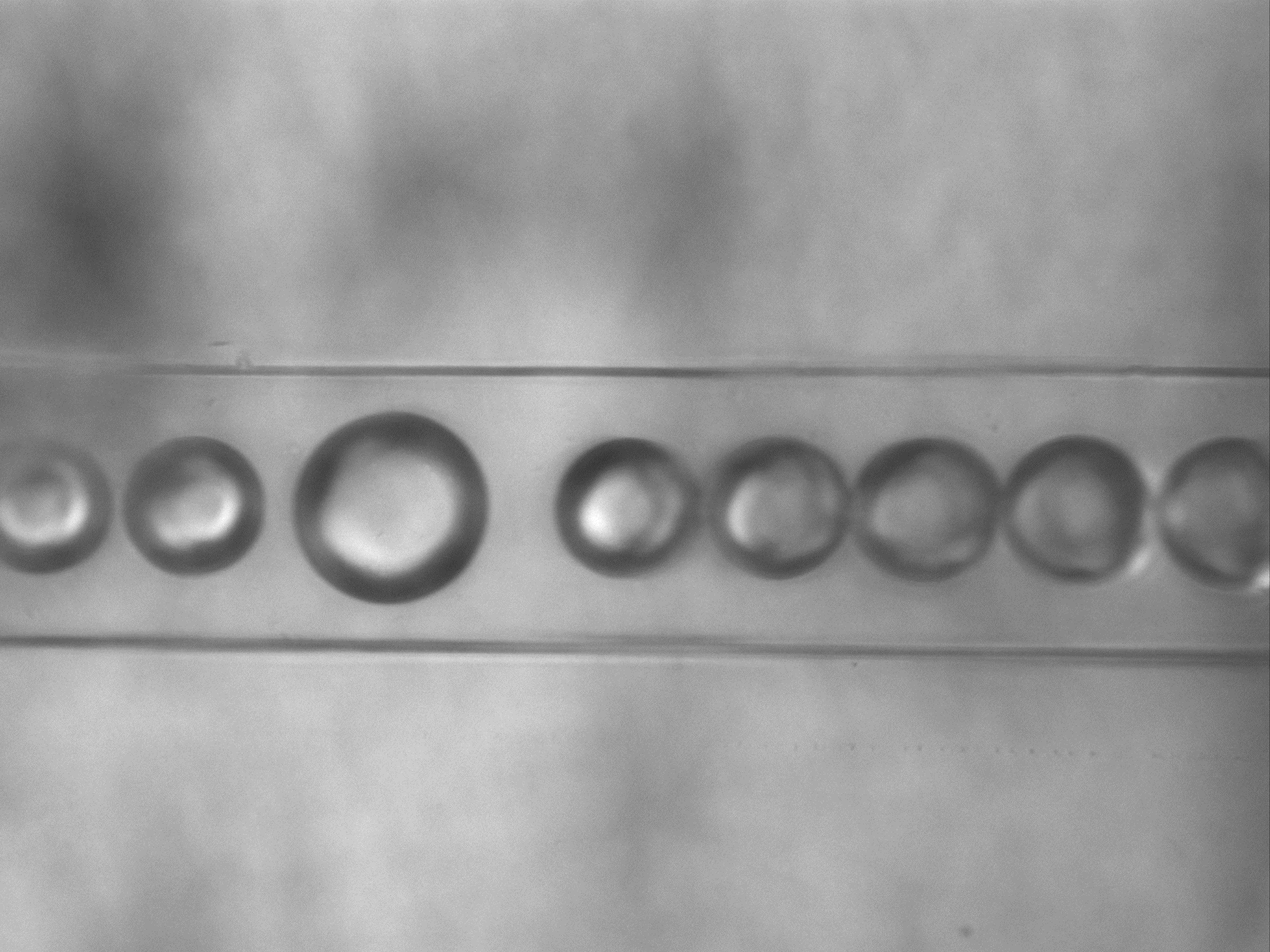 | 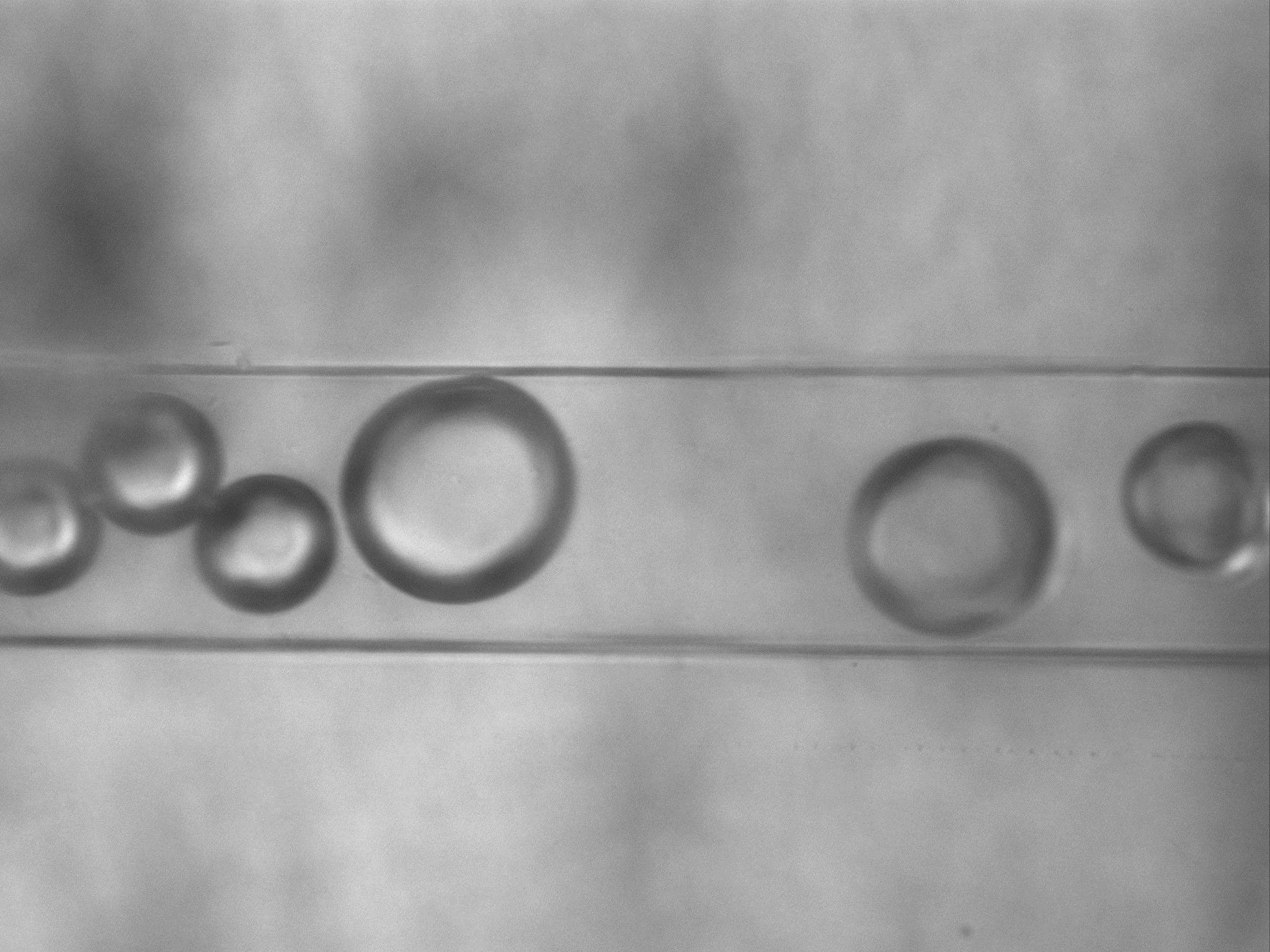 |  |

**Table S2** The phase diagram of droplet generation at different temperatures and flow rate ratios for mineral oil with surfactant used as the continuous phase.

| Flow rate ratio | 25 °C | 40 °C | 50 °C | 60 °C | 70 °C | 80 °C | 90 °C |
| --- | --- | --- | --- | --- | --- | --- | --- |
| 0.10 | 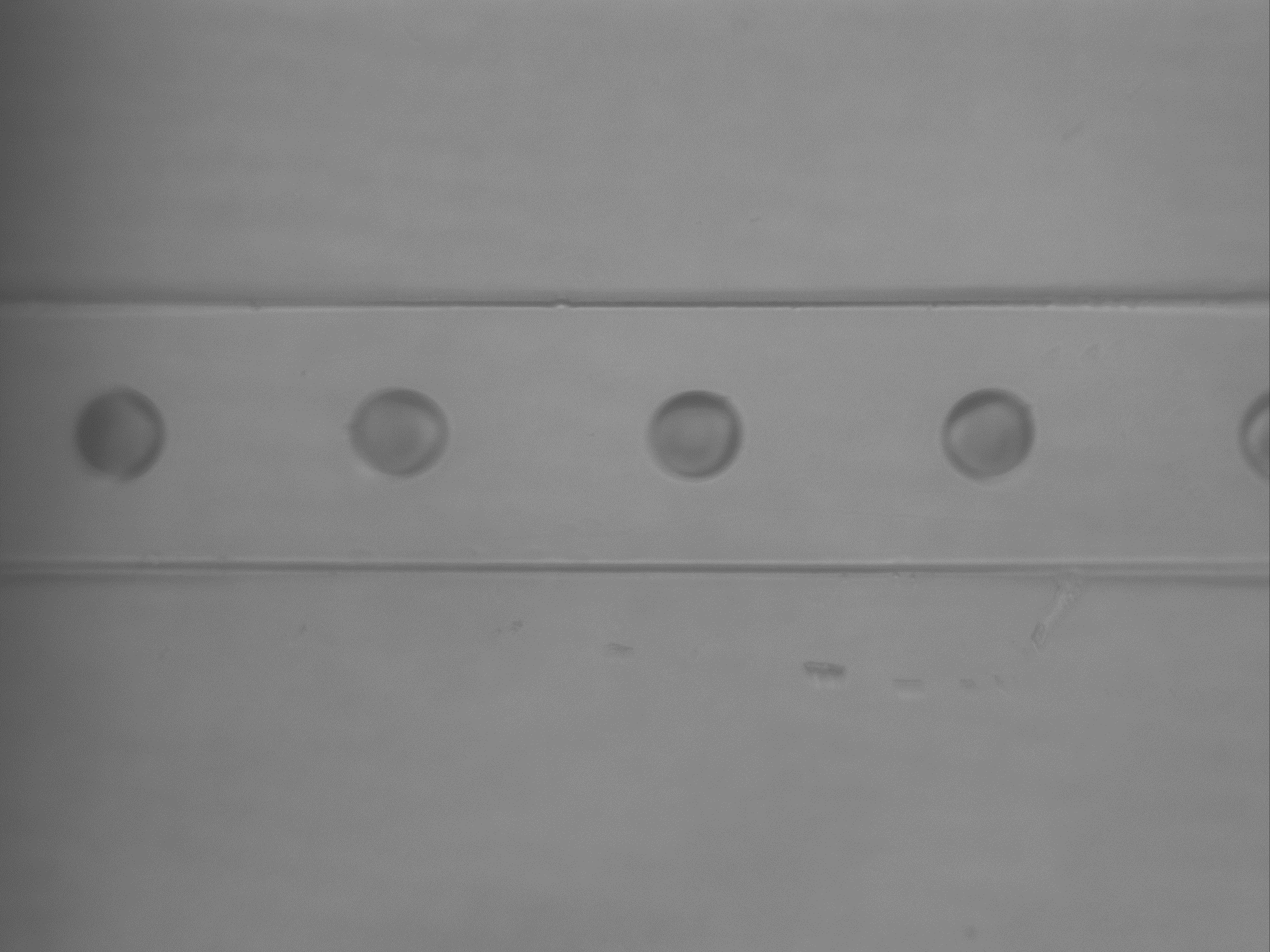 | 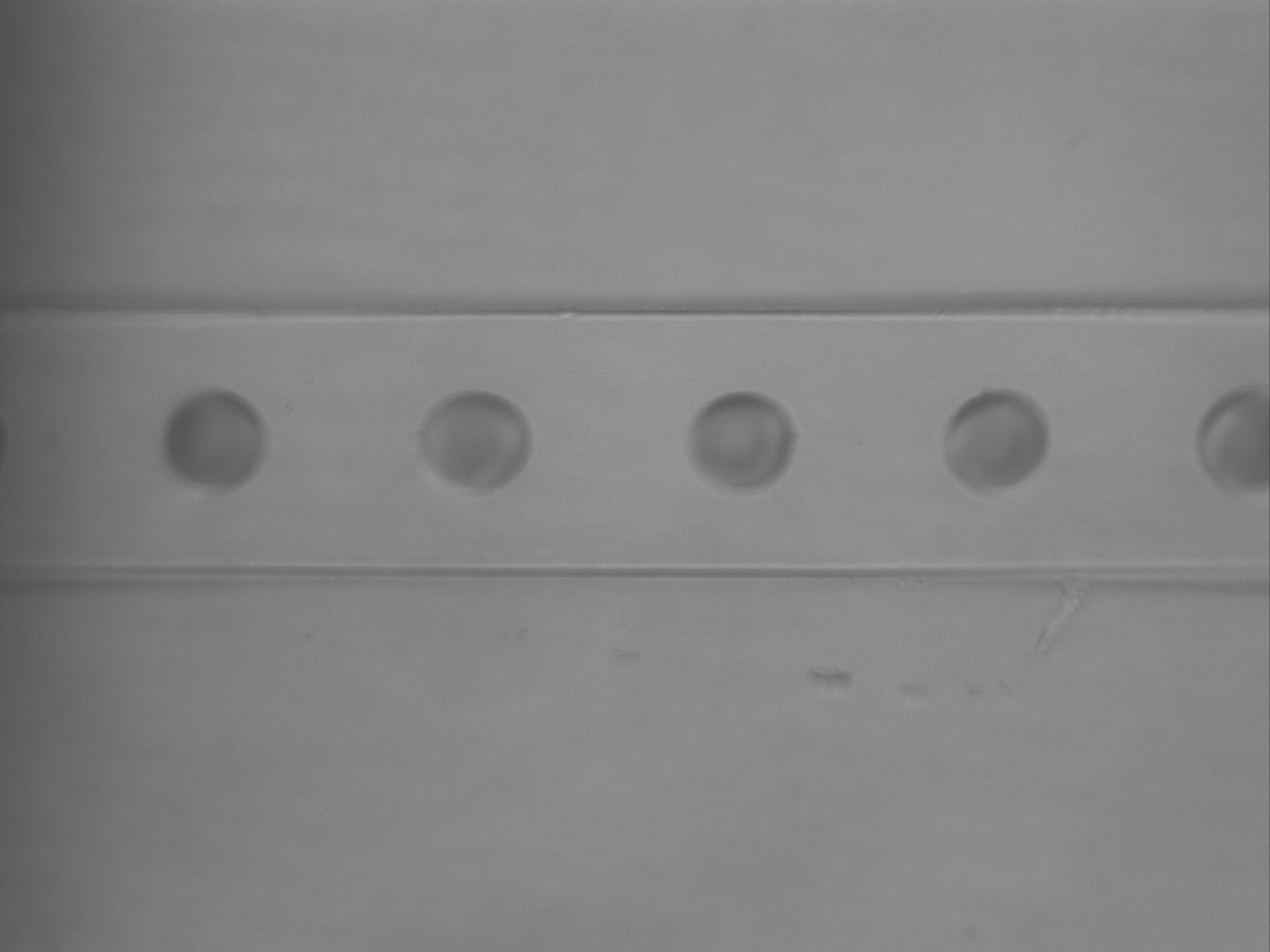 | 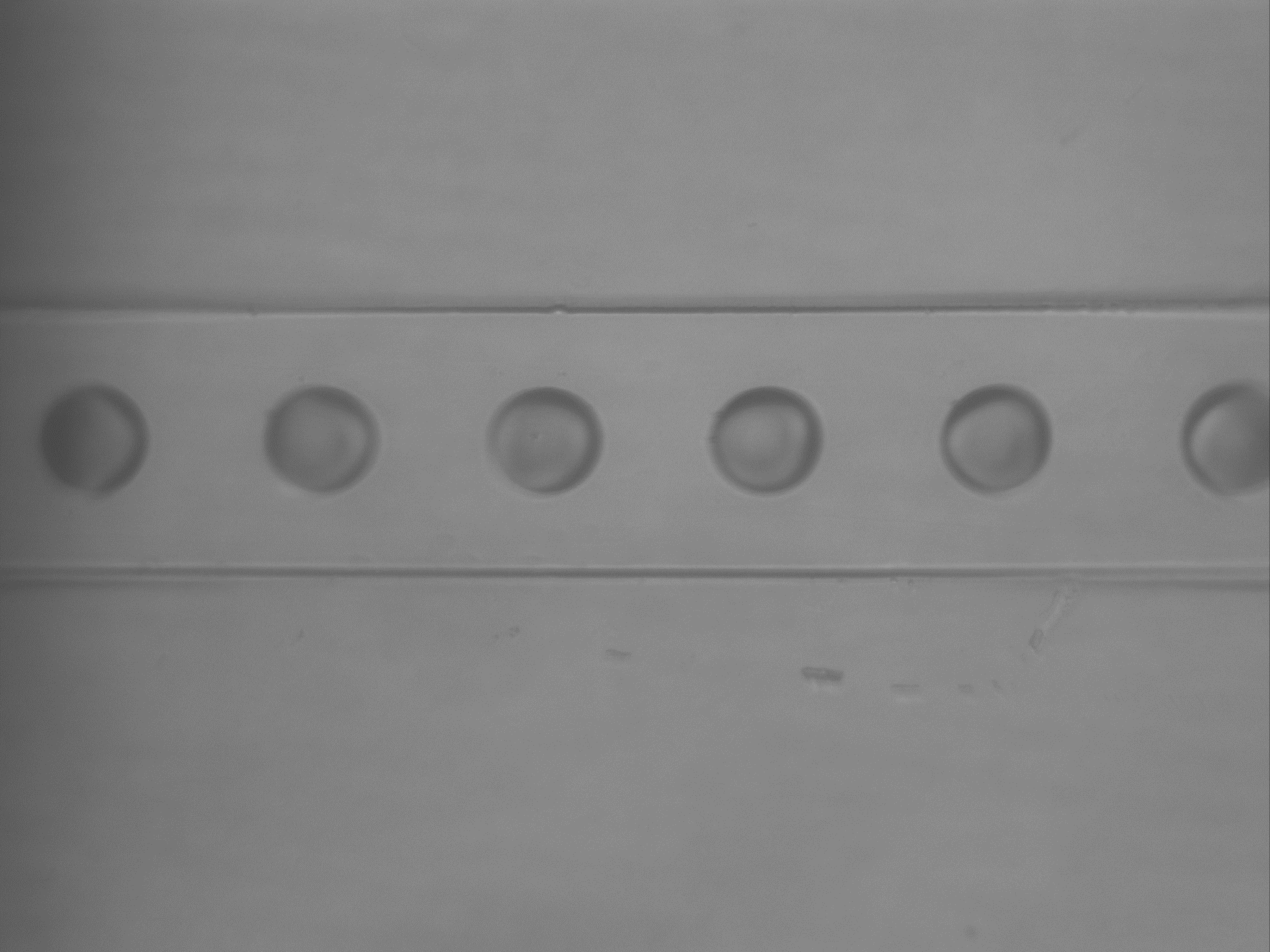 | 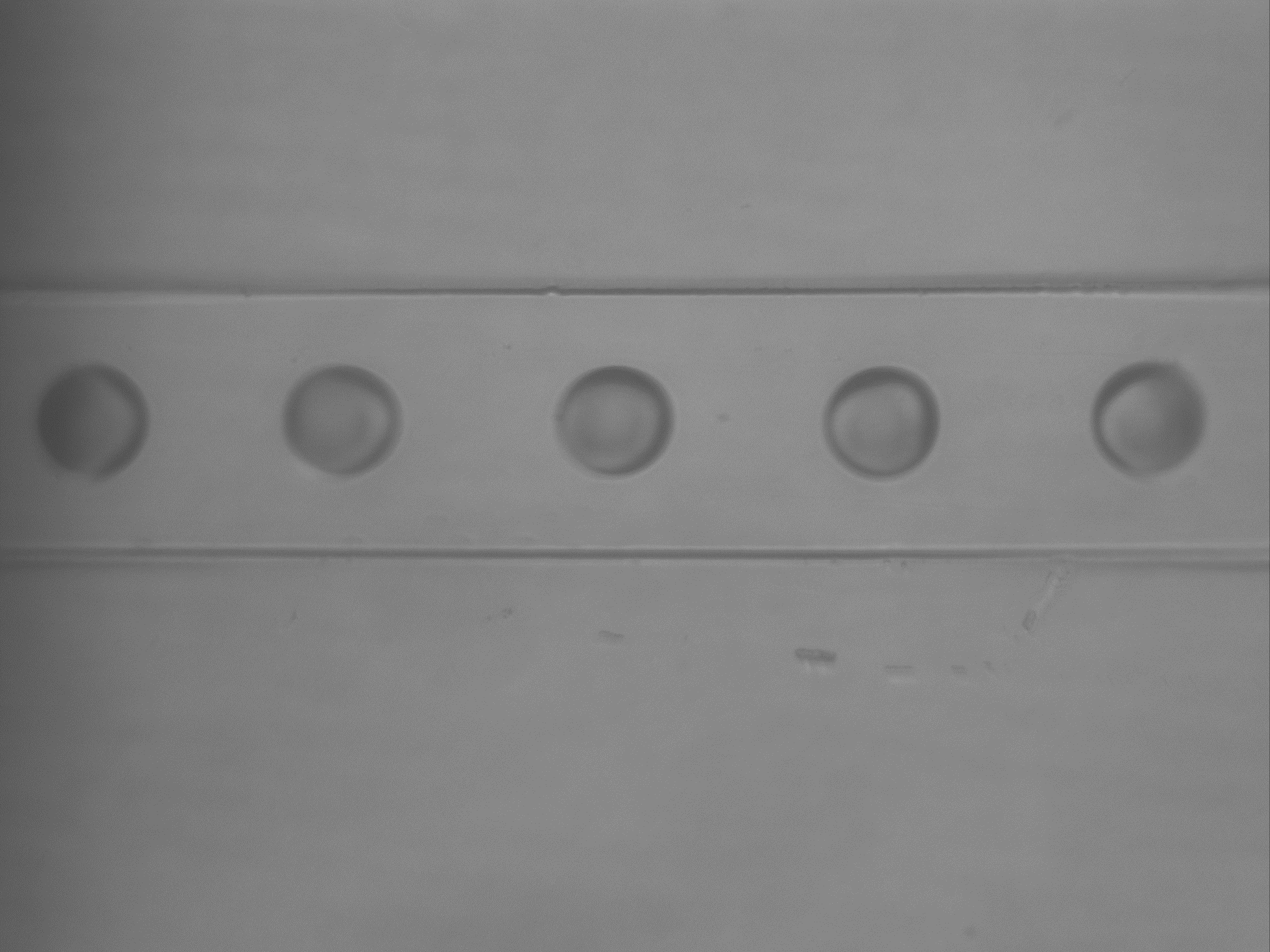 | 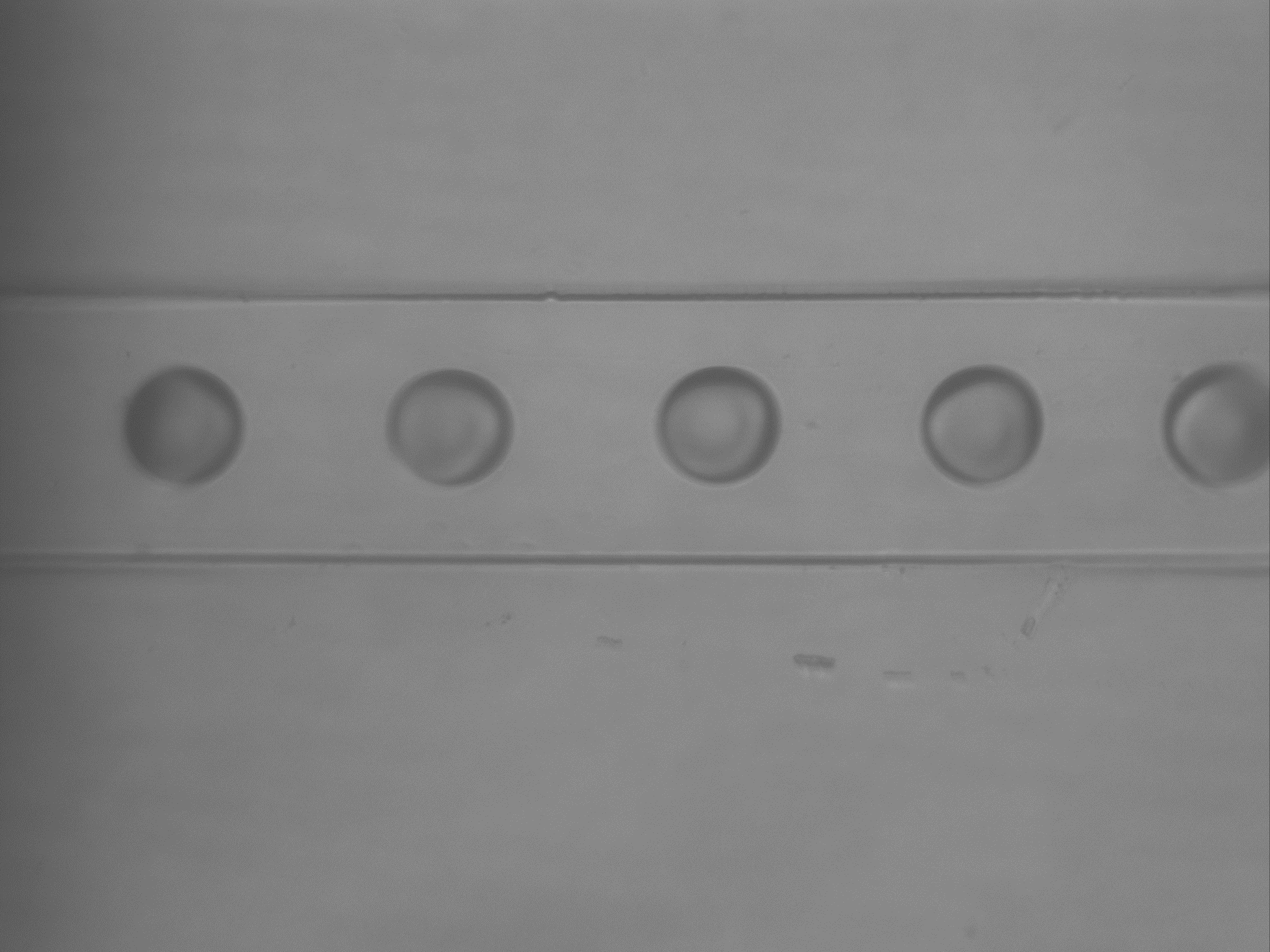 | 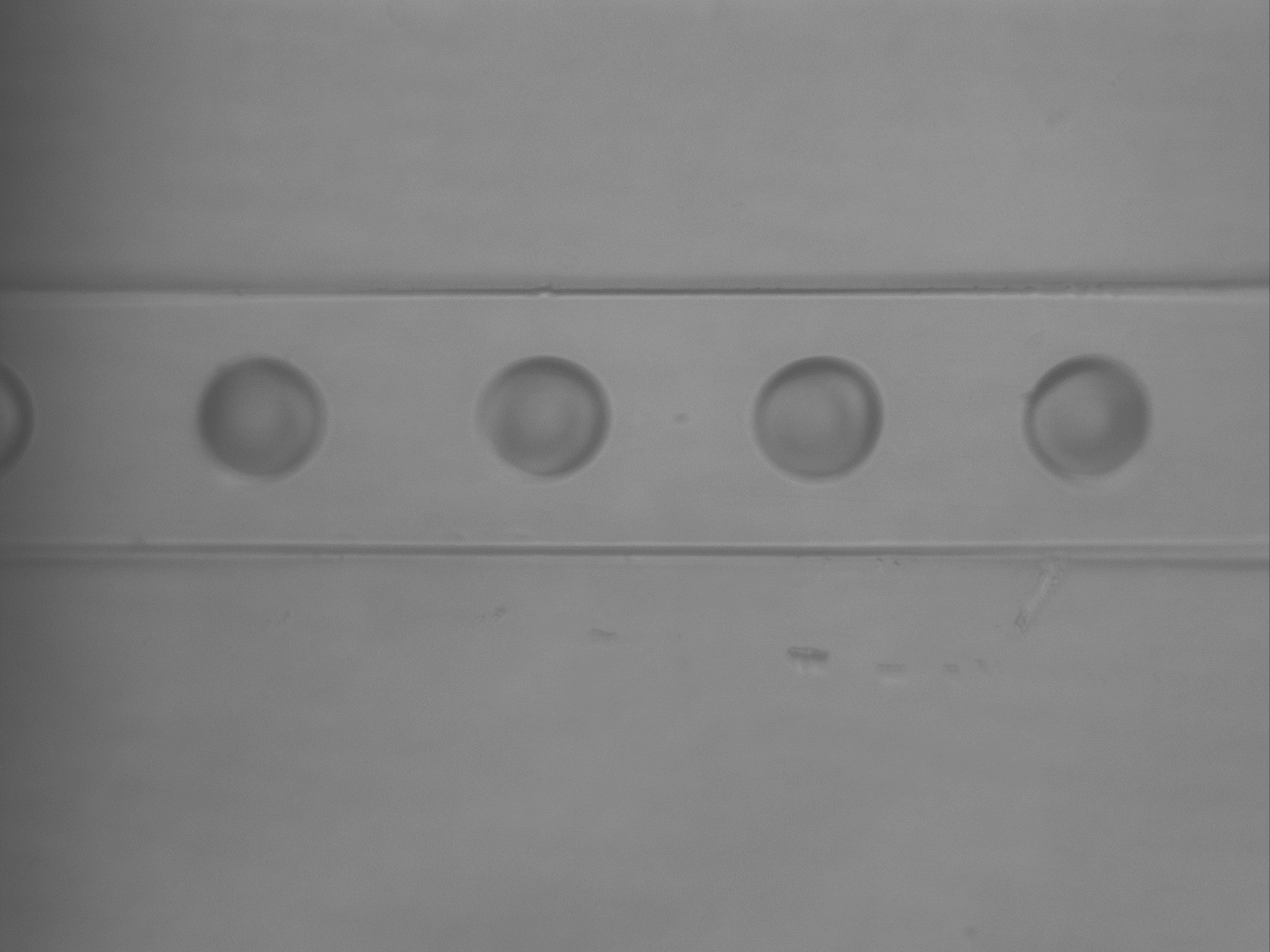 | 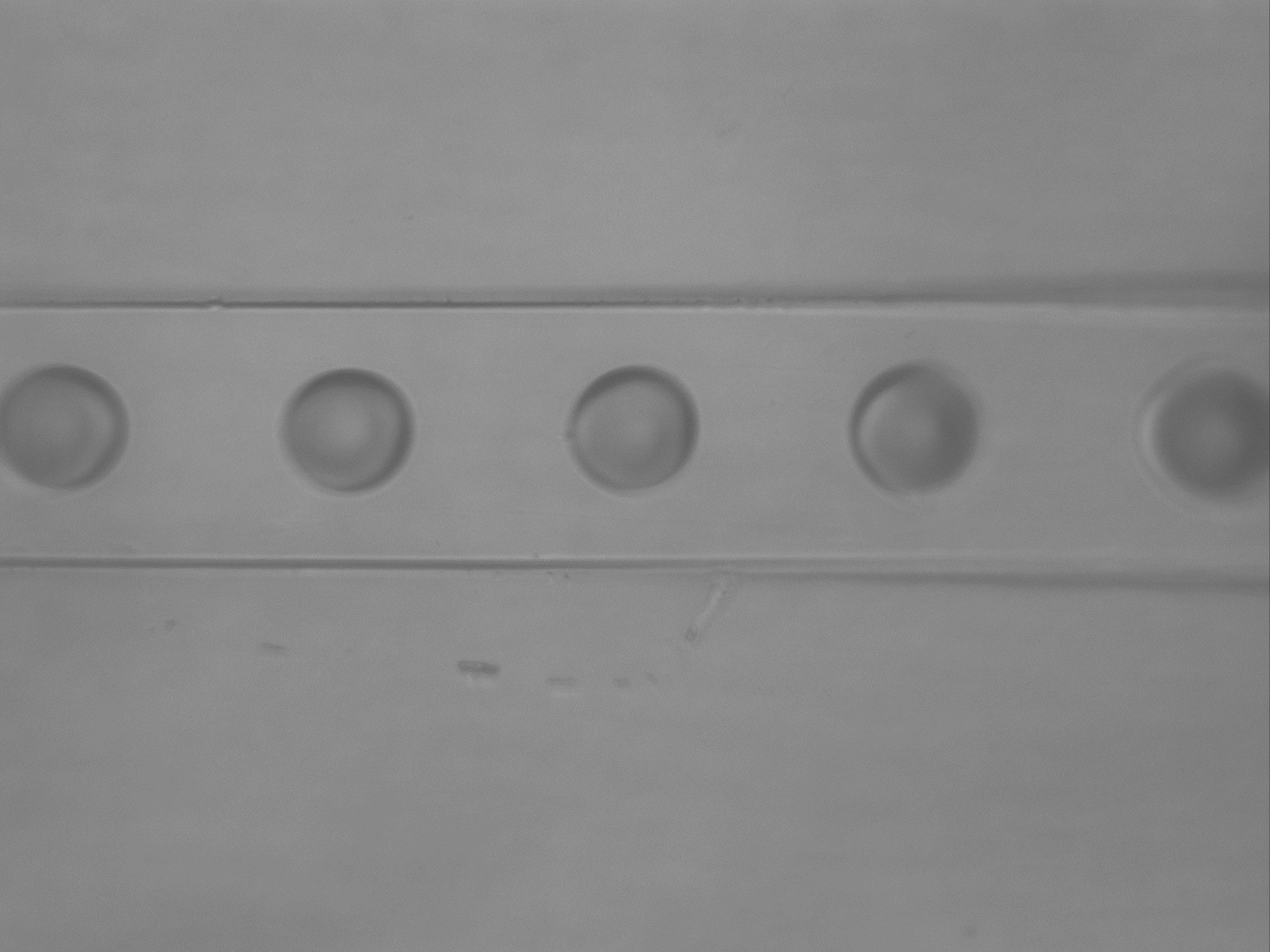 |
| 0.13 | 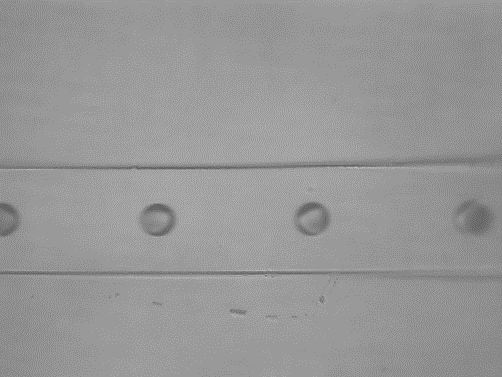 | 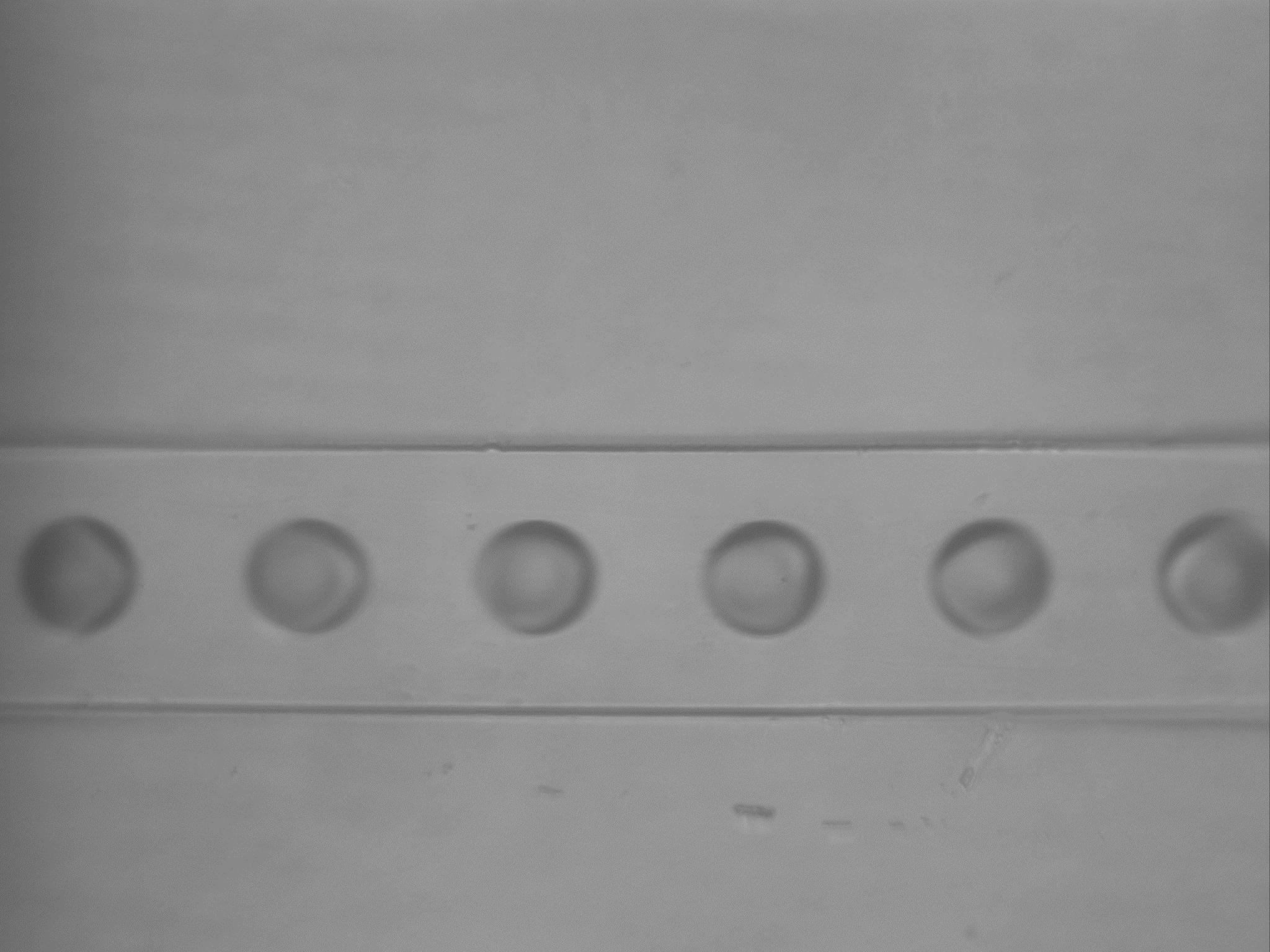 | 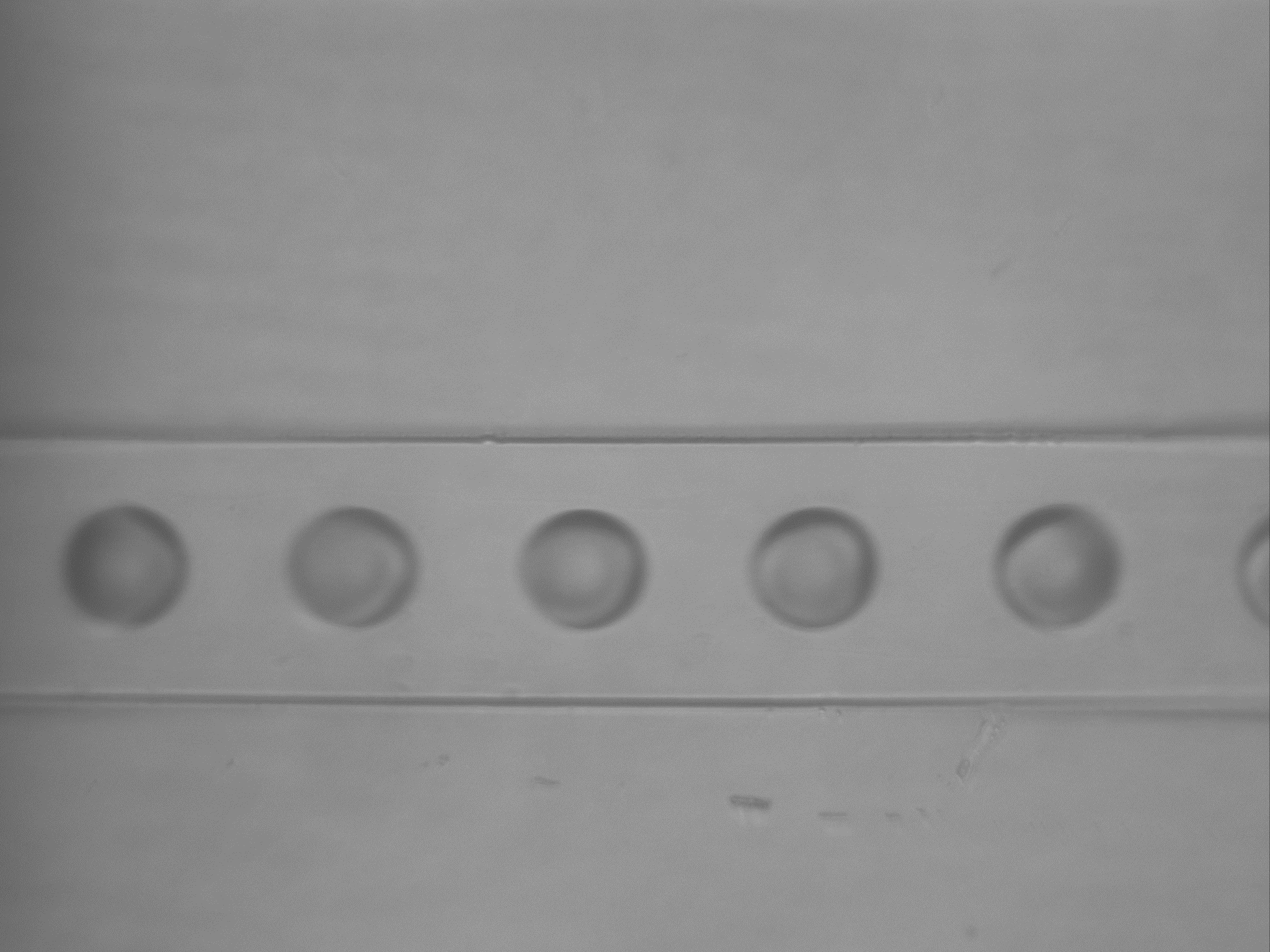 | 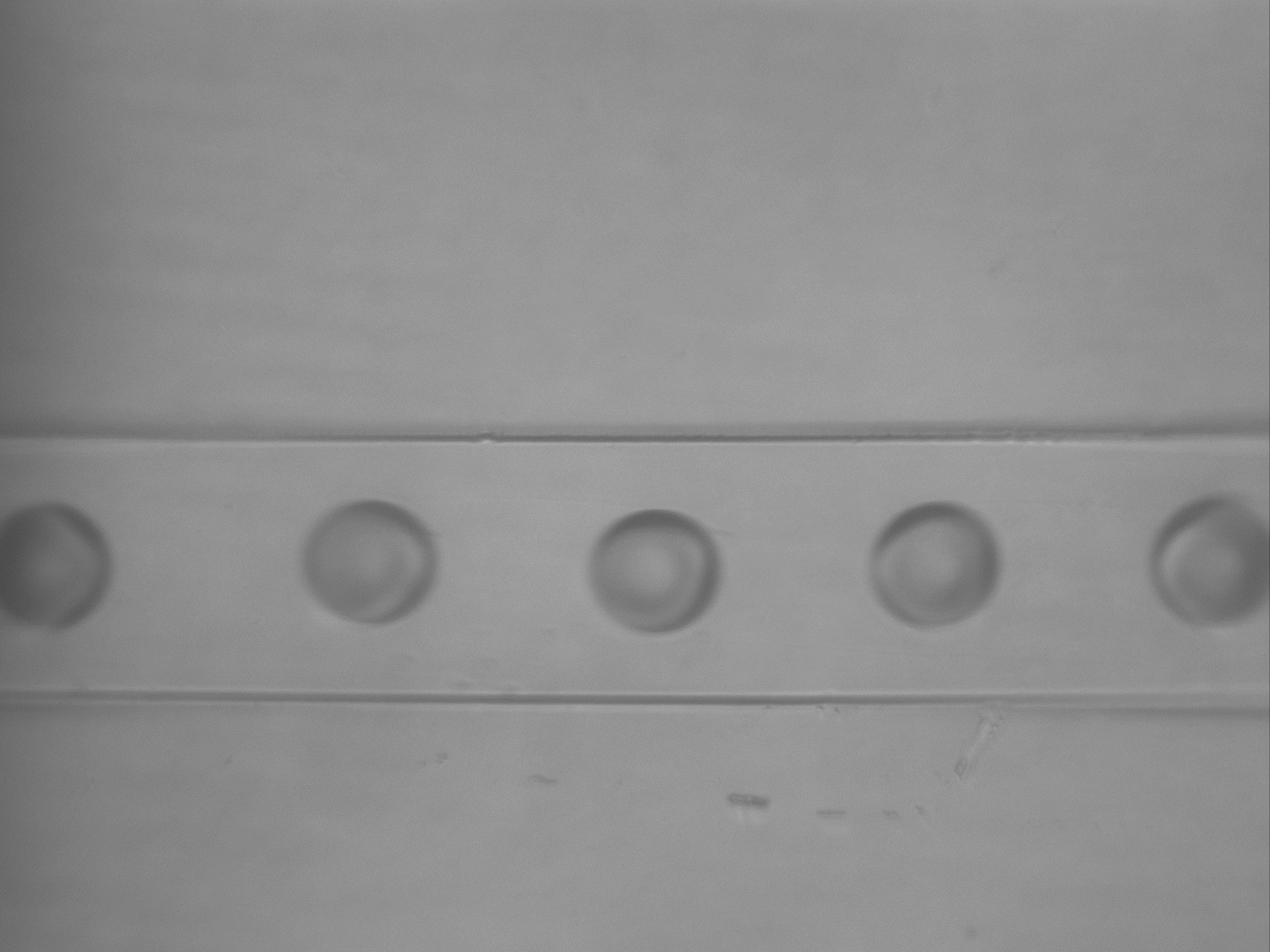 | 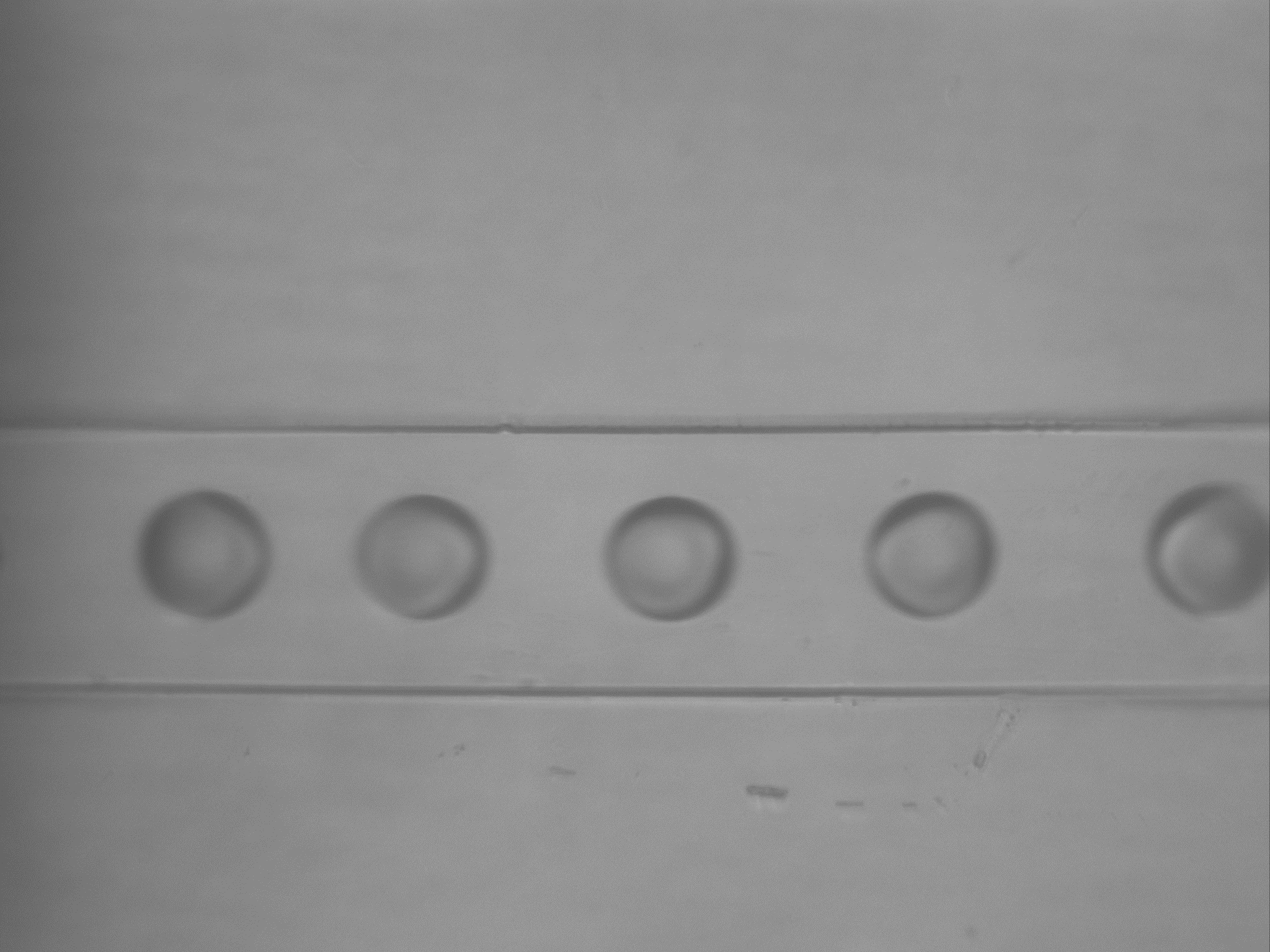 | 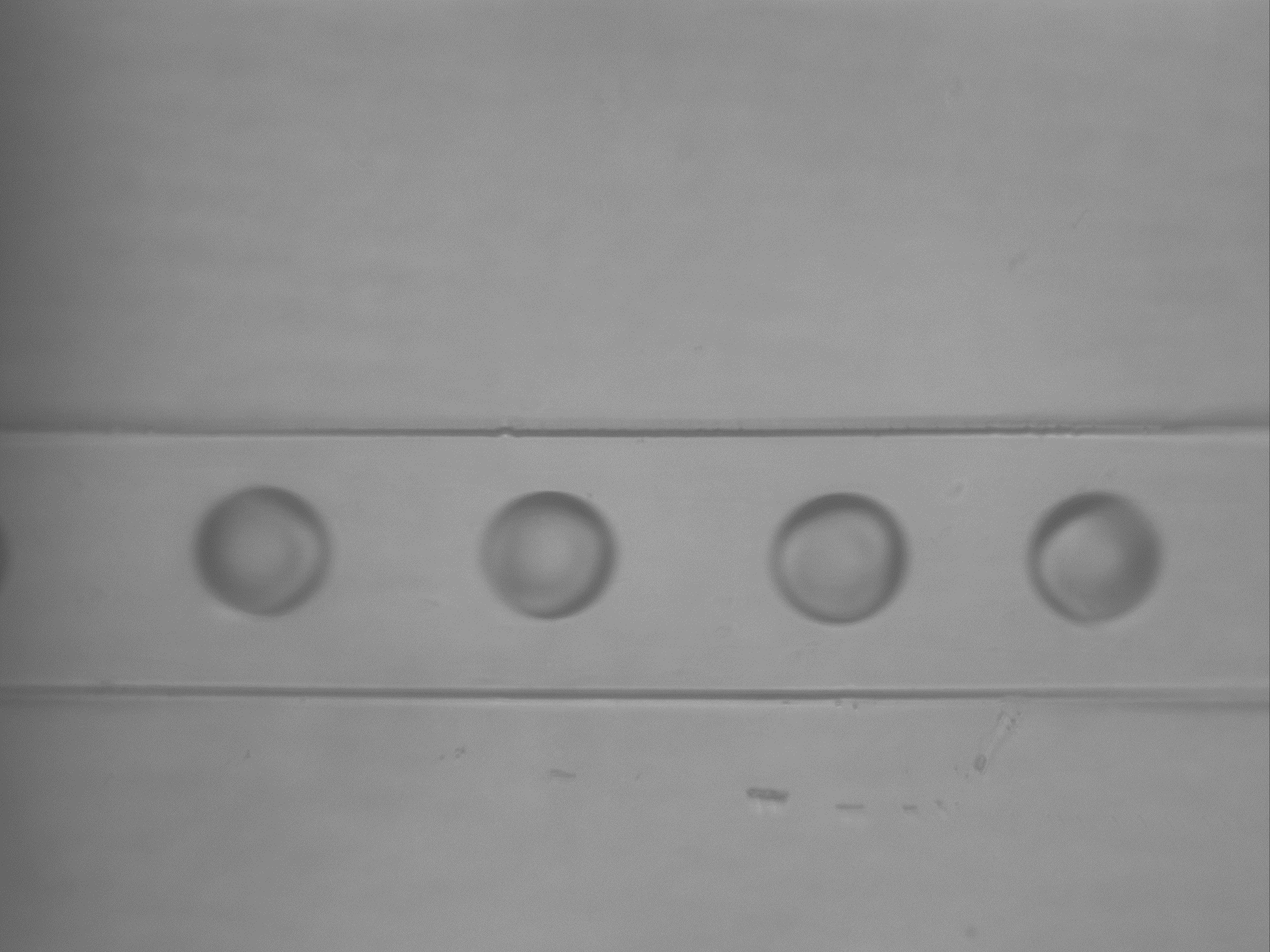 | 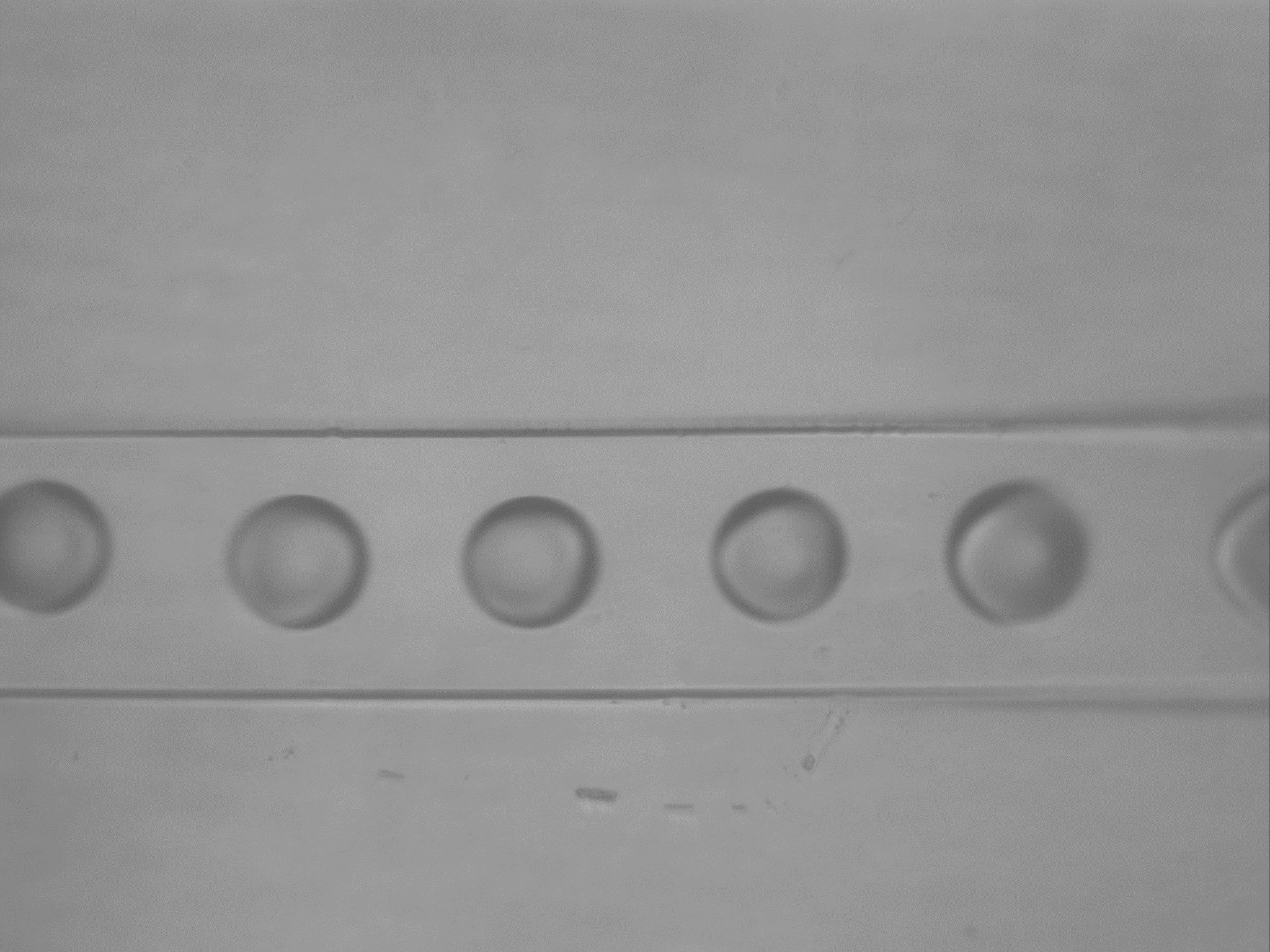 |
| 0.15 | 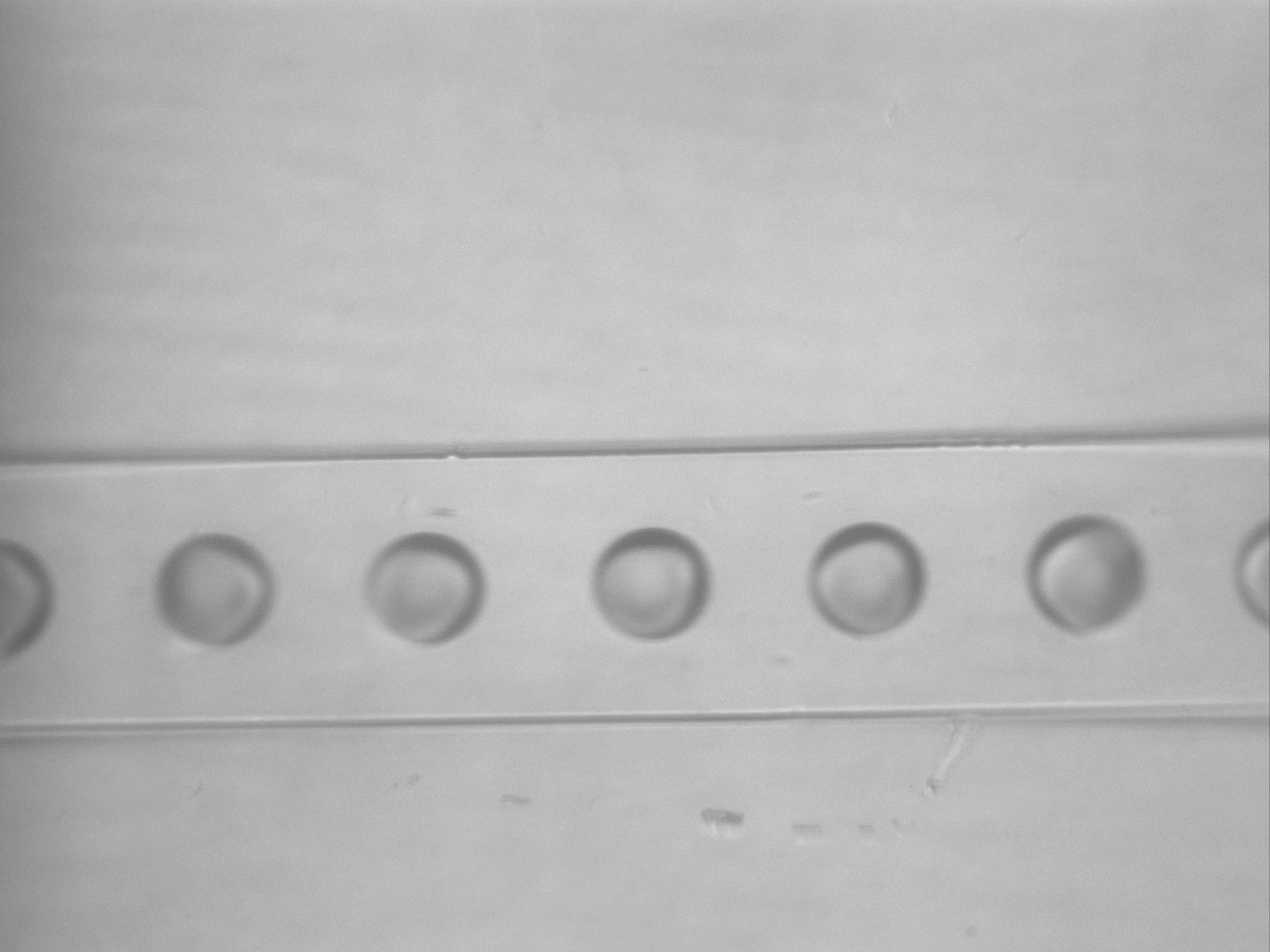 | 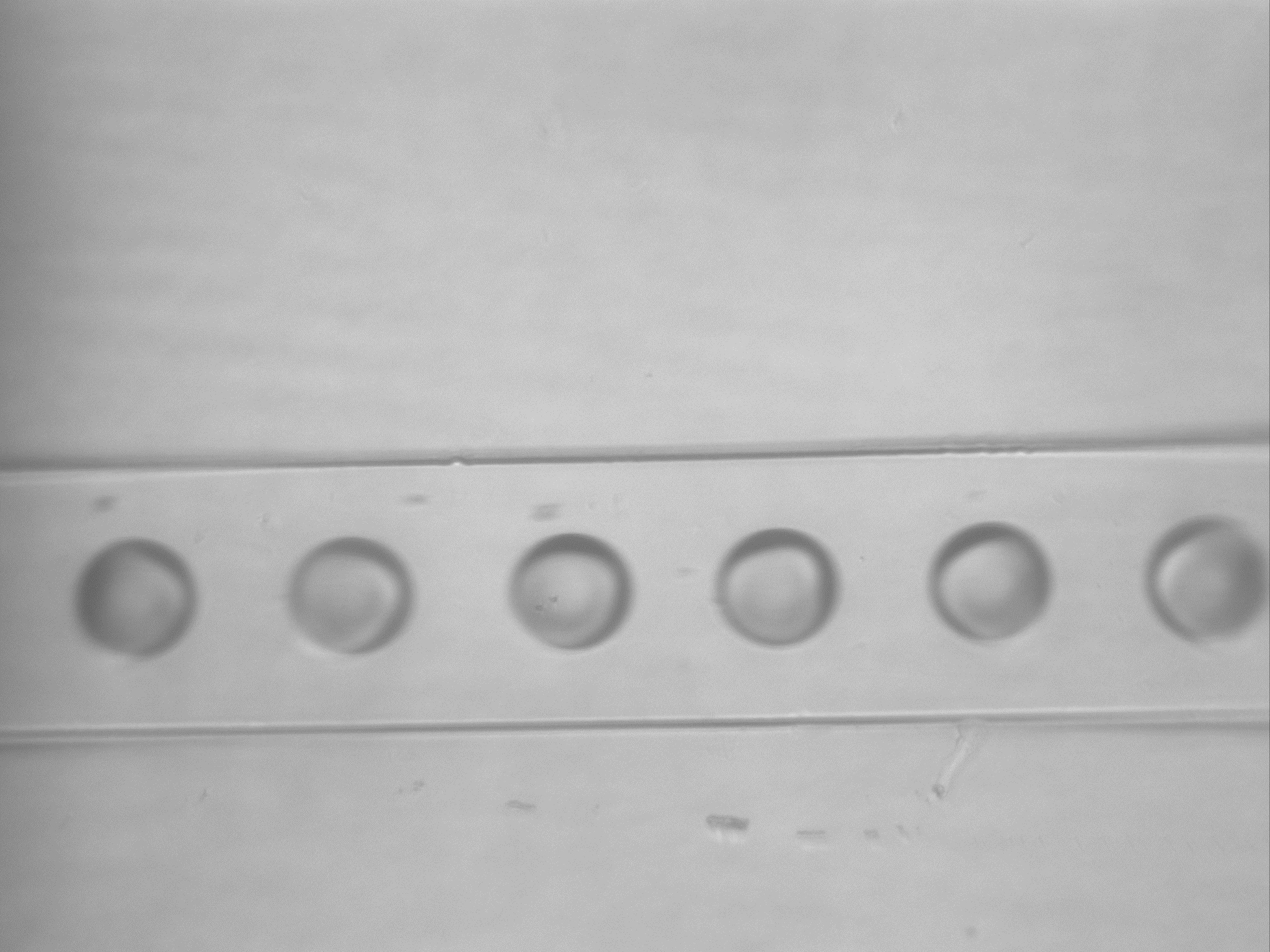 | 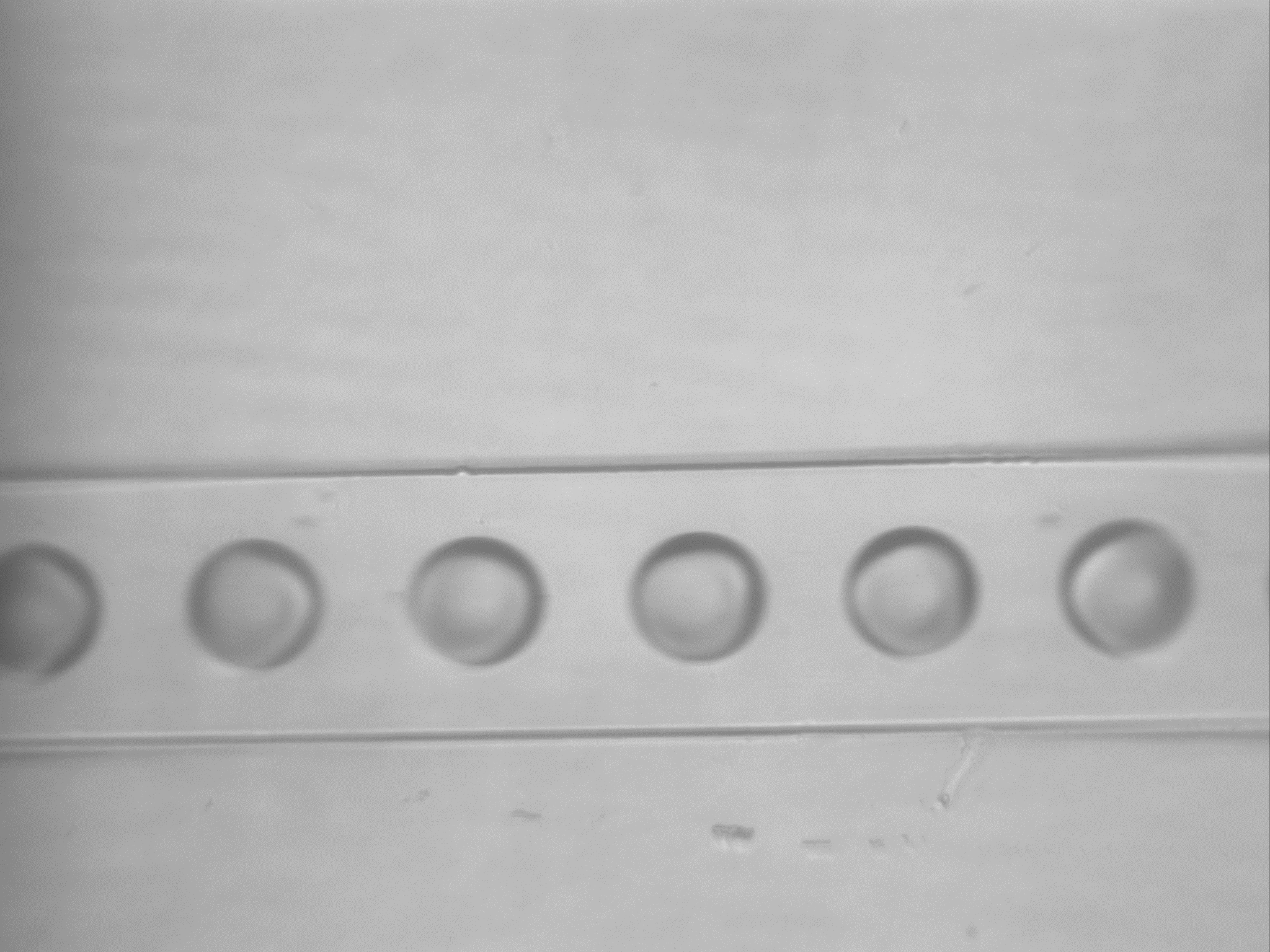 | 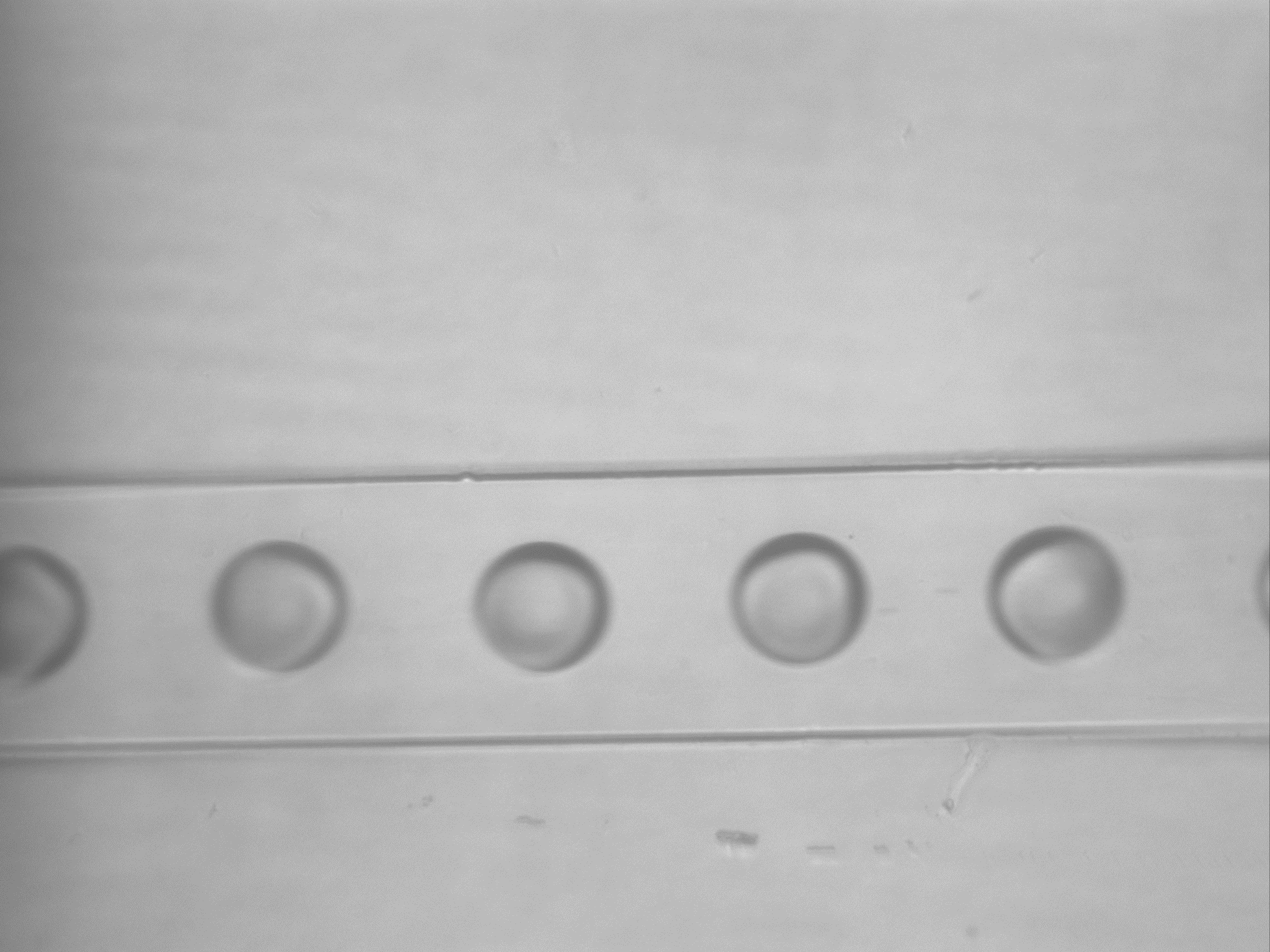 | 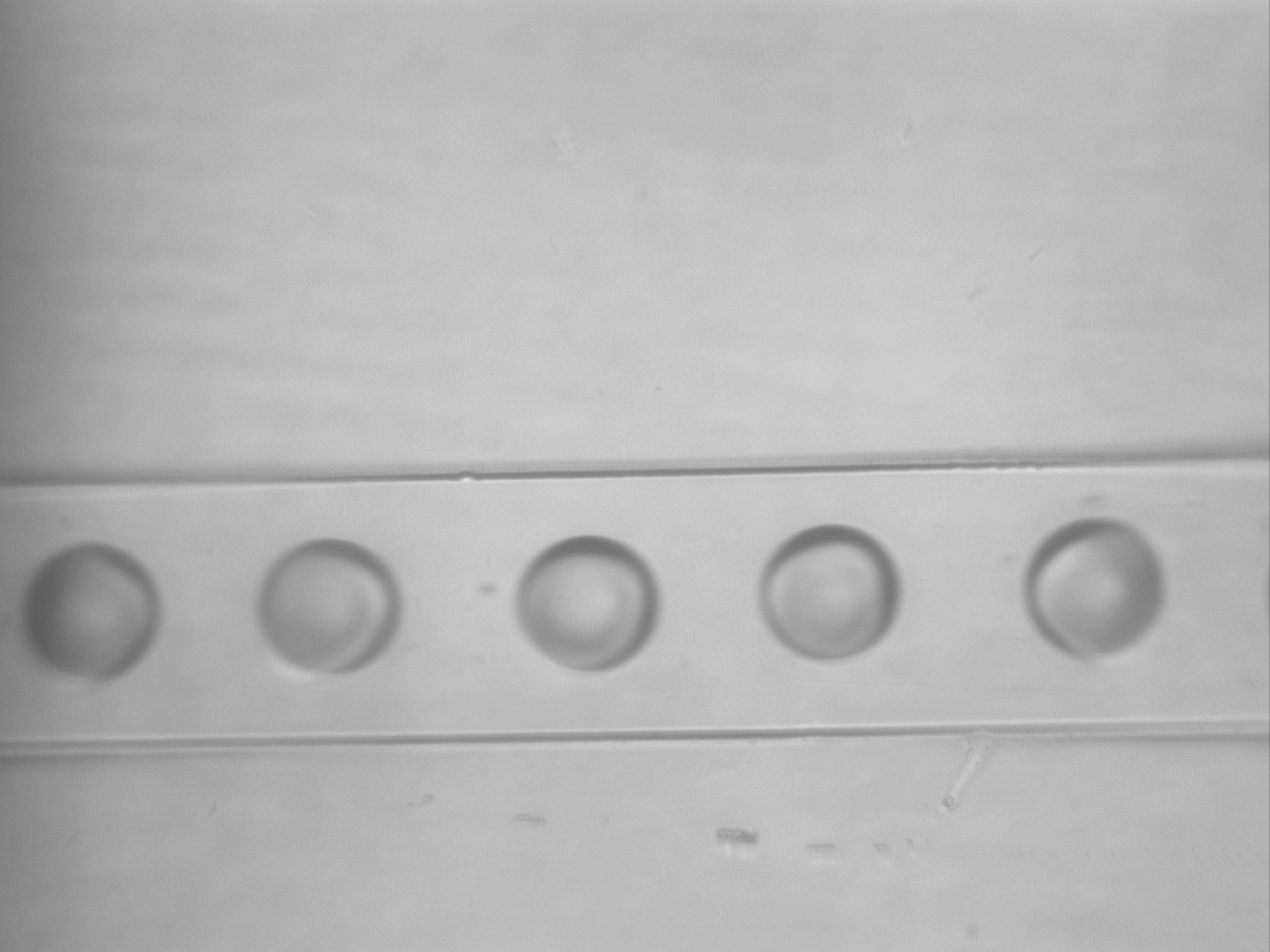 | 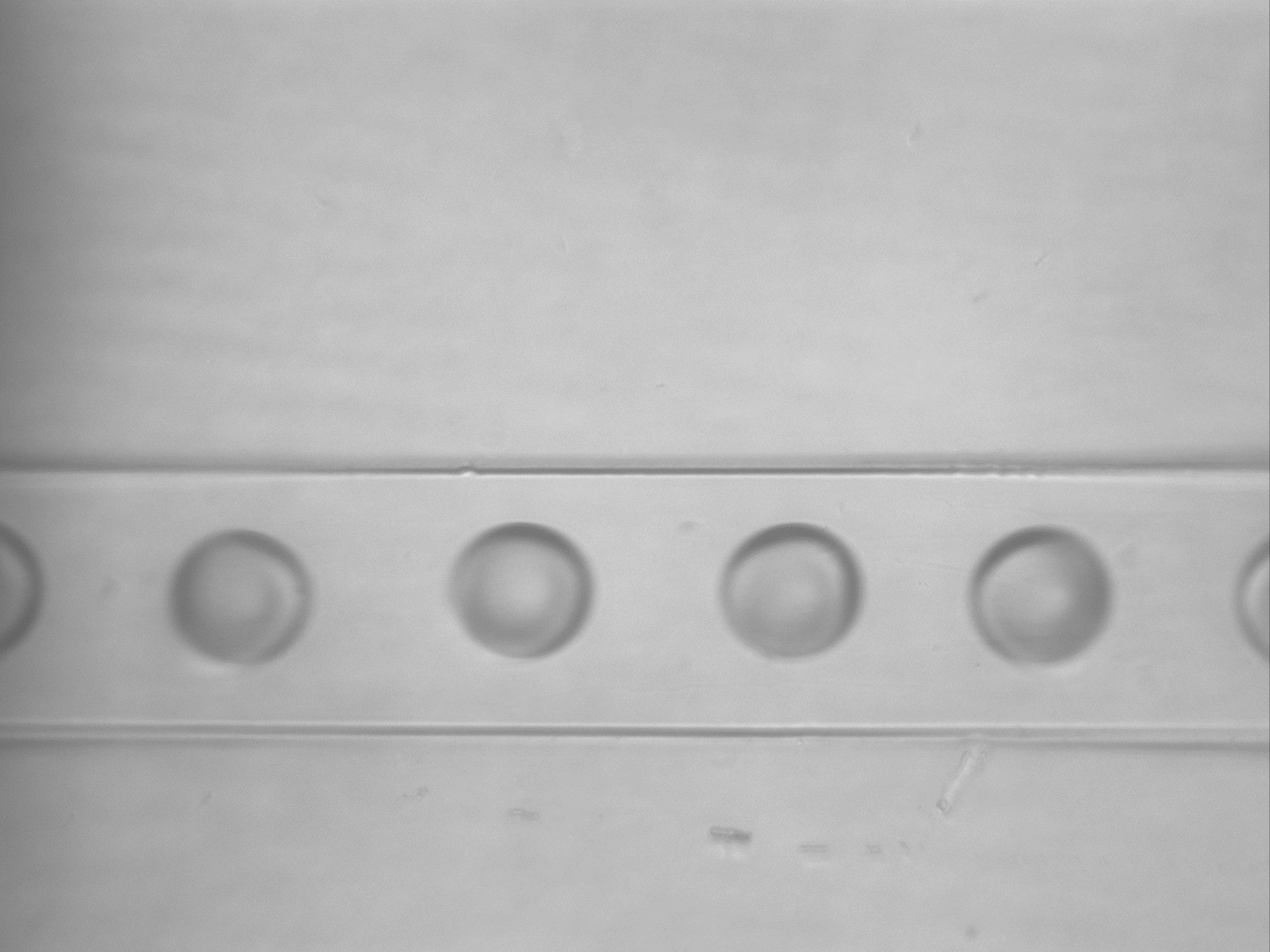 | 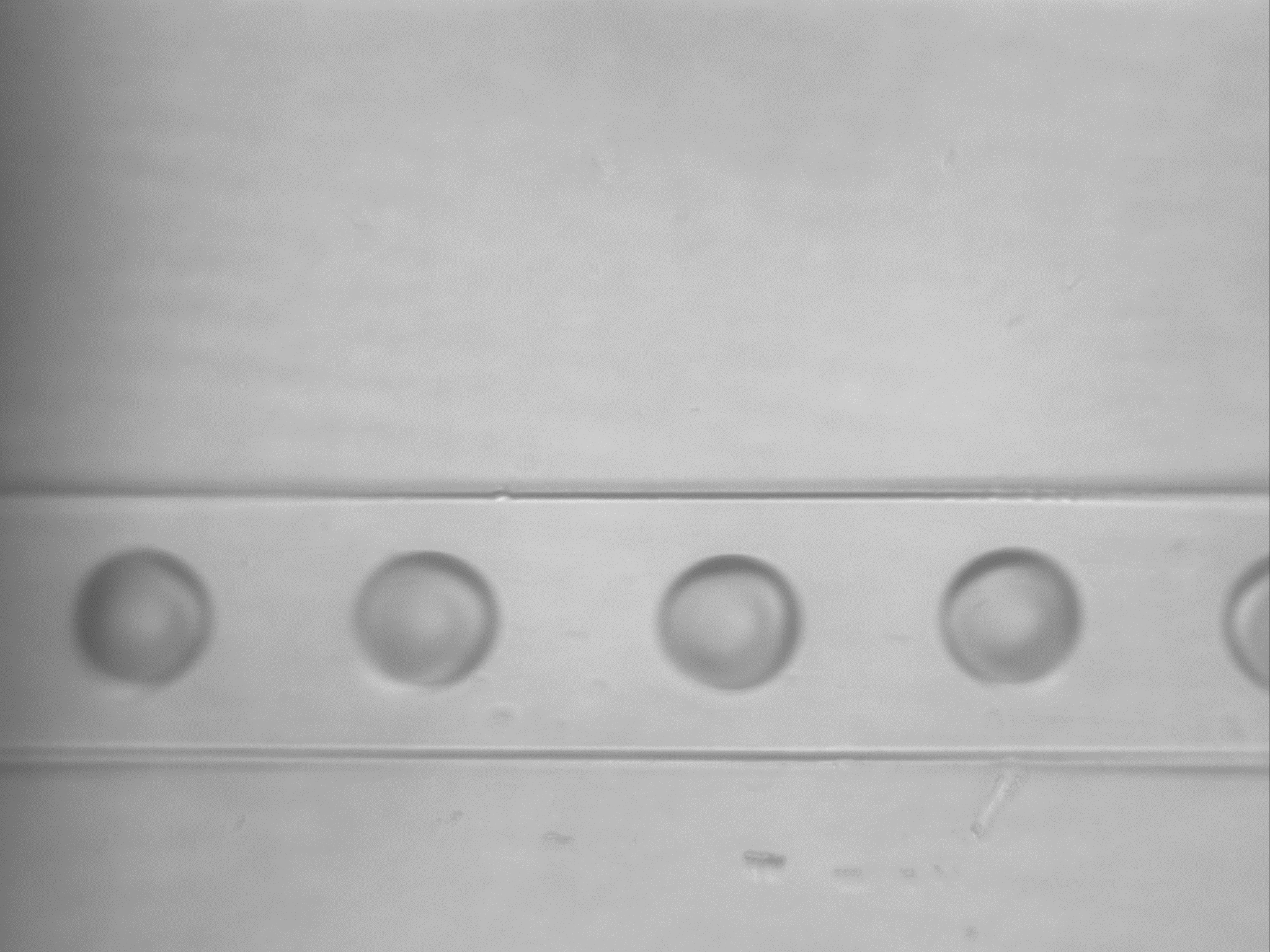 |
| 0.20 | 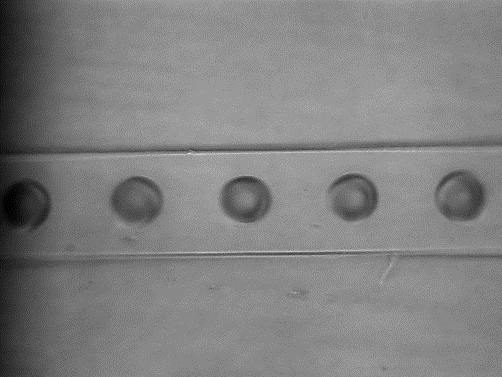 | 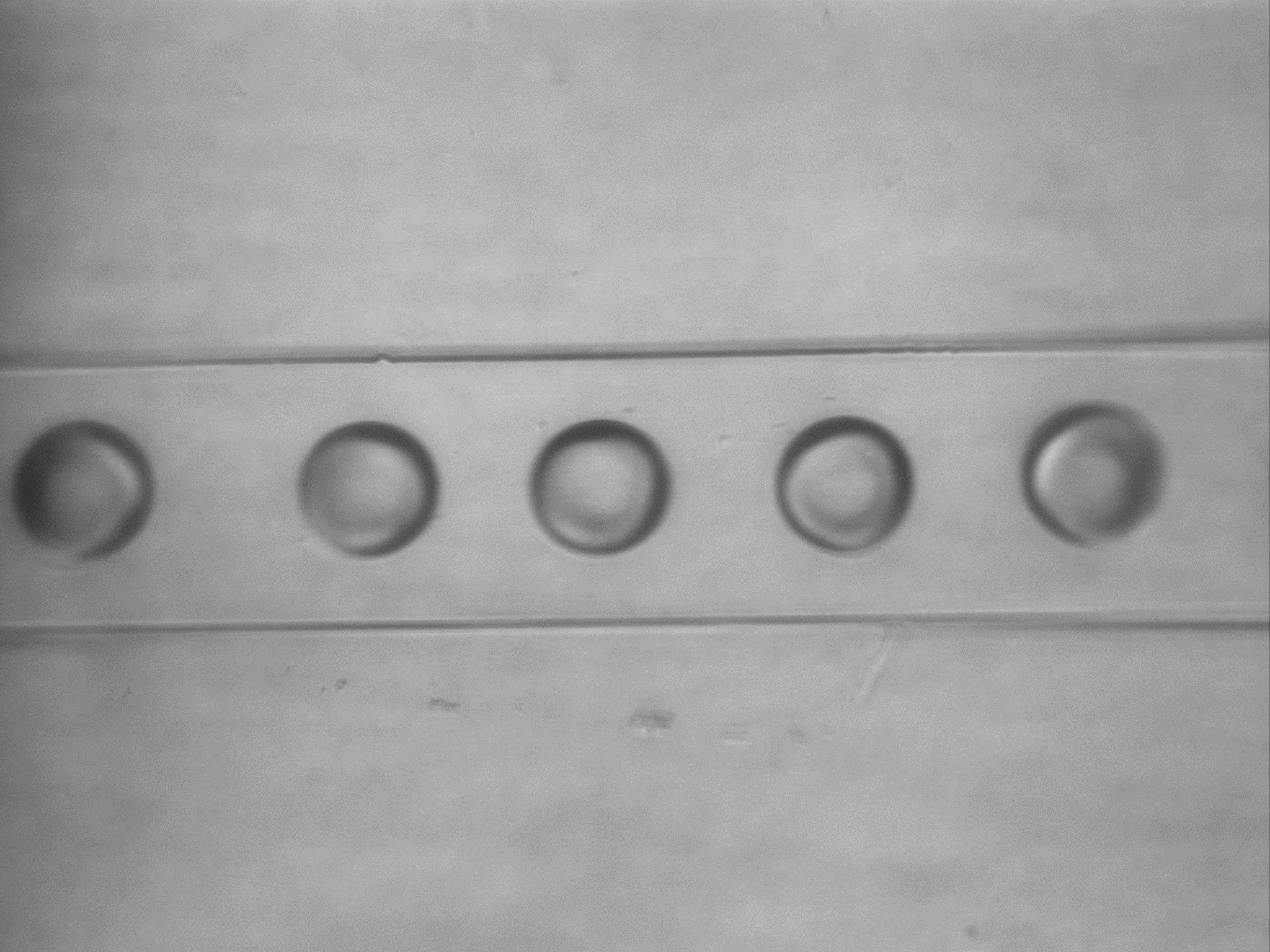 | 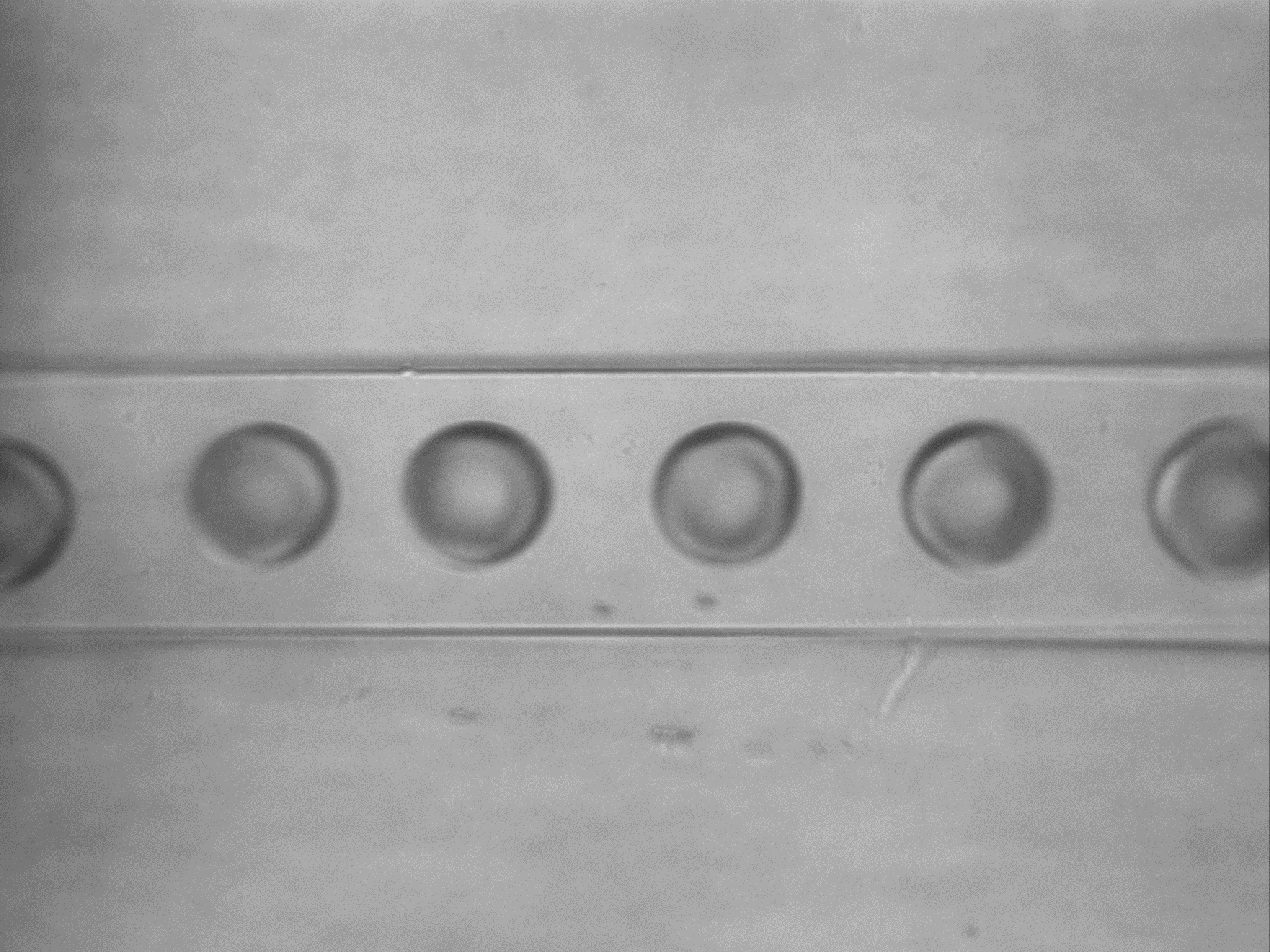 | 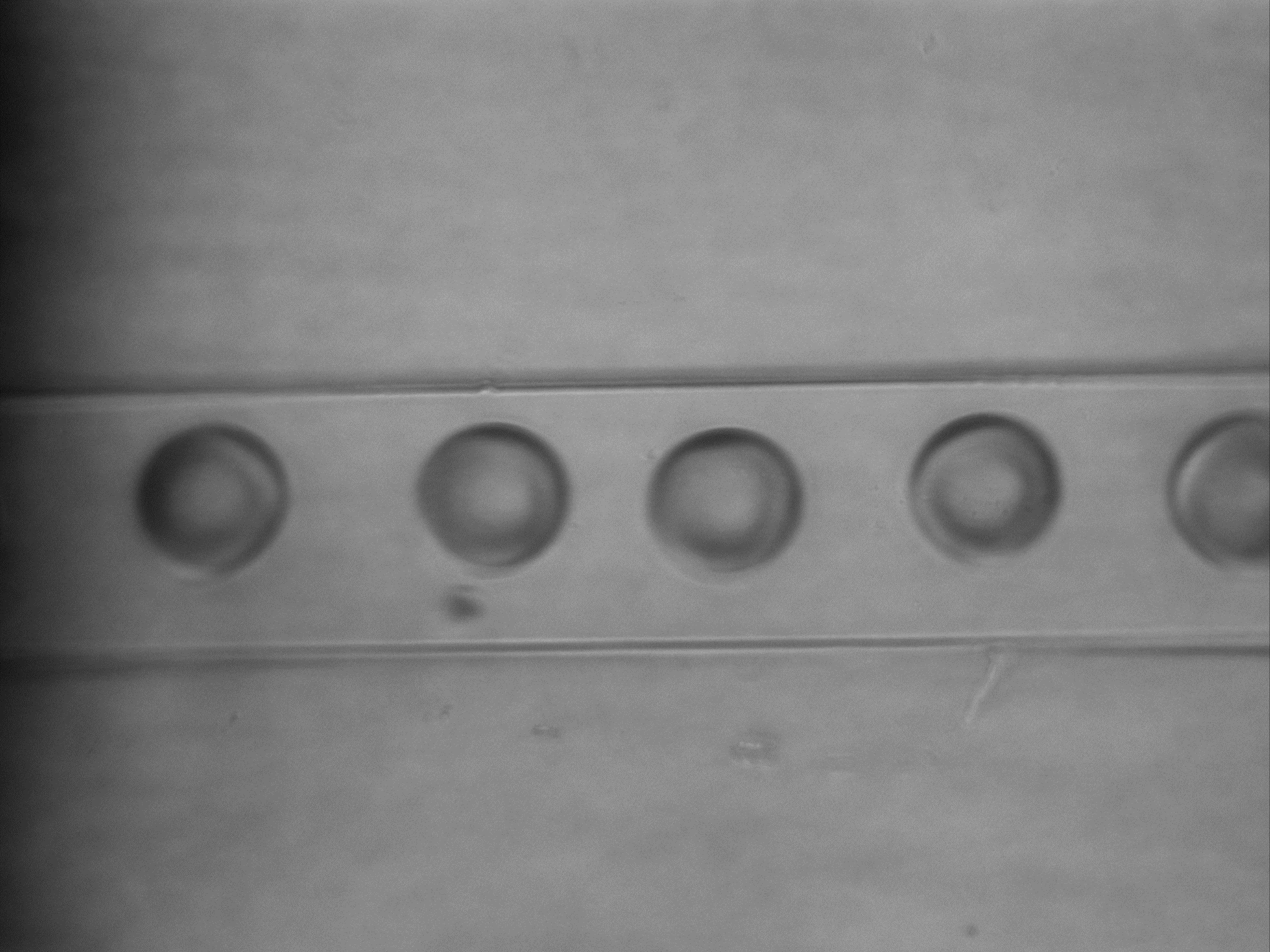 | 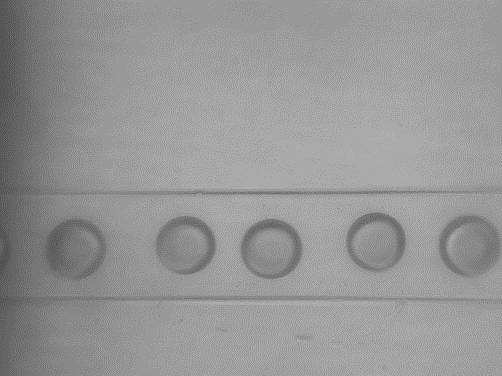 | 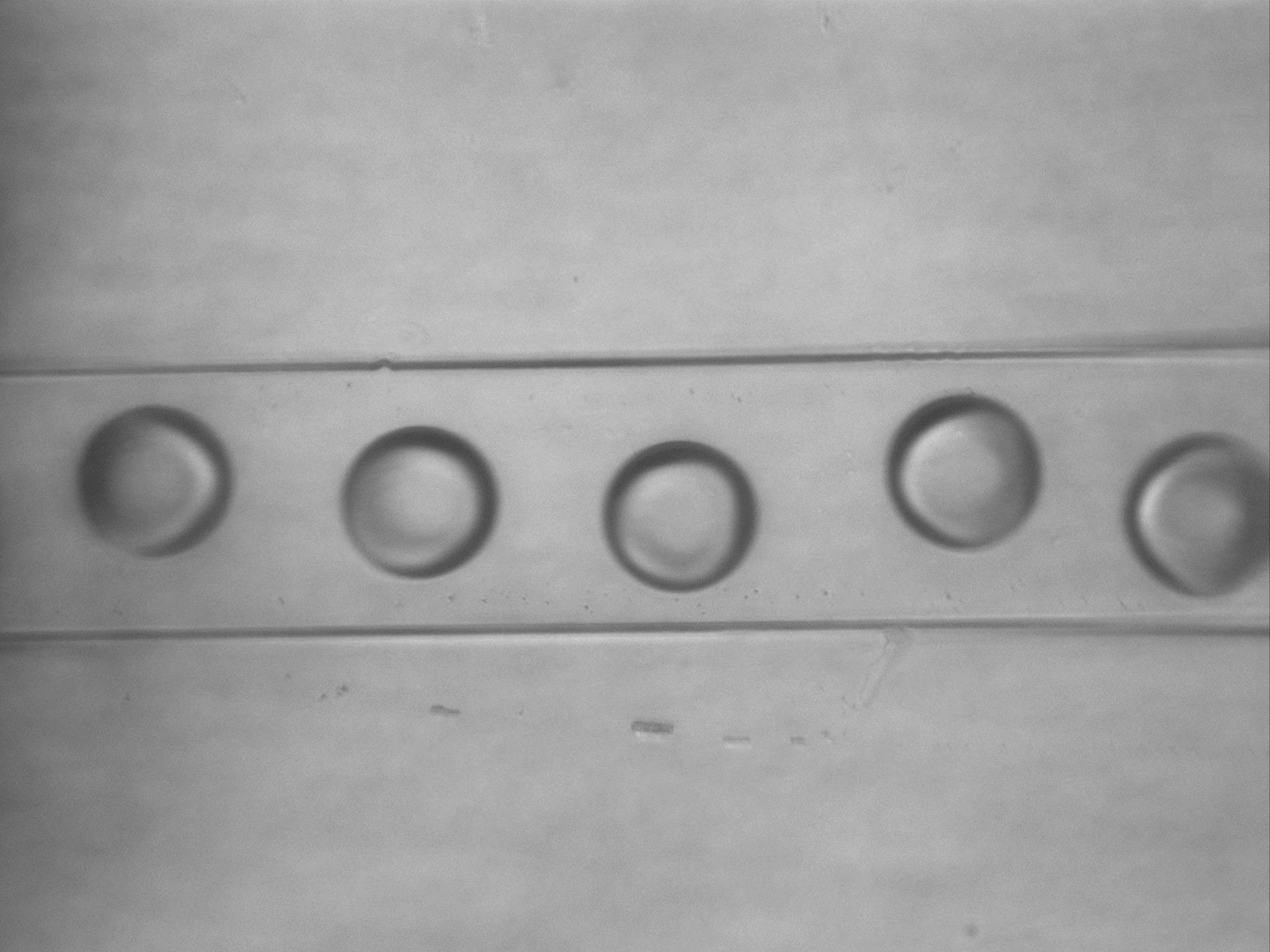 | 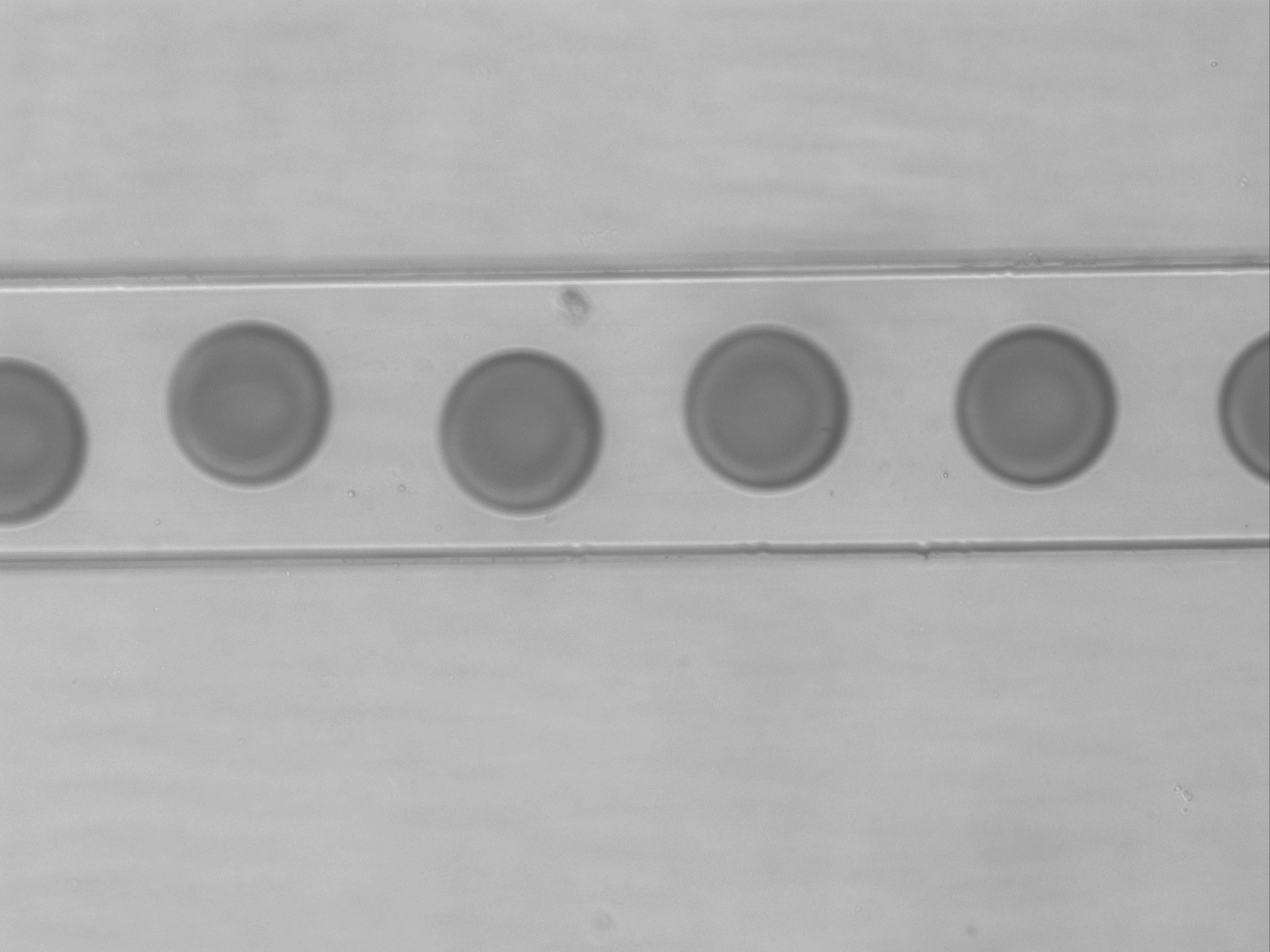 |
| 0.25 | 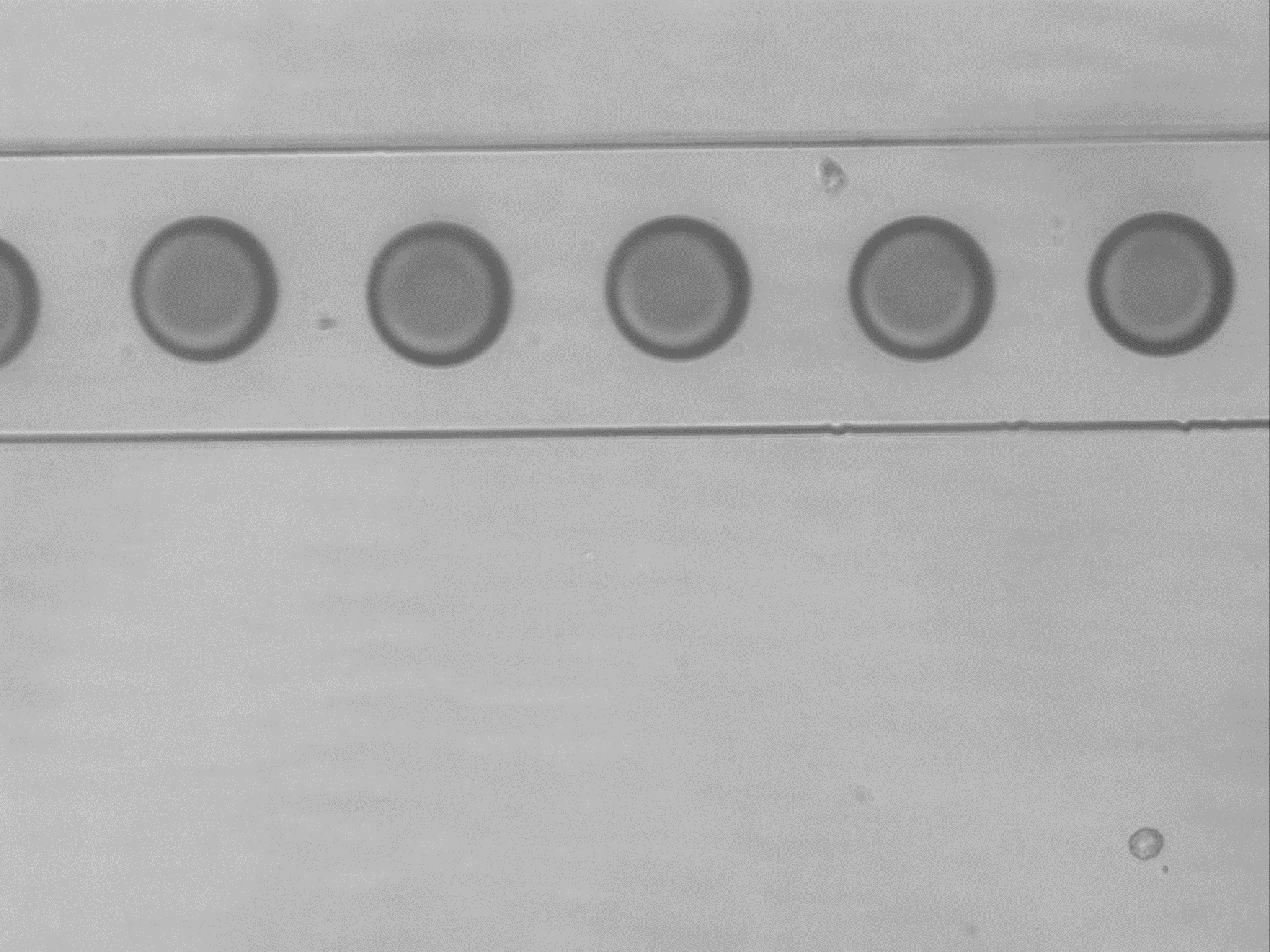 | 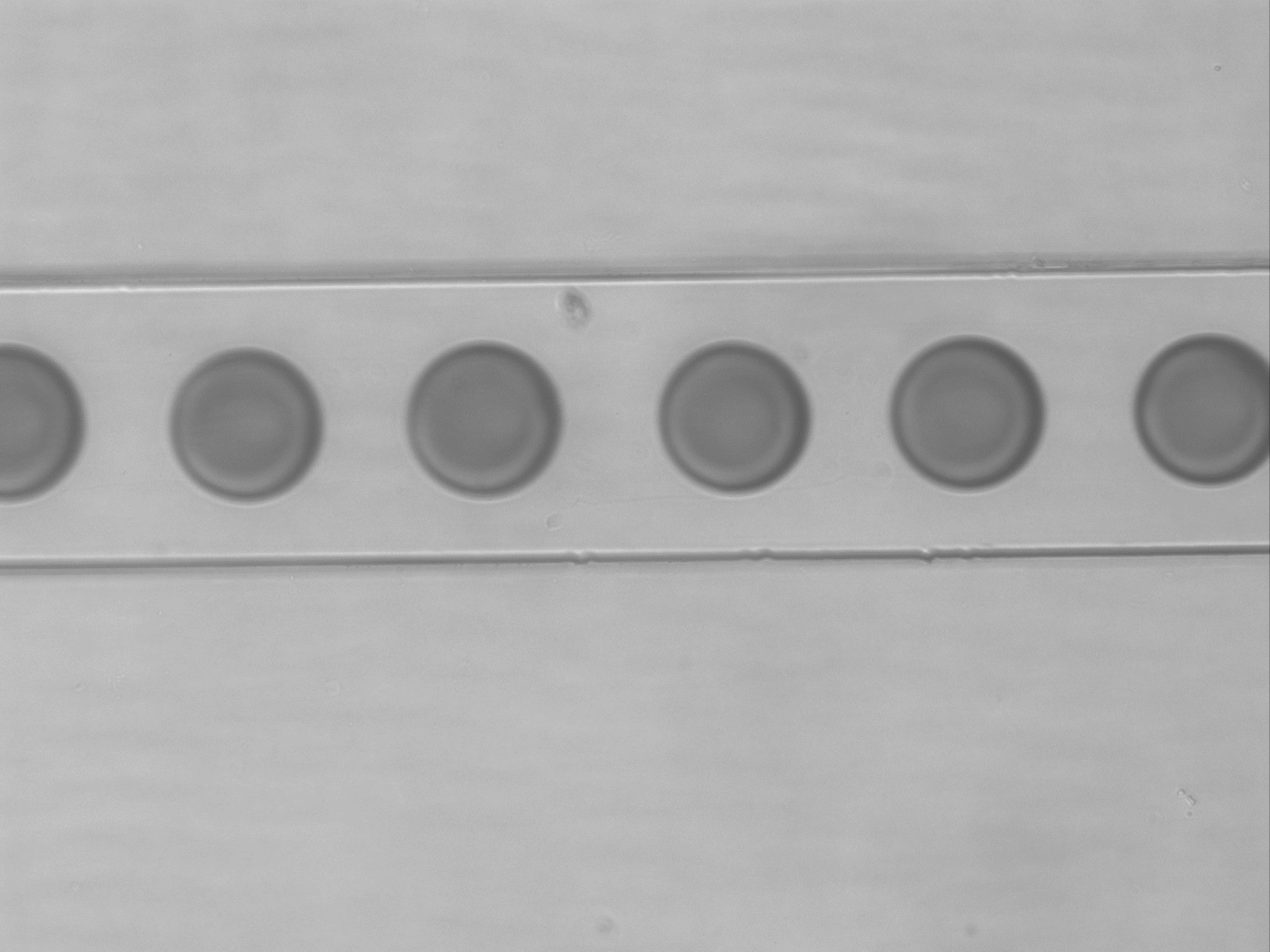 | 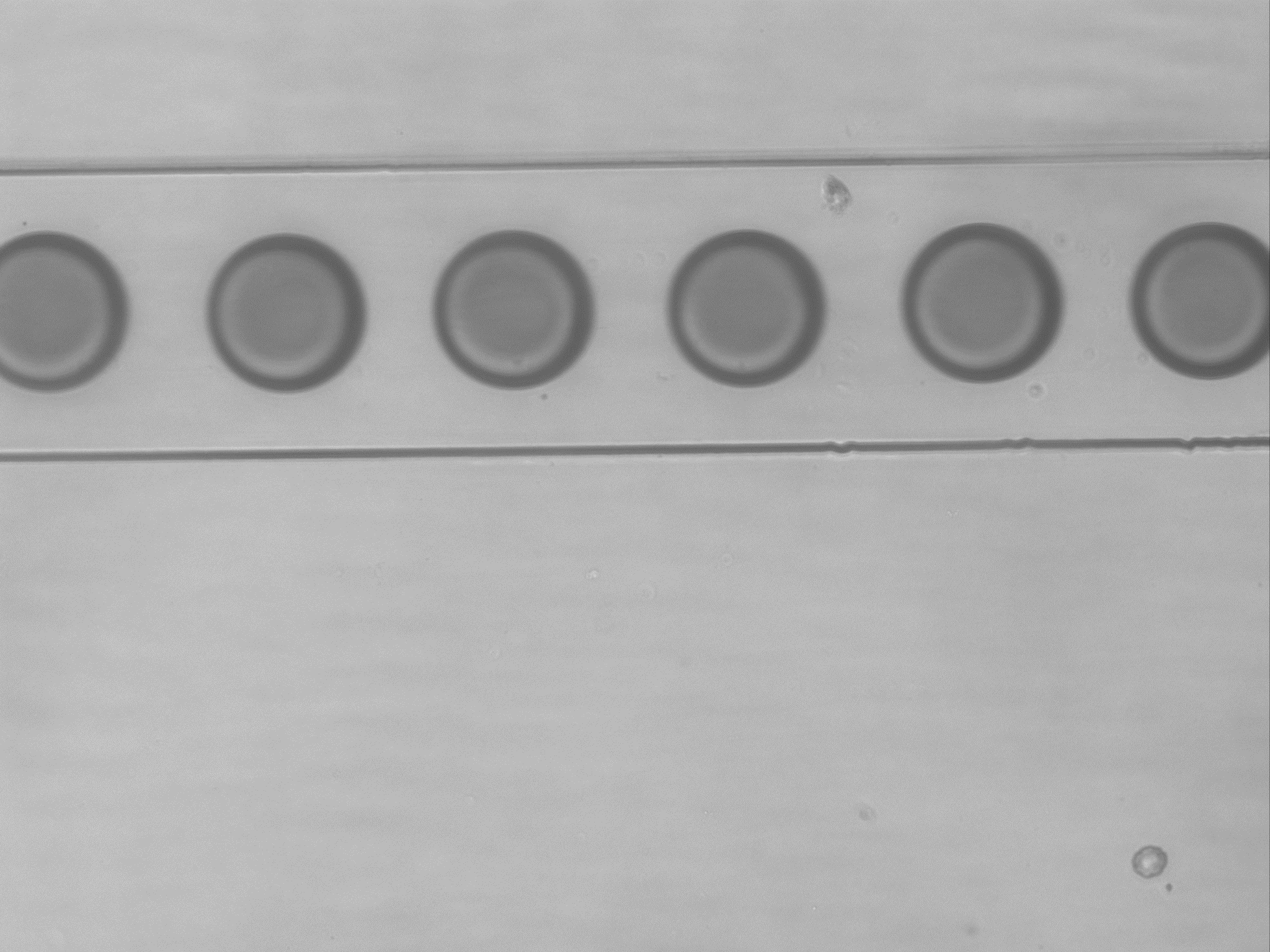 | 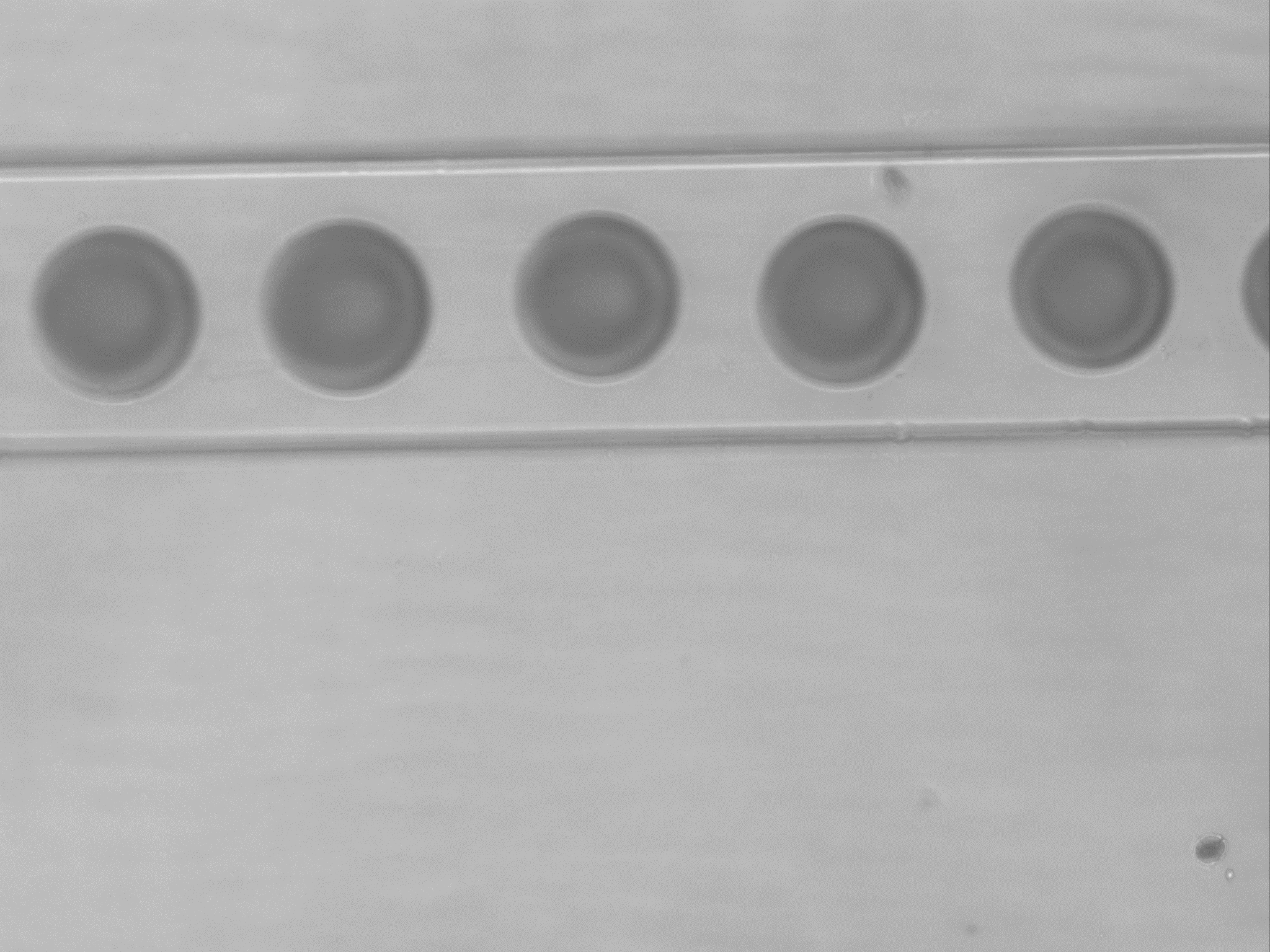 | 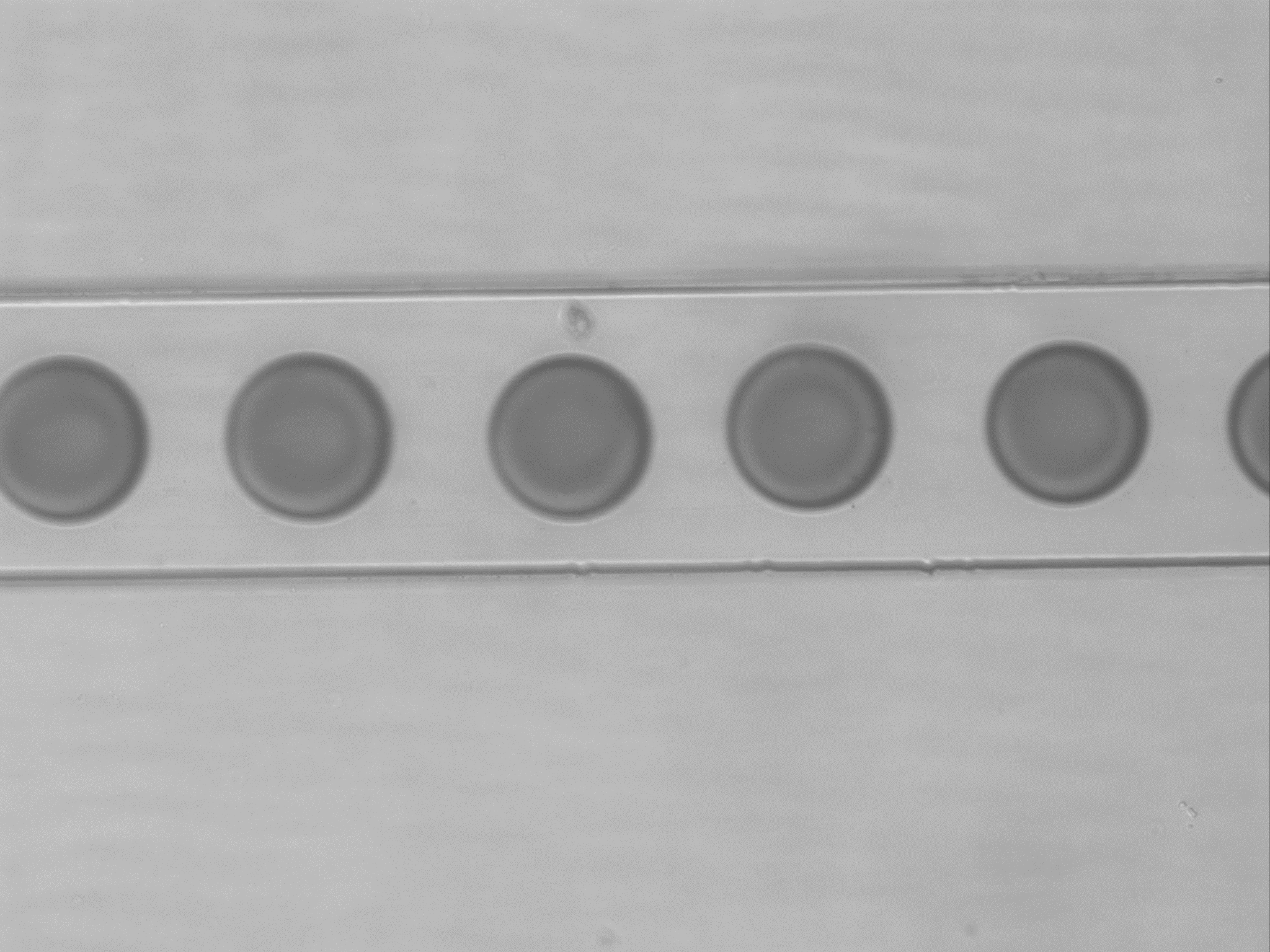 | 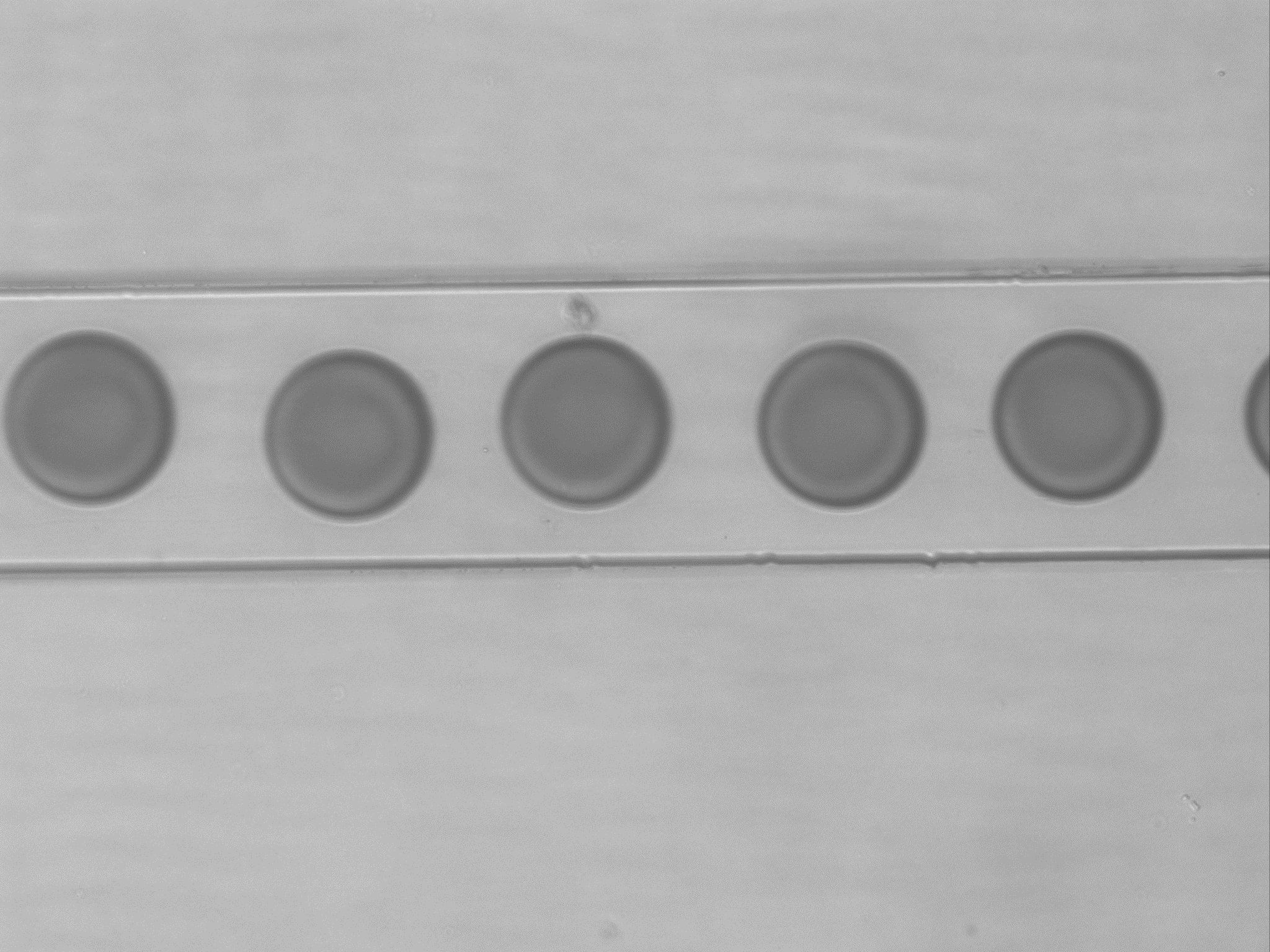 | 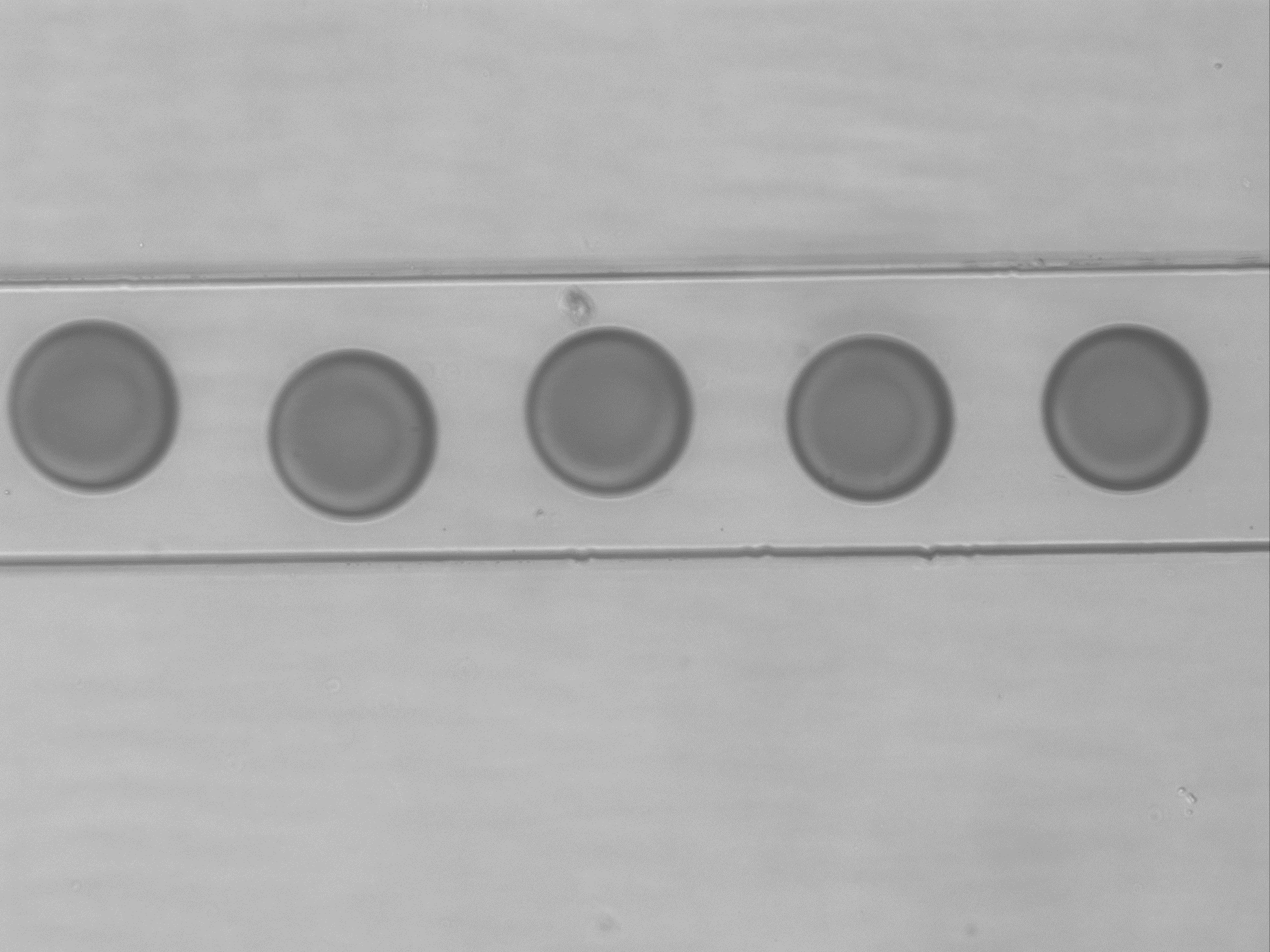 |

## S3. Relative error calculated from correlations

The relative error defined by equation S2 is calculated between the experimental data and the correlations obtained from $f_{1}\left( \varphi\right)$in equation S1. The errors are presented for the experiments performed in the absence and presence of the surfactant.

$f_{1}\left( \varphi\right)= \left\{ \begin{aligned} 0.75\varphi^{0.37} , pure Oil \\ \\ 0.83\varphi^{0.37},Oil with SPAN20 \end{aligned} \right.$ (S1)

$\% Relative Error = \frac{(Exp-Corr)}{Exp}\times100$ (S2)

**Table S3** Relative error calculated from $f_{1}\left( \varphi\right)$ respect to the experimental data for mineral oil with and without SPAN20 surfactant.

| Pure Oil | | | |
| --- | --- | --- | --- |
| Flow rate ratio | Experimental | Correlation | % Relative Error |
| 0.000 | 0.000 | 0.000 | 0.000 |
| 0.100 | 0.323 | 0.320 | 1.086 |
| 0.150 | 0.3734 | 0.372 | 0.540 |
| 0.200 | 0.415 | 0.413 | 0.386 |
| 0.250 | 0.455 | 0.449 | 1.408 |
|  | | | |
| Oil with SPAN 20 | | | |
| Flow rate ratio | Experimental | Correlation | % Relative Error |
| 0.000 | 0.000 | 0.000 | 0.000 |
| 0.100 | 0.360 | 0.363 | 0.582 |
| 0.150 | 0.427 | 0.421 | 1.363 |
| 0.200 | 0.467 | 0.469 | 0.374 |
| 0.250 | 0.513 | 0.509 | 0.855 |

Similar relative errors are calculated for $f_{2}\left( \theta\right)$ (equation S3) for the experiments performed in the absence and presence of the surfactant.

$f_{2}\left( \theta\right)= \frac{D^{*}}{f_{1}(\varphi)}= \left\{ \begin{aligned} 1.01e^{0.36\theta}, pure Oil \\ \\ 1.05e^{0.27\theta}, Oil with SPAN20 \end{aligned} \right.$ (S3)

**Table S4** Relative error calculated from $f_{2}\left( \theta\right)$ for mineral oil with and without SPAN 20

| Pure Oil | | | |
| --- | --- | --- | --- |
| $f_{2}\left( \theta\right)$ | Experimental | Correlation | % Relative Error |
| 0.000 | 1.031 | 1.010 | 2.037 |
| 0.230 | 1.128 | 1.097 | 2.710 |
| 0.385 | 1.171 | 1.160 | 0.964 |
| 0.538 | 1.213 | 1.226 | 1.097 |
|  | | | |
| Oil with SPAN 20 | | | |
| $f_{2}\left( \theta\right)$ | Experimental | Correlation | % Relative Error |
| 0.000 | 0.978 | 1.046 | 6.870 |
| 0.230 | 1.143 | 1.112 | 2.665 |
| 0.385 | 1.219 | 1.159 | 4.923 |
| 0.538 | 1.247 | 1.207 | 3.152 |
| 0.692 | 1.266 | 1.258 | 0.599 |
| 0.846 | 1.294 | 1.311 | 1.335 |
| 1.000 | 1.318 | 1.366 | 3.656 |

**S5. Lumped system calculations**

Heat transfer analysis was carried out based on equation S4 to predict the time required for droplets to reach the temperature of the heater and compare it with the time calculated from simulations.

$\frac{T\left( t \right)-T_{\infty}}{{T_{i}-T}_{\infty}}= e^{-\left[ \frac{hA}{\rho\forall C} \right]t}$ (S6)

where $T_{\infty}$ is oil temperature, $T_{i}$ is droplet initial temperature, $h$ is convective heat transfer coefficient, $A$ is drop surface area, $\forall$ is droplet volume, $\rho$ and $C$ are droplet density and specific heat, respectively. The time required to heat up the droplet is inversely proportional to the droplet diameter.

Nusselt number (Nu):

Nu = 3.675

Convective heat transfer coefficient (h)

$h= \frac{k Nu}{D_{Hy}}$ (S7)

where $k$ is thermal conductivity in (W/m.K) and $D_{Hy}$ is hydraulic diameter.

| 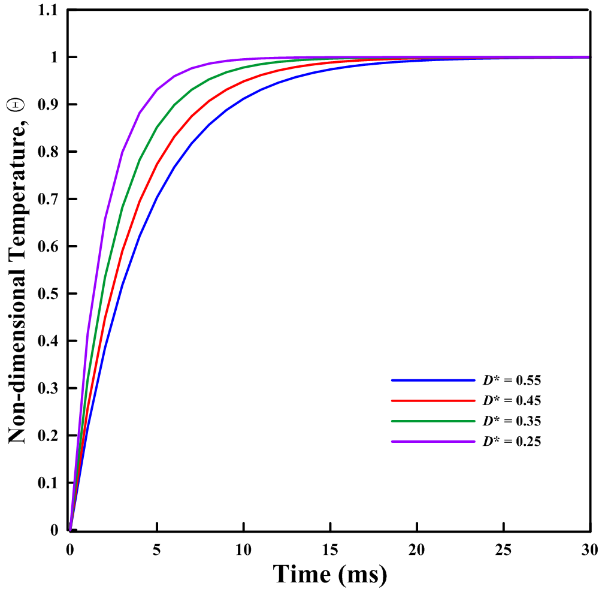  **Figure S2** Dimensionless temperature (θ) as a function of heating time at different droplets sizes. |
| --- |

## S4. Detailed numerical results


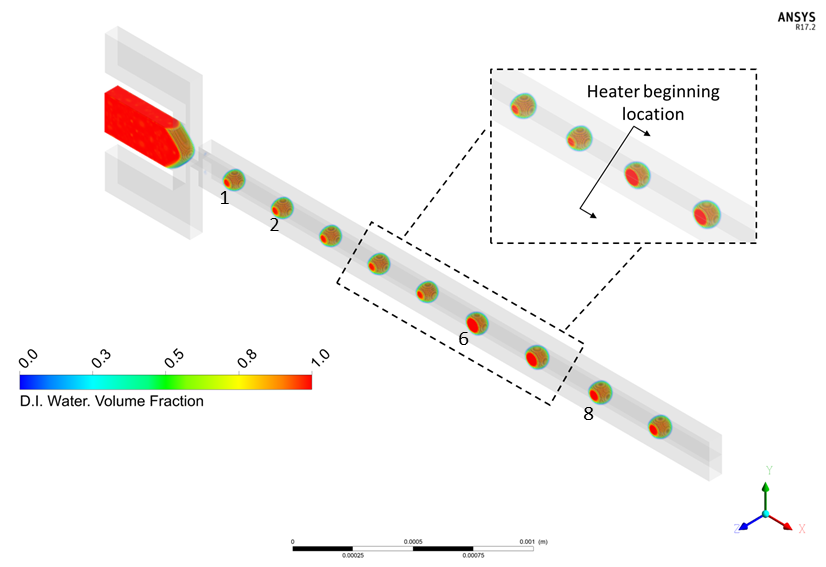


**Figure S3** Simulation results at the flow rate ratio of 0.2. The density, viscosity, and surface tensions are functions of temperature (contact angle was assumed to be constant due to the lack of literature data on its dependency to temperature). The droplet volume increases gradually during its transport over the heater in response to a gradual increase of temperature inside the channel and then stabilizes where it reaches the stable temperature zone of the heater. when the density and viscosity of both phases are functions of temperature, the droplets moved towards the heater because of thermocapillary effect as well. The XY-diameter appears larger at the transit region (a thermal gradient within the droplet) (droplet no. 6 from generation point). Although droplet no. 6 seems having a larger diameter than no. 8. The volume of the droplet is not constant (the number of the cells occupied by droplet no. 8 is bigger than the number of the cells occupied by droplets 1,2, and 6). Fig. 8a was obtained at an iso-surface with z= 40 µm, total value of z=80 µm, starting at z=0).


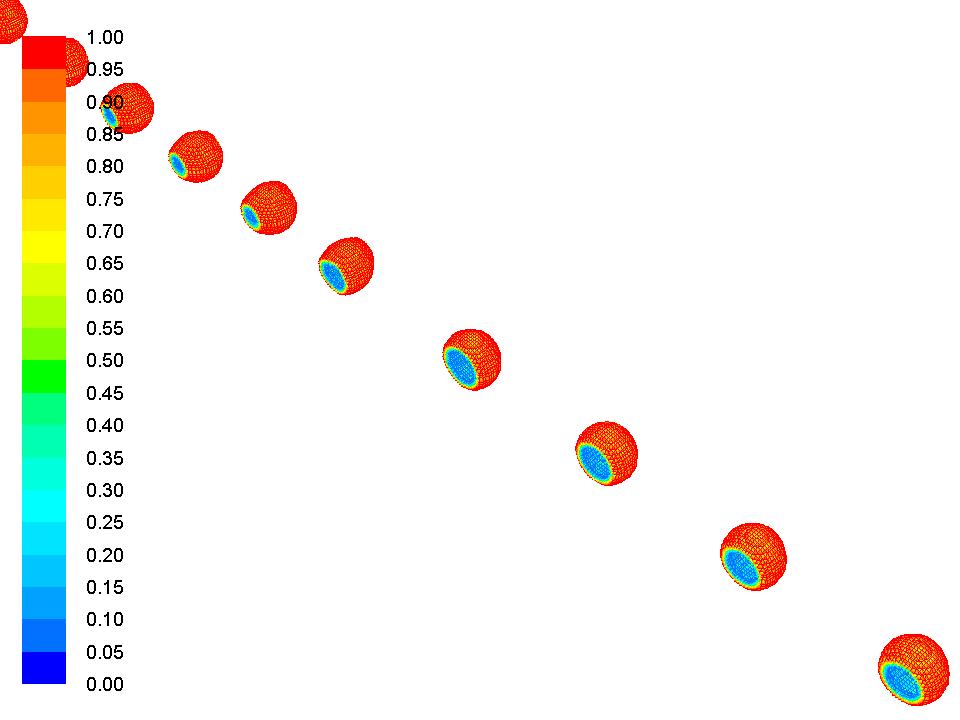


**Figure S4** The zoomed view of the gradual increase of the droplet volume over the heater (shown in **Fig S3**). The density, viscosity, and surface tensions are defined as functions of temperature.

**Figure S5** The simulation results for the gradual increase of the droplet volume (quantified by the number of cells per droplet) through its travel over the heater.


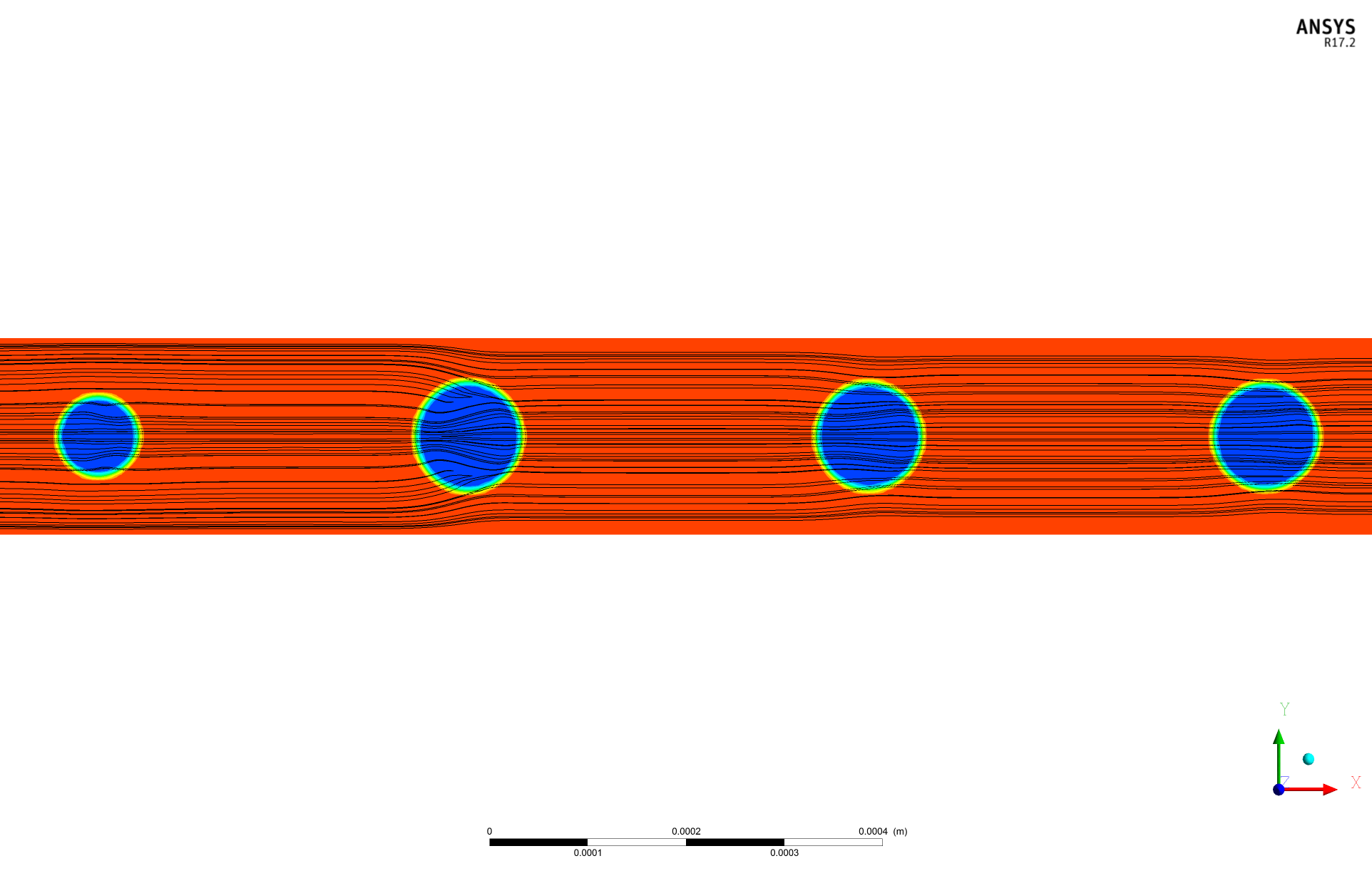


(a)


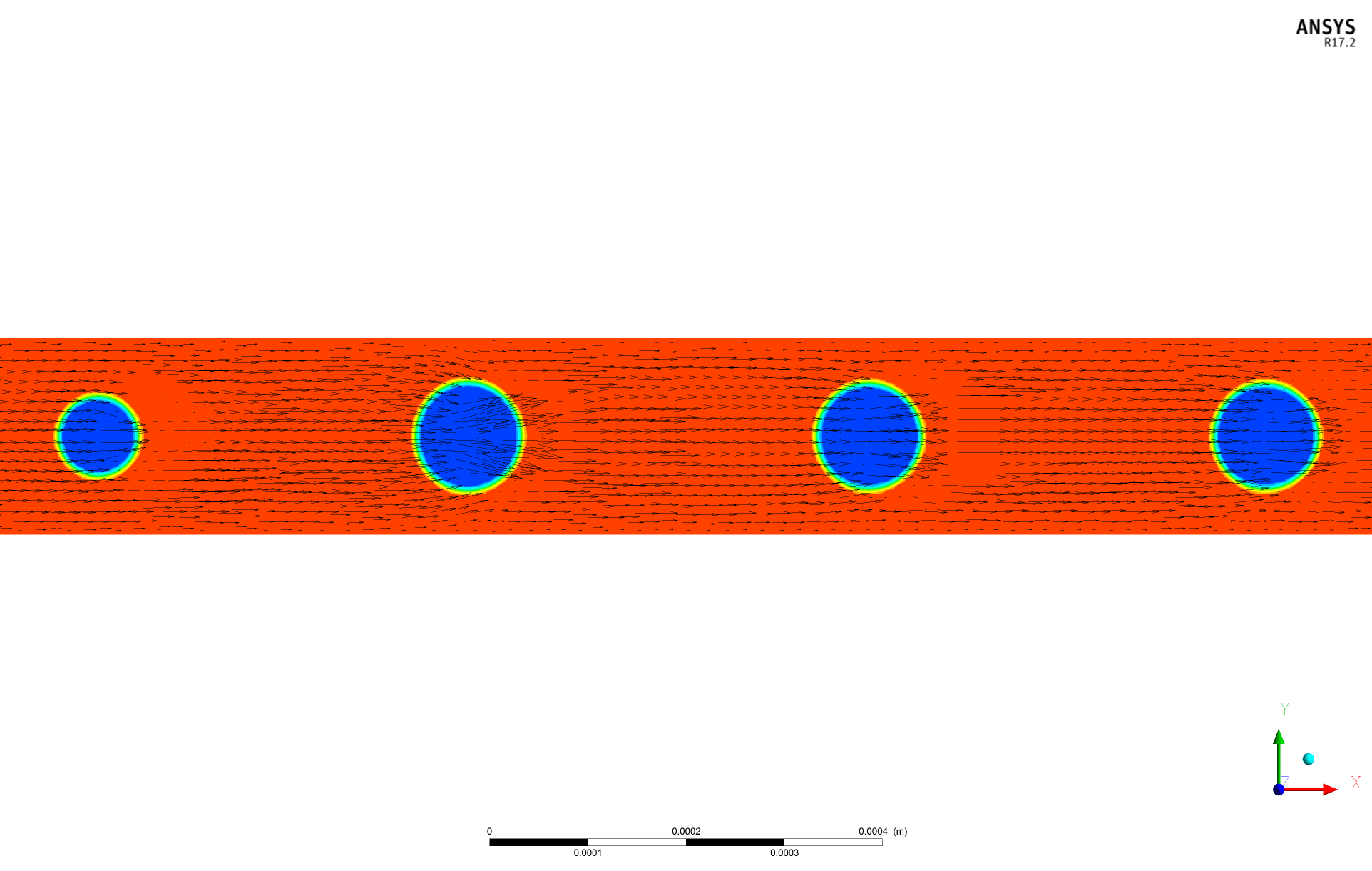


(b)

**Figure S6** Volume fraction profile for droplets #2, 3, 4, and 5 with (a) streamlines of velocity and (b) velocity vectors.


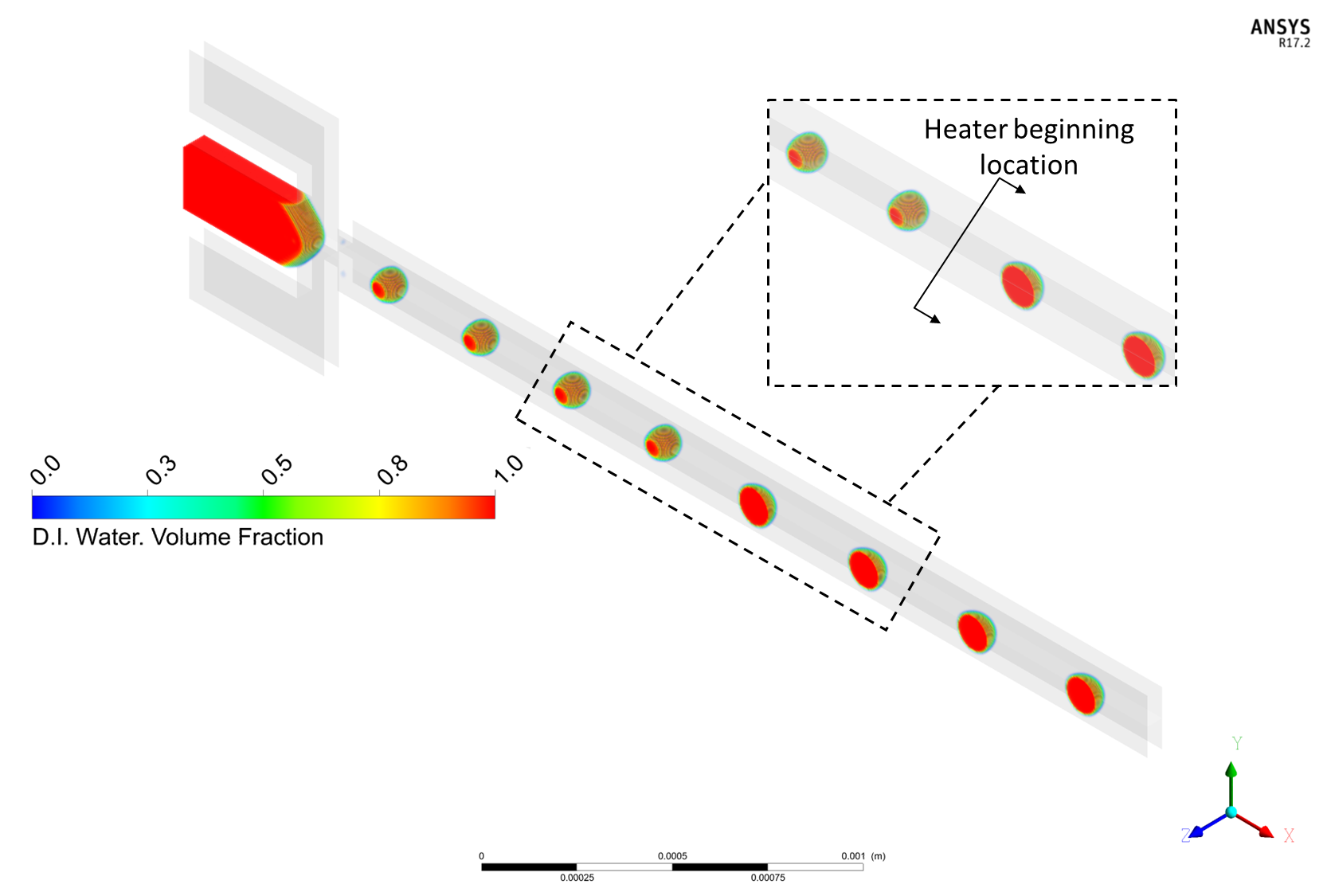


**Figure S7** Surface tension is the only temperature-dependant property in temperature-dependent droplet transport under external heating source (The density and viscosity of both phases are assumed constant).
